# Gene Structures, Evolution, Classification and Expression Profiles of the Aquaporin Gene Family in Castor Bean (*Ricinus communis* L.)

**DOI:** 10.1371/journal.pone.0141022

**Published:** 2015-10-28

**Authors:** Zhi Zou, Jun Gong, Qixing Huang, Yeyong Mo, Lifu Yang, Guishui Xie

**Affiliations:** 1 Danzhou Investigation & Experiment Station of Tropical Crops, Ministry of Agriculture, Rubber Research Institute, Chinese Academy of Tropical Agricultural Sciences, Danzhou, Hainan, P. R. China; 2 Institute of Tropical Biosciences and Biotechnology, Chinese Academy of Tropical Agricultural Sciences, Haikou, Hainan, P. R. China; Universidade Federal do Rio Grande do Sul, BRAZIL

## Abstract

Aquaporins (AQPs) are a class of integral membrane proteins that facilitate the passive transport of water and other small solutes across biological membranes. Castor bean (*Ricinus communis* L., Euphobiaceae), an important non-edible oilseed crop, is widely cultivated for industrial, medicinal and cosmetic purposes. Its recently available genome provides an opportunity to analyze specific gene families. In this study, a total of 37 full-length AQP genes were identified from the castor bean genome, which were assigned to five subfamilies, including 10 plasma membrane intrinsic proteins (PIPs), 9 tonoplast intrinsic proteins (TIPs), 8 NOD26-like intrinsic proteins (NIPs), 6 X intrinsic proteins (XIPs) and 4 small basic intrinsic proteins (SIPs) on the basis of sequence similarities. Functional prediction based on the analysis of the aromatic/arginine (ar/R) selectivity filter, Froger’s positions and specificity-determining positions (SDPs) showed a remarkable difference in substrate specificity among subfamilies. Homology analysis supported the expression of all 37 RcAQP genes in at least one of examined tissues, e.g., root, leaf, flower, seed and endosperm. Furthermore, global expression profiles with deep transcriptome sequencing data revealed diverse expression patterns among various tissues. The current study presents the first genome-wide analysis of the AQP gene family in castor bean. Results obtained from this study provide valuable information for future functional analysis and utilization.

## Introduction

Aquaporins (AQPs) are a special class of integral membrane proteins that belong to the ancient major intrinsic protein (MIP) superfamily [1,2]. Although they firstly raised considerable interest for their high permeability to water, increasing evidence has shown that some of them also transport certain small molecules, e.g., glycerol, urea, boric acid, silicic acid, ammonia (NH_3_), carbon dioxide (CO_2_) and hydrogen peroxide (H_2_O_2_) [3,4]. After the first AQP gene reported in human erythrocytes [5], in the past three decades, its homologs have been identified from all types of organisms, including eubacteria, archaea, fungi, animals and plants [2,4]. The AQPs are characterized by six transmembrane helices (TM1–TM6) connected by five loops (i.e. LA–LE) as well as two highly conserved NPA motifs located at the N-termini of two half helices (i.e. HB and HE) in LB and LE. The NPA motifs which create an electrostatic repulsion of protons and act as a size barrier form one selectivity region of the pore, whereas another region called the aromatic/arginine (ar/R) selectivity filter (i.e. H2 in TM2, H5 in TM5, LE1 and LE2 in LE) that renders the pore constriction site diverse in both size and hydrophobicity determines the substrate specificity [6–8]. Based on the statistical analysis, Froger et al. proposed five conserved amino acid residues (called Froger’s positions, P1–5) for discriminating glycerol-transporting aquaglyceroporins (GLPs) from water-conducting AQPs: GLPs usually feather an aromatic residue at P1, an acidic residue at P2, a basic residue at P3, a proline followed by a nonaromatic residue at P4 and P5, as Y^108^-D^207^-K^211^-P^236^-L^237^ observed in the *Escherichia coli* glycerol facilitator GlpF in contrast to A^103^-S^190^-A^194^-F^208^-W^209^ in the pure water channel AqpZ [9]. More recently, based on the analysis of structure resolved and/or functionally characterized AQPs, nine specificity-determining positions (SDPs) for non-aqua substrates, i.e., urea, boric acid, silicic acid, NH_3_, CO_2_ and H_2_O_2_ were also predicted for each group [10]. Surveys at the global genome level indicate that the AQPs in terrestrial plants are especially abundant and diverse [11–15]. Based on sequence similarities, plant AQPs are divided into seven main subfamilies, i.e., plasma membrane intrinsic proteins (PIPs), tonoplast intrinsic proteins (TIPs), NOD26-like intrinsic proteins (NIPs), small basic intrinsic proteins (SIPs), uncategorized X intrinsic proteins (XIPs), GlpF-like intrinsic proteins (GIPs) and hybrid intrinsic proteins (HIPs) [16–19]. The former four subfamilies are widely distributed whereas GIPs and HIPs are only found in algae and moss, and XIPs in moss and several dicots including poplar (*Populus trichocarpa*) [11–20].

Castor bean (*Ricinus communis* L., 2n = 20) is an annual or perennial shrub that belongs to the Euphorbiaceae family. The castor oil extracted from the seeds is an important raw material used for industrial, medicinal and cosmetic purposes [21]. Although originated in Africa, castor bean is now cultivated in many tropical, subtropical and warm temperate regions around the world, especially under unfavorable conditions with barren, drought and salt stresses [22–24]. The recent completion of its draft genome and the available transcriptome datasets provide an opportunity to analyze specific gene families in castor bean [25–32]. In present study, a genome-wide search was performed to identify the castor bean AQP (RcAQP) genes. Furthermore, functional prediction was performed based on the ar/R filter, Froger’s positions and SDPs [6,7,9], and the expression profiles were examined using deep transcriptome sequencing data. Results obtained from this study provide global information in understanding the molecular basis of the AQP gene family in castor bean.

## Methods

### Identification of RcAQP Genes

The genome sequences of castor bean [25] were downloaded from phytozome v9.1 (http://www.phytozome.net/), whereas the nucleotides, Sanger ESTs (expressed sequence tags) and raw RNA sequencing reads were downloaded from NCBI (http://www.ncbi.nlm.nih.gov/). The amino acid sequences of Arabidopsis (*Arabidopsis thaliana*) and poplar AQP obtained from Phytozome v9.1 (the accession numbers are available in S1 Table) were used as queries to search for castor bean AQP homologs. Sequences with an E-value of less than 1E-5 in the tBlastn search [33] were selected for further analysis. The predicted gene models were further validated with cDNAs, ESTs and raw RNA sequencing reads derived from various tissues/organs such as root, stem, leaf, flower, seed, embryo, endosperm and callus [25–32]. Gene structures were displayed using GSDS [34]. Homology search for nucleotides, ESTs or RNA sequencing reads was performed using Blastn [33], and sequences with a similarity of more than 98% were taken into account.

### Sequence Alignments and Phylogenetic Analysis

Multiple sequence alignments using deduced amino acid sequences were performed with ClustalX [35], and the unrooted phylogenetic tree was constructed by the maximum likelihood method using MEGA 6.0 [36]. The reliability of branches in resulting trees was supported with 1,000 bootstrap resamplings. Classification of AQPs into subfamilies and subgroups was done as described before [16,19].

### Structural Features of Putative RcAQPs

Biochemical features of RcAQPs were determined using ProtParam (http://web.expasy.org/protparam/). The protein subcellular localization was predicted using WoLF PSORT [37] and Plant-mPLoc [38]. The transmembrane regions were detected using TOPCONS [39]. Functional prediction was performed on the basis of dual NPA motifs, ar/R filter (H2, H5, LE1 and LE2), Froger’s positions (P1–5) and specificity-determining positions (SDP1–9) from alignments with the structure resolved *Spinacia oleracea* PIP2;1 and functionally characterized AQPs [8–10].

### Gene Expression Analyses

To analyze the global expression profiles of RcAQP genes among different tissues or developmental stages, RNA sequencing data of leaf, flower, endosperm (II/III, V/VI) and seed described before [30] were examined: the expanding true leaves, appearing after the first cotyledons and leaf-pair, represent the leaf tissue; the male flower tissue includes pollen and anthers but excludes sepals; the germinating seed tissue was obtained by soaking dry seeds in running water overnight followed by germination in the dark for 3 days; and the endosperm tissue includes two representative stages termed stages II/III (endosperm free-nuclear stage) and V/VI (onset of cellular endosperm development). The clean reads were obtained by removing adaptor sequences, adaptor-only reads, reads with “N” rate larger than 10% (“N” representing ambiguous bases) and low quality reads containing more than 50% bases with Q-value≤5. Then, the clean reads were mapped to 37 identified RcAQP genes (cDNA) and released transcripts using Bowtie 2 [40], and the mapped reads were counted. The abundance of each transcript can be measured by its read counts. To remove technical biases inherent in the sequencing approach, the widely-used RPKM (reads per kilo bases per million reads) method [41], which was developed to correct certain biases (i.e. the length of the RNA species and the sequencing depth of a sample), was adopted for the expression annotation. Unless specific statements, the tools used in this study were performed with default parameters.

## Results

### Identification and Classification of RcAQP Genes

Via a comprehensive homology analysis, a total of 37 loci putatively encoding AQP-like genes were identified from the castor bean genome, corresponding 36 loci by the genome annotation (Table 1) [25]. Among them, the locus 28747.t000001 was predicted to encode 243 residues (28747.m000131) with a gene length of 11054 bp (default in part of the 3’ sequences), however, EST and read mapping supported the existence of two genes denoted *RcXIP2;1* and *RcXIP3;1*, both of which harbor one intron and putatively encode 306 and 304 residues, respectively (see S1 File). In addition, although most predicted gene models were validated with ESTs and RNA sequencing reads, three loci (i.e. 28962.t000006, 30101.t000004 and 29816.t000013) seem not to be properly annotated. In phytozome v9.1, the locus 28962.t000006 was predicted to harbor two introns encoding 198 residues (28962.m000437) which is relatively shorter than that of any other PIP subfamily members, however, two nucleotide sequences (accession numbers HB466472 and HB466473) [42] and three ESTs (accession numbers EE257493, EE260412 and EE258867) suggested that this locus harbors four introns encoding 270 or less residues depending on different alternative splicing isoforms (at least four) (see S2 File), and the longest transcript consistent with other *PIPs* was selected for further analyses. The locus 30101.t000004 was predicted to encode 203 residues (30101.m000372), however, EST mapping indicated that a number of 143-bp coding -sequences close to the second intron is absent from the assembled genome, which was further validated with PCR amplification and sequencing (see S3 File). The locus 29816.t000013 was predicted to harbor seven introns encoding 367 residues (29816.m000676) which is considerably longer than that of any other NIPs, however, thousands of RNA sequencing reads indicated that this locus harbors only four introns putatively encoding 269 residues (see S4 File). Although the current draft genome of castor bean is comprised of 25,763 scaffolds without anchored to 10 chromosomes [25], we still observed that 8 scaffolds harbor two AQP-encoding loci, whereas other 21 scaffolds contain only one (Table 1).

**Table 1:** List of the 37 RcAQP genes identified in this study.

Name	Phytozome position	Locus ID	Transcript ID	Identified position	Nucleotide length (bp, from start to stop codons)		Intron NO.	EST hits in GenBank	Alternative splicing	Phytozome ID of AtAQP ortholog	Phytozome ID of PtAQP ortholog
					Gene	CDS					
*RcPIP1;1*	29969: 73673–75042	29969.t000006	29969.m000266	29969: 73604–75299	1370	867	3	0	-	AT4G00430	Pt_0010s19930
*RcPIP1;2*	29669: 103956–102272	29669.t000017	29669.m000808	29669: 104366–101932	1304	864	3	18	-	AT4G00430	Pt_0003s12870
*RcPIP1;3*	29669: 109461–107998	29669.t000018	29669.m000809	29669: 109461–107553	1243	867	3	14	-	AT4G00430	Pt_0003s12870
*RcPIP1;4*	30190: 2676316–2679326	30190.t000465	30190.m011229	30190: 2676127–2679732	2648	864	3	109	Yes	AT4G00430	Pt_0003s12870
*RcPIP1;5*	30174: 1747792–1749693	30174.t000011	30174.m008614	30174: 1747452–1750093	1712	861	3	10	-	AT4G00430	Pt_0008s06580
*RcPIP2;1*	30078: 847025–848540	30078.t000130	30078.m002337	30078: 846884–849450	1185	867	3	155	Yes	AT2G37170	Pt_0008s03950
*RcPIP2;2*	27516: 79785–82924	27516.t000007	27516.m000174	27516: 79695–83007	2665	852	3	14	Yes	AT2G37170	Pt_0010s22950
*RcPIP2;3*	28076: 21501 23698	28076.t000002	28076.m000411	28076: 21259–23855	1939	864	3	2	-	AT5G60660	Pt_0009s01940
*RcPIP2;4*	29869: 700906–699306	29869.t000059	29869.m001194	29869: 701298–698970	1221	843	3	35	Yes	AT2G16850	Pt_0004s18240
*RcPIP2;5*	28962: 25218–23278	28962.t000006	28962.m000437	28962: 25344–23278	1654	813	4	3	Yes	AT4G35100	Pt_0005s11110
*RcTIP1;1*	30078: 302469–303701	30078.t000047	30078.m002254	30078: 302469–304194	871	756	1	307	-	AT2G36830	Pt_0006s12350
*RcTIP1;2*	28180: 101873–103187	28180.t000017	28180.m000392	28180: 101668–103298	1149	759	2	5	-	AT4G01470	Pt_0001s24200
*RcTIP1;3*	29788: 135388–136721	29788.t000021	29788.m000338	29788: 135388–137224	1070	759	2	60	-	AT4G01470	Pt_0001s24200
*RcTIP1;4*	29589: 301876–300570	29589.t000040	29589.m001261	29589: 301877–300492	1129	759	1	7	-	AT3G26520	Pt_0008s05050
*RcTIP2;1*	30146: 893208–891669	30146.t000119	30146.m003542	30146: 893065–891669	1300	747	2	47	Yes	AT3G16240	Pt_0003s04930
*RcTIP2;2*	30101: 54727–56622	30101.t000004	30101.m000372	30101: 54578–56981	1059	753	2	196	Yes	AT3G16240	Pt_0001s15700
*RcTIP3;1*	29681: 356771–358102	29681.t000071	29681.m001366	29681: 356430–358852	994	768	2	68	Yes	AT1G17810	Pt_0017s03540
*RcTIP4;1*	29794: 784441–783217	29794.t000118	29794.m003419	29794: 784479–783045	1017	744	2	1	-	AT2G25810	Pt_0006s25620
*RcTIP5;1*	30147: 1084776–1086016	30147.t000502	30147.m014231	30147: 1083709–1086062	1241	759	2	0	-	AT3G47440	Pt_0001s00690
*RcNIP1;1*	30026: 383318–385396	30026.t000052	30026.m001488	30026: 382992–385763	2079	816	4	1	-	AT4G18910	Pt_0004s06160
*RcNIP2;1*	27860: 4197–6794	27860.t000001	27860.m000040	27860: 3885–7450	2598	894	4	0	-	-	Pt_0017s11960
*RcNIP3;1*	29908: 1225139–1223830	29908.t000084	29908.m006033	29908: 1225139–1222967	1310	849	4	0	-	-	Pt_0002s09740
*RcNIP4;1*	29816: 132753–129974	29816.t000013	29816.m000676	29816: 132884–130426	1425	810	4	0	-	AT5G37810	Pt_0010s12330
*RcNIP4;2*	28827: 15568–18585	28827.t000002	28827.m000171	28827: 15568–18585	3018	759	4	0	-	AT5G37820	Pt_0017s03060
*RcNIP5;1*	30068: 792510–798491	30068.t000130	30068.m002640	30068: 792230–798545	4934	897	3	62	-	AT4G10380	Pt_0001s45920
*RcNIP6;1*	29588: 109897–113370	29588.t000015	29588.m000860	29588: 109896–113370	3320	927	4	1	-	AT1G80760	Pt_0003s17930
*RcNIP7;1*	29844: 994348–995605	29844.t000176	29844.m003330	29844: 993874–996212	1258	897	4	0	-	AT3G06100	Pt_0008s20750
*RcXIP1;1*	28929: 27007–26856	28929.t000003	28929.m000055	28929: 27059–25434	1195	930	1	20	-	-	Pt_0009s13090
*RcXIP1;2*	28846: 5121–3392	28846.t000001	28846.m000048	28846: 5267–3386	1346	912	1	12	-	-	Pt_0009s13090
*RcXIP1;3*	28846: 37430–36553	28846.t000002	28846.m000049	28846: 38157–36376	858	858	0	3	-	-	Pt_0009s13090
*RcXIP1;4*	28929: 16870–16144	28929.t000002	28929.m000054	28929: 16870–16144	727	627	1	0	-	-	Pt_0009s13090
*RcXIP2;1*	28747: 53725–42672	28747.t000001	28747.m000131	28747: 54568–52108	1302	921	2	18	Yes	-	Pt_0009s13110
*RcXIP3;1*				28747: 44459–42629	1827	915	1	0	-	-	Pt_0004s17430
*RcSIP1;1*	29950: 345324–340111	29950.t000061	29950.m001177	29950: 345535–339806	4822	720	2	4	-	AT3G04090	Pt_0019s04640
*RcSIP1;2*	30010: 75574–74870	30010.t000008	30010.m000658	30010: 76324–74304	705	705	0	0	-	AT3G04090	Pt_0014s15250
*RcSIP1;3*	30010: 77831–77112	30010.t000009	30010.m000659	30010: 78002–76765	720	720	0	0	-	AT3G04090	Pt_0014s15250
*RcSIP2;1*	30045: 458397–454436	30045.t000019	30045.m000487	30045: 458594–454223	3962	723	2	0	-	AT3G56950	Pt_0016s02560

**Fig 1:**
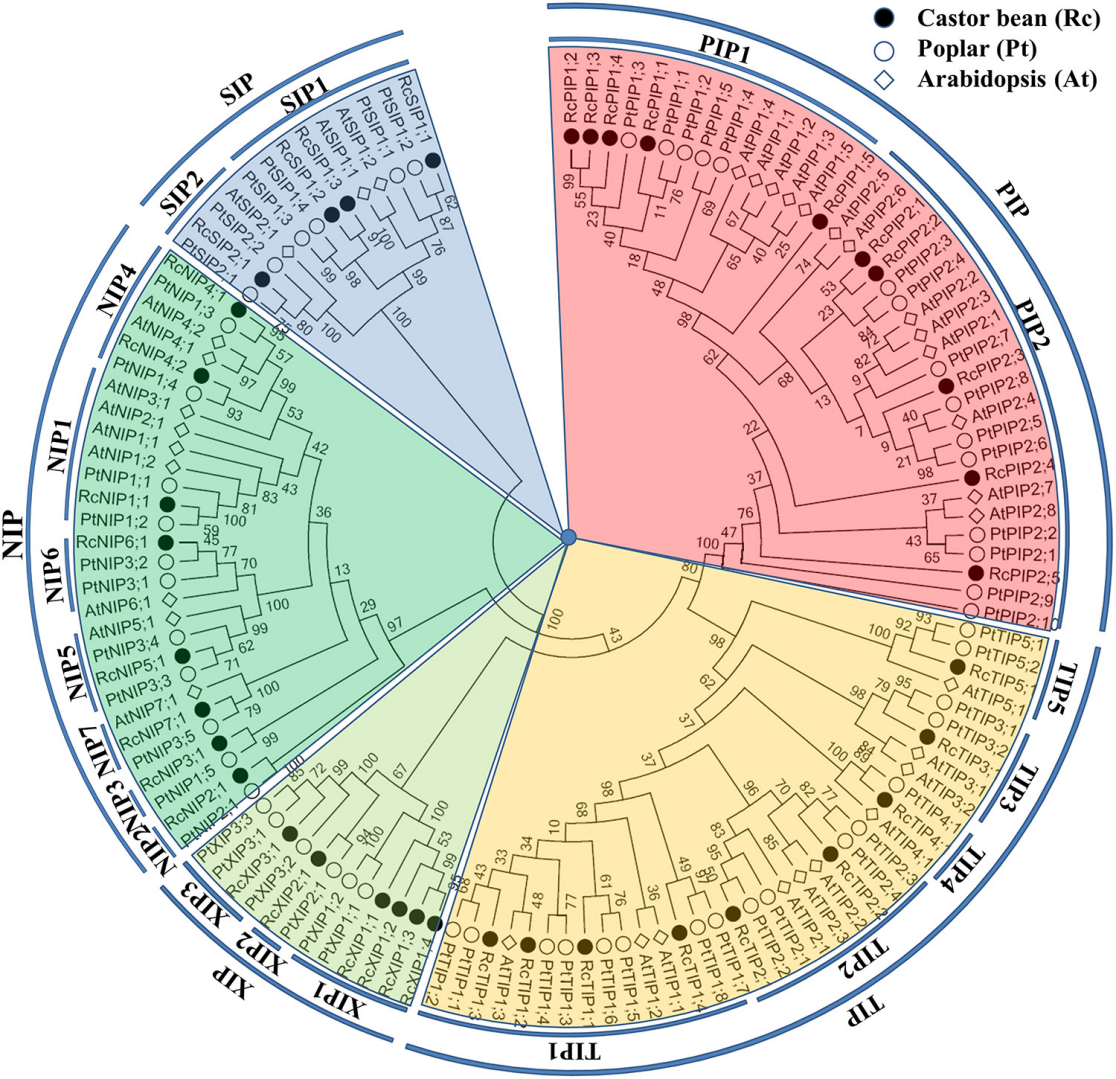
Phylogenetic analysis of deduced amino acid sequences of the 37 RcAQPs with Arabidopsis and poplar homologs. Deduced amino acid sequences were aligned using ClustalX and the phylogenetic tree was constructed using bootstrap maximum likelihood tree (1000 replicates) method and MEGA6 software. The distance scale denotes the number of amino acid substitutions per site. The name of each subfamily and subgroup is indicated next to the corresponding group. Species and accession numbers are listed in Table 1 and S1 Table.

To analyze the evolutionary relationship and their putative function, an unrooted phylogenetic tree was constructed from the deduced amino acid sequences of RcAQPs together with that from model plant species, Arabidopsis (AtAQPs) [16] and poplar (PtAQPs) [19] (Fig 1). According to the phylogenetic analysis, the identified RcAQPs were grouped into five subfamilies, i.e., PIP (10), TIP (9), NIP (8), XIP (6) and SIP (4) (Table 1 and Fig 1). Following the nomenclature of Arabidopsis and poplar [16,19], the RcPIP subfamily was further divided into two phylogenetic subgroups (5 RcPIP1s and 5 RcPIP2s), the RcTIP subfamily into five subgroups (4 RcTIP1s, 2 RcTIP2s, 1 RcTIP3, 1 RcTIP4 and 1 RcTIP5), the RcNIP subfamily into seven subgroups (1 RcNIP1, 1 RcNIP2, 1 RcNIP3, 2 RcNIP4s, 1 RcNIP5, 1 RcNIP6 and 1 RcNIP7), the RcSIP subfamily into two subgroups (3 RcSIP1s and 1 RcSIP2) and the RcXIP subfamily into three subgroups (4 RcXIP1s, 1 RcXIP2 and 1 RcXIP3) (Fig 1). Although the closest homolog of RcNIP2;1 and RcNIP3;1 is not AtNIP2;1 or AtNIP3;1, their counterparts in poplar were identified, thereby, they were nominated following the nomenclature of poplar [19]. As shown in Fig 1, several RcAQPs were clustered together apart from homologs from Arabidopsis and poplar, i.e., RcPIP1;2/RcPIP1;3/RcPIP1;4, RcPIP2;1/RcPIP2;2, RcSIP1;2/RcSIP1;3, RcXIP1;1/RcXIP1;2/RcXIP1;3/RcXIP1;4. Most of these gene pairs are tandem distributed on the same scaffold, i.e., RcPIP1;2/RcPIP1;3 on the scaffold29669, RcSIP1;1/RcSIP1;2 on the scaffold30010, RcXIP1;1/RcXIP1;4 on the scaffold28929 and RcXIP1;2/RcXIP1;3 on the scaffold28846. Without any exception, these four gene pairs are characterized by same-direction neighbors (foot-to-head order), and the intergenic spacer is about 3.1 kb, 0.5 kb, 8.5 kb or 31.1 kb, respectively (Table 1). Homology analysis indicated that the 37 RcAQPs have 30 counterparts in poplar, whereas only 29 out of them have orthologs with a number of twenty in Arabidopsis (Table 1 and Fig 1).

Sequence alignments indicated that the complete cDNA sequences of 18 RcAQP genes (i.e., *RcPIP1;1*–*RcPIP1;5*, *RcPIP2;1*–*RcPIP2;5*, *RcTIP1;1*–*RcTIP1;3*, *RcTIP2;1*, *RcTIP4;1*, *RcNIP1;1*, *RcNIP5;1* and *RcNIP6;1*) were mentioned in three patents and one academic paper [42–45]. Except for RcPIP1;1, RcPIP2;1 and RcTIP1;1 whose water transport activity and expression pattern along the hypocotyl axis were investigated [45], the expression profiles and precise roles of other RcAQP genes have not been reported yet. Homology search showed that 25 RcAQP genes had EST hits in NCBI GenBank database (as of Oct 2014), and *RcTIP1;1* matched the maximum number of 304 ESTs. Furthermore, read alignments against RNA sequencing data of root, leaf, flower, seed and endosperm further supported the expression of other 12 RcAQP genes. In addition, alternative splicing isoforms existing in 9 RcAQP-encoding loci were supported by Sanger ESTs (Table 1).

### Analysis of Exon-Intron Structure

**Fig 2:**
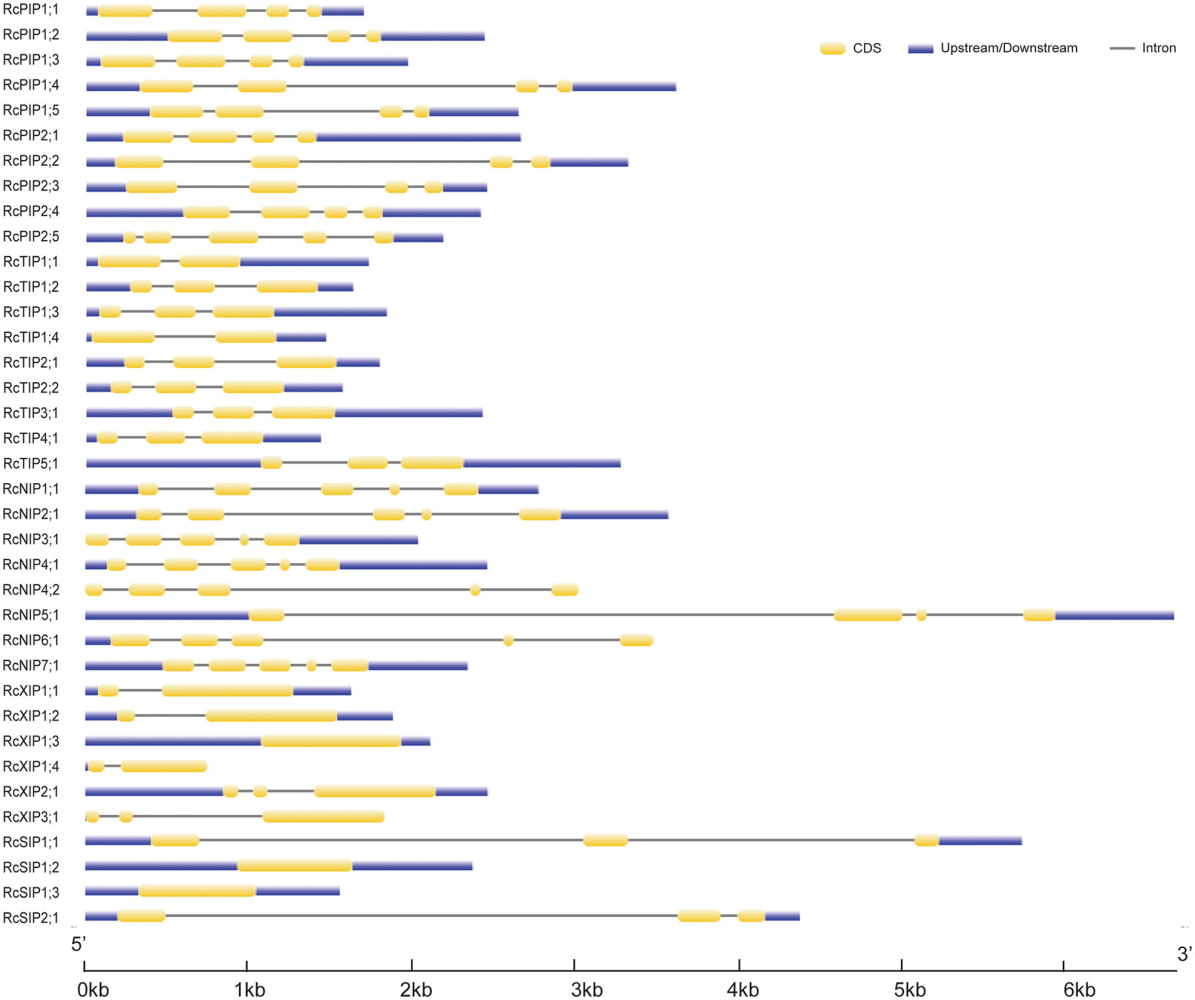
Exon-intron structures of the 37 RcAQP genes. The graphic representation of the gene models is displayed using GSDS.

The exon-intron structures of the 37 RcAQP genes were analyzed based on the optimized gene models. Although the ORF (open reading frame) length of each gene is similar (627–830 bp), the gene size (from start to stop codons) is distinct (705–4934 bp) (Table 1 and Fig 2). The introns of RcAQP genes have an average length of about 380 bp, with the minimum of 46 bp in *RcPIP2;5* and the maximum of 3360 bp in *RcNIP5;1*. Genes in different subfamilies harbor distinct exon-intron structures. Except for the existing of one more small intron close to the 5’-terminal of *RcPIP2;5*, other RcPIP subfamily members feature three introns (74–537 bp, 90–1401 bp and 68–141 bp, respectively). Most *RcTIPs* contain two introns (117–401 bp and 81–429 bp, respectively), in contrast, *RcTIP1;1* and *RcTIP1;4* contain only one. Most *RcNIPs* harbor four introns (91–343 bp, 82–910 bp, 86–1469 bp and 91–650 bp, respectively), however, *RcNIP5;1* contains three instead. Two out of three *RcSIP1s* harbor no introns, in contrast, *RcSIP1;1* and *RcSIP2;1* contain two. Most *RcXIPs* contain one intron except for *RcXIP1;4* and *RcXIP2;1* that contain zero or two, respectively (Table 1 and Fig 2).

### Structural Features of RcAQPs

Sequence analysis showed that the 37 deduced RcAQPs consist of 208–309 amino acids, with a theoretical molecular weight of 22.49–33.22 kDa and a *p*I value of 4.93–9.97. Homology analysis of these deduced proteins revealed a high sequence diversity existing within and between the five subfamilies. The sequence similarity of 65.2∓99.7% was found within RcPIPs, 57.3∓93.3% within RcTIPs, 37.6∓90.6% within RcXIPs, 33.8∓74.6% within RcNIPs, and 39.4∓75.1% within RcSIPs. RcPIPs share the highest sequence similarity of 37.1∓45.1% with RcTIPs, 25.6∓42.1% with RcXIPs, 22.8∓34.9% with RcNIPs, and the lowest of 23.7∓30.1% with RcSIPs. RcTIPs show 27.2∓42.8%, 20.4∓36.4% and 27.4∓37.3% sequence similarity with RcNIPs, RcXIPs and RcSIPs, respectively. RcNIPs share a similarity of 16.6∓34.5% and 19.3∓30.7% with RcXIPs and RcSIPs, whereas RcSIPs share the similarity of 17.7∓27.9% with RcXIPs (see S2 Table).

Topological analysis showed that almost all RcAQPs were predicted to harbor six TMs except for RcXIP1;4 containing only four, which is consistent with the results from multiple alignments with structure proven AQPs (Table 2 and S5 File). The subcellular localization of each RcAQP was also predicted (Table 2). RcPIPs with an average *p*I value of 7.89 and RcNIPs with an average *p*I value of 8.44 are localized to plasma membranes. RcTIPs with an average *p*I value of 5.63 are mainly localized to vacuoles, though several members (RcTIP3;1 and RcTIP4;1) were mispredicted to target the cytosol by WoLF PSORT. RcSIPs (with an average *p*I value of 9.79) and RcXIPs (with an average *p*I value of 7.63) are mainly predicted to target the plasma membrane by Plant-mPLoc, in contrast, the predicted localizations by WoLF PSORT are diverse, including the plasma membrane, chloroplast, vacuole, peroxisome and cytosol. In addition, based on the multiple alignments with structure/function characterized AQPs, the conserved residues typical of dual NPA motifs, the ar/R selectivity filter, five Froger’s positions and nine SDPs were also identified (Tables 2 and 3, and S6 File).

**Table 2:** Structural and subcellular localization analysis of the RcAQPs.

Name	Len	Mw (KDa)	pI	TM^a^	Loc^b^	Loc^c^	Ar/R selectivity filter				NPA motifs		Froger’s positions				
							H2	H5	LE1	LE2	LB	LE	P1	P2	P3	P4	P5
RcPIP1;1	288	30.77	7.00	6	Plas	Plas	F	H	T	R	NPA	NPA	E	S	A	F	W
RcPIP1;2	287	30.69	7.69	6	Plas	Plas	F	H	T	R	NPA	NPA	E	S	A	F	W
RcPIP1;3	288	30.78	7.65	6	Plas	Plas	F	H	T	R	NPA	NPA	E	S	A	F	W
RcPIP1;4	287	30.67	8.30	6	Plas	Plas	F	H	T	R	NPA	NPA	E	S	A	F	W
RcPIP1;5	286	30.71	8.83	6	Plas	Plas	F	H	T	R	NPA	NPA	Q	S	A	F	W
RcPIP2;1	288	30.66	8.21	6	Plas	Plas	F	H	T	R	NPA	NPA	Q	S	A	F	W
RcPIP2;2	283	30.30	8.29	6	Plas	Plas	F	H	T	R	NPA	NPA	Q	S	A	F	W
RcPIP2;3	287	30.60	7.62	6	Plas	Plas	F	H	T	R	NPA	NPA	Q	S	A	F	W
RcPIP2;4	280	29.71	8.99	6	Plas	Plas	F	H	T	R	NPA	NPA	M	S	A	F	W
RcPIP2;5	270	28.56	6.29	6	Plas	Plas	F	H	T	R	NPA	NPA	M	S	A	F	W
RcTIP1;1	251	25.90	5.91	6	Vacu	Vacu	H	I	A	V	NPA	NPA	T	S	A	Y	W
RcTIP1;2	252	25.92	5.35	6	Vacu	Vacu	H	I	A	V	NPA	NPA	T	S	A	Y	W
RcTIP1;3	252	25.80	4.94	6	Vacu	Vacu	H	I	A	V	NPA	NPA	T	S	A	Y	W
RcTIP1;4	252	26.26	5.13	6	Vacu	Vacu	H	I	A	V	NPA	NPA	T	S	A	Y	W
RcTIP2;1	248	25.03	5.06	6	Vacu	Vacu	H	I	G	R	NPA	NPA	T	S	A	Y	W
RcTIP2;2	250	25.22	4.93	6	Vacu	Vacu	H	I	G	R	NPA	NPA	T	S	A	Y	W
RcTIP3;1	255	27.11	6.49	6	Cyto	Vacu	H	I	A	R	NPA	NPA	T	A	A	Y	W
RcTIP4;1	247	26.15	6.12	6	Cyto	Vacu	H	I	A	R	NPA	NPA	T	S	A	Y	W
RcTIP5;1	252	25.92	6.71	6	Vacu	Vacu	N	V	G	C	NPA	NPA	A	A	A	Y	W
RcNIP1;1	271	28.86	9.26	6	Plas	Plas	W	V	A	R	NPA	NPA	F	S	A	Y	I
RcNIP2;1	297	31.49	9.02	6	Plas	Plas	G	S	G	R	NPA	NPA	L	T	A	Y	I
RcNIP3;1	282	30.65	6.21	6	Plas	Plas	W	A	A	R	NPA	NPA	F	S	A	F	I
RcNIP4;1	269	28.94	8.59	6	Plas	Plas	W	V	A	R	NPA	NPA	F	T	A	Y	M
RcNIP4;2	252	26.39	8.44	6	Plas	Plas	W	V	A	R	NPA	NPA	V	S	A	Y	I
RcNIP5;1	298	30.95	8.88	6	Plas	Plas	A	I	G	R	NPS	NPV	F	T	P	Y	L
RcNIP6;1	308	31.85	8.60	6	Plas	Plas	T	I	A	R	NPS	NPV	F	T	A	Y	L
RcNIP7;1	298	31.57	8.54	6	Plas	Plas	A	V	G	R	NPA	NPA	Y	S	A	Y	I
RcXIP1;1	309	33.22	6.92	6	Cyto	Plas	V	F	V	R	SPT	NPA	M	C	A	F	W
RcXIP1;2	303	32.55	6.91	6	Chlo	Plas	V	F	V	R	SPT	NPA	M	C	A	F	W
RcXIP1;3	285	30.80	7.94	6	Plas	Plas	V	F	V	R	SPA	NPA	M	C	V	F	W
RcXIP1;4	208	22.49	7.57	4	Pero	Plas	V	-	-	-	SPV	-	M	-	-	-	-
RcXIP2;1	306	32.22	8.22	6	Plas	Plas	I	F	V	R	NPV	NPA	V	C	A	F	W
RcXIP3;1	304	32.36	8.21	6	Plas	Plas	V	Y	A	R	NPI	NPA	V	C	A	F	W
RcSIP1;1	239	25.92	9.63	6	Plas	Plas	V	V	P	N	NPT	NPA	E	A	A	Y	W
RcSIP1;2	234	25.24	9.97	6	Vacu	Plas/Vacu	A	A	T	N	NPN	NPA	Q	A	A	Y	W
RcSIP1;3	239	25.62	9.74	6	Plas	Plas/Vacu	A	A	P	N	NPA	NPA	Q	V	A	Y	W
RcSIP2;1	240	26.05	9.82	6	Chlo	Plas/Vacu	S	H	G	S	NPL	NPA	I	V	A	Y	W

Ar/R: aromatic/arginine; Chlo: chloroplast; Cyto: cytosol; ER: endoplasmic reticulum; H2: transmembrane helix 2; H5: transmembrane helix 5; LE: loop E; Loc: subcellular localization; NPA: Asn-Pro-Ala; Plas: plasma membrane; TM: transmembrane helix; Vacu: vacuolar membrane.

^a^ Representing the numbers of transmembrane helices predicted by TOPCONS.

^b^ Best possible subcellular localization prediction by WoLF PSORT.

^c^ Best possible subcellular localization prediction by Plant-mPLoc.

**Table 3:** Summary of typical SDPs and those identified in the RcAQPs ^a^.

SD position Aquaporin	SDP1	SDP2	SDP3	SDP4	SDP5	SDP6	SDP7	SDP8	SDP9
**Typical NH**_**3**_ **transporter**	**F/T**	**K/L/N/V**	**F/T**	**V/L/T**	**A**	**D/S**	**A/H/L**	**E/P/S**	**A/R/T**
RcTIP2;2	T	K	T	V	A	S	A	P	**S**
RcNIP1;1	F	K	F	T	A	D	L	E	T
**Typical boric acid transporter**	**T/V**	**I/V**	**H/I**	**P**	**E**	**I/L**	**I/L/T**	**A/T**	**A/G/K/P**
RcPIP1;1	T	I	H	P	E	L	L	T	P
RcPIP1;2	T	I	H	P	E	L	L	T	P
RcPIP1;3	T	I	H	P	E	L	L	T	P
RcPIP1;4	T	I	H	P	E	L	L	T	P
RcPIP1;5	T	I	H	P	E	L	L	T	P
RcNIP5;1	T	I	H	P	E	L	L	A	P
**Typical CO**_**2**_ **transporter**	**I/L/V**	**I**	**C**	**A**	**I/V**	**D**	**W**	**D**	**W**
RcPIP1;1	V	**M**	C	A	I	D	W	D	W
RcPIP1;3	L	**M**	C	A	I	D	W	D	W
RcPIP1;4	L	**M**	C	A	I	D	W	D	W
RcPIP1;5	I	**M**	C	A	V	D	W	D	W
RcPIP2;2	I	**M**	C	A	V	D	W	D	W
RcPIP2;4	V	**M**	C	A	V	D	W	D	W
**Typical H**_**2**_ **O**_**2**_ **transporter**	**A/S**	**A/G**	**L/V**	**A/F/L/T/V**	**I/L/V**	**H/I/L/Q**	**F/Y**	**A/V**	**P**
RcPIP1;1	A	G	V	L	I	H	F	V	P
RcPIP1;2	A	G	V	F	I	H	F	V	P
RcPIP1;3	A	G	V	F	I	H	F	V	P
RcPIP1;4	A	G	V	F	I	H	F	V	P
RcPIP1;5	A	G	V	F	I	H	F	V	P
RcPIP2;1	A	G	V	F	I	Q	F	V	P
RcPIP2;2	A	G	V	F	I	Q	F	V	P
RcPIP2;3	A	G	V	F	I	Q	F	V	P
RcPIP2;4	A	G	V	F	I	H	F	V	P
RcPIP2;5	A	G	V	F	I	H	F	V	P
RcTIP5;1	S	A	L	A	I	Q	Y	V	P
RcNIP2;1	A	A	L	L	V	I	Y	V	P
RcNIP3;1	S	A	L	L	I	L	F	V	P
RcNIP4;2	S	A	L	V	V	L	Y	V	P
RcNIP5;1	S	A	L	V	V	L	Y	V	P
RcXIP1;1	A	G	L	V	**S**	H	F	V	P
RcXIP1;2	A	G	L	V	**S**	H	F	V	P
RcXIP1;3	A	A	L	V	**S**	H	F	V	P
**Typical silicic acid transporter**	**C/S**	**F/Y**	**A/E/L**	**H/R/Y**	**G**	**K/N/T**	**R**	**E/S/T**	**A/K/P/T**
RcNIP2;1	S	F	**V**	H	G	N	R	T	**Q**
**Typical urea transporter**	**H**	**P**	**F/I/L/T**	**A/C/F/L**	**L/M**	**A/G/P**	**G/S**	**G/S**	**N**
RcPIP1;1	H	P	F	F	L	P	G	G	N
RcPIP1;2	H	P	F	F	L	P	G	G	N
RcPIP1;3	H	P	F	F	L	P	G	G	N
RcPIP1;4	H	P	F	F	L	P	G	G	N
RcPIP1;5	H	P	F	L	L	P	G	G	N
RcPIP2;1	H	P	L	F	L	P	G	G	N
RcPIP2;2	H	P	F	F	L	P	G	G	N
RcPIP2;3	H	P	F	F	L	P	G	G	N
RcPIP2;4	H	P	F	F	L	P	G	G	N
RcPIP2;5	H	P	F	L	L	P	G	G	N
RcTIP1;1	H	P	F	F	L	A	G	S	N
RcTIP1;2	H	P	F	F	L	A	G	S	N
RcTIP1;3	H	P	F	F	L	A	G	S	N
RcTIP1;4	H	P	F	F	L	A	G	S	N
RcTIP2;1	H	P	F	A	L	P	G	S	N
RcTIP2;2	H	P	F	A	L	P	G	S	N
RcTIP3;1	H	P	F	L	L	P	G	S	N
RcTIP4;1	H	P	L	L	L	P	G	S	N
RcTIP5;1	H	P	F	A	L	P	G	S	N
RcNIP1;1	H	P	L	A	L	P	G	S	N
RcNIP2;1	H	P	T	A	M	P	G	S	N
RcNIP3;1	H	P	I	A	L	P	G	S	N
RcNIP4;1	H	P	I	A	L	P	G	S	N
RcNIP4;2	H	P	I	A	L	P	G	S	N
RcNIP5;1	H	P	I	A	L	P	G	S	N
RcNIP6;1	H	P	I	A	L	**E**	G	S	N
RcNIP7;1	H	P	I	A	M	P	G	S	N

^a^ The SDP residues in the castor bean AQPs differing from the typical SDPs determined in this study are highlighted in bold.

### RcPIP Subfamily

All RcPIPs were identified to have similar sequence length, however, RcPIP2s (270–288 residues) can be distinguished from RcPIP1s (286–288 residues) by harboring relatively shorter N-terminal and longer C-terminal sequences (see S5 File). The five RcPIP1s harbor sequence similarities of 90.7∓99.7%, whereas the similarity similarities of the five RcPIP2s are 73.3∓94.5%. Between RcPIP1 and RcPIP2 members, sequence similarities of 59.1∓65.9% were observed (see S1 Table). The dual NPA motifs, ar/R filter (F-H-T-R), and four of five Froger’s positions are highly conserved in RcPIPs (Table 2). In contrast, the P1 position is more variable with the appearance of an E, Q or M residue (Table 2). In addition, two phosphorylation sites corresponding to S115 and S274 in *Spinacia oleracea* PIP2;1 [8] are invariable in RcPIP2s, and the former one is even highly conserved in all RcPIPs, RcTIPs and RcXIPs, and most RcSIP1s except for the S→T substitution in several members (see S5 File), implying their regulation by phosphorylation.

### RcTIP Subfamily

RcTIPs consist of 247–255 residues. Those belonging to RcTIP1s (4 members, 251–252 residues) share 84.6∓93.3% sequence similarities, whereas two RcTIP2s (248–250 residues) exhibit a similarity of 87.2% (Table 2 and S1 Table). Members of the RcTIP1 subgroup exhibit similarities of 71.5∓76.6%, 70.0∓72.7%, 67.1∓69.7% and 60.7∓63.0% with that of RcTIP2, RcTIP3, RcTIP4 and RcTIP5 subgroups, respectively. RcTIP2s share similarities of 69.1∓71.0%, 68.3∓70.1% and 65.5∓65.9% with RcTIP3, RcTIP4 and RcTIP5, respectively. RcTIP3;1 shares the similarity of 63.4% and 60.1% with RcTIP4;1 and RcTIP5;1, respectively, whereas RcTIP4;1 shares 57.3% sequence similarity with RcTIP5;1 (see S1 Table). Dual NPA motifs and three Froger’s positions (i.e. P3, P4 and P5) are highly conserved in RcTIPs (Table 2), in contrast, residue substitutions were observed at the P1 and P2 positions: the usual T is replaced by A in RcTIP5;1 at the P1 position, and the S is replaced by A in RcTIP3;1 and RcTIP5;1 at the P2 position. Of the ar/R filter, the usual H residue at H2 and I at H5 positions are replaced by N and V in RcTIP5;1, respectively; at the LE1 position, members of RcTIP1, RcTIP3, and RcTIP4 subgroups favor an A, whereas RcTIP2 and RcTIP5 members favor the G residue; residues at the LE2 position are more variable, including V, R or C (Table 2).

### RcNIP Subfamily

RcNIPs consist of 252–308 residues (Table 2). Except for the RcNIP4 subgroup that contains two members, each of the other six subgroups harbors a single one. The highest sequence similarity of 74.6% was observed between RcNIP5;1 and RcNIP6;1, followed by 61.7% and 60.0% between RcNIP1;1 with RcNIP4;2 and RcNIP3;1, respectively, and the lowest similarity of 33.8% was found between RcNIP4;1 and RcNIP7;1. Although both RcNIP4;1 and RcNIP4;2 harbor the same ar/R filter (W-V-A-R) (Table 2) and were clustered together (48.2% similarity) as shown in Fig 1, RcNIP4;2 exhibits higher sequence similarities of 42.5–61.7% with other RcNIP subgroup members than that of RcNIP4;1 (33.8–46.3%). The RcNIPs harbor typical dual NPA motifs except for RcNIP5;1 and RcNIP6;1 with NPS-NPV (Table 2 and S5 File). Except for the LE2 position, RcNIPs are highly variable in the ar/R filter and Froger’s positions in comparison to other subfamilies: W/A/G/T at the H2 position, V/I/A/S at the H5 position, A/G at the LE1 position, F/L/V/Y at the P1 position, A/P at the P3 position, S/T at the P2 position, Y/F at the P4 position and I/L/M at the P5 position (Table 2 and S5 File). In addition, one CDPK phosphorylation site corresponding to S262 in *Glycine max* NOD26 [46] was also found in the C-terminus of RcNIP1;1 and RcNIP4;1 (see S5 File).

### RcSIP Subfamily

There are only four members composing two subgroups in the RcSIP subfamily, which consist of 234–240 residues (Table 2). RcSIP2;1 shares sequence similarities of 39.4–41.0% with RcSIP1s. Within the RcSIP1 subgroup, the highest sequence similarity of 75.1% was observed between RcSIP1;2 and RcSIP1;3, and whereas the lowest similarity of 62.4% between RcSIP1;1 and RcSIP1;2 (see S5 File). In RcSIPs, the residues of the second NPA motif (NPA) and the P3, P4 and P5 Froger’s positions (A-Y-W) are highly conserved, whereas other positions are more variable, i.e., NPA/NPN/NPT of the first NPA motif, A/V/S-A/V/H-T/P/G-N/S at the ar/R filter and Q/E/I-A/V at the P1 and P2 Froger’s positions (Table 2).

### RcXIP Subfamily

RcXIPs vary from 208 to 309 residues in length (Table 2). With the exception of the RcXIP1 subgroup that contains four members, other two groups harbor only one. RcXIP2;1 shares 37.6–45.2% sequence similarity with RcXIP1s, which is considerably lower than that between RcXIP2;1 and RcXIP3;1 (76.2%), and slightly lower than that between RcXIP3;1 and RcXIP1s (39.5–47.1%) (see S2 Table). Among four RcXIP1s, the highest sequence similarity of 90.6% and the lowest of 42.8% were observed between RcXIP1;1 and RcXIP1;2, or RcXIP1;3 and RcXIP1;4, respectively. Compared with other RcXIPs, the length of RcXIP1;4 is relatively shorter (only 208 residues). Further sequence alignments indicated that RcXIP1;4 harbors only the first NPA motif and H2 and P1 positions (Table 2 and S5 File). In RcXIP1;1, RcXIP1;2, RcXIP1;3, RcXIP2;1 and RcXIP3;1, the second NPA motif and the LE2, P2, P4 and P5 positions are highly conserved, whereas other positions are variable, i.e., SPT/SPA/NPV/NPI in the first NPA motif, V/I at the H2 position, F/Y at the H5 position, V/A at the LE1 position, M/V at the P1 position and A/V at the P3 position (Table 2). In addition, like most XIPs [11,47], two highly conserved C residues in the LG/AGC motif of LC and the NPARC motif of LE were also found in RcXIPs except for RcXIP1;4 which is deficient in LE (see S5 File).

### Expression Profiles of RcAQP Genes

**Fig 3:**
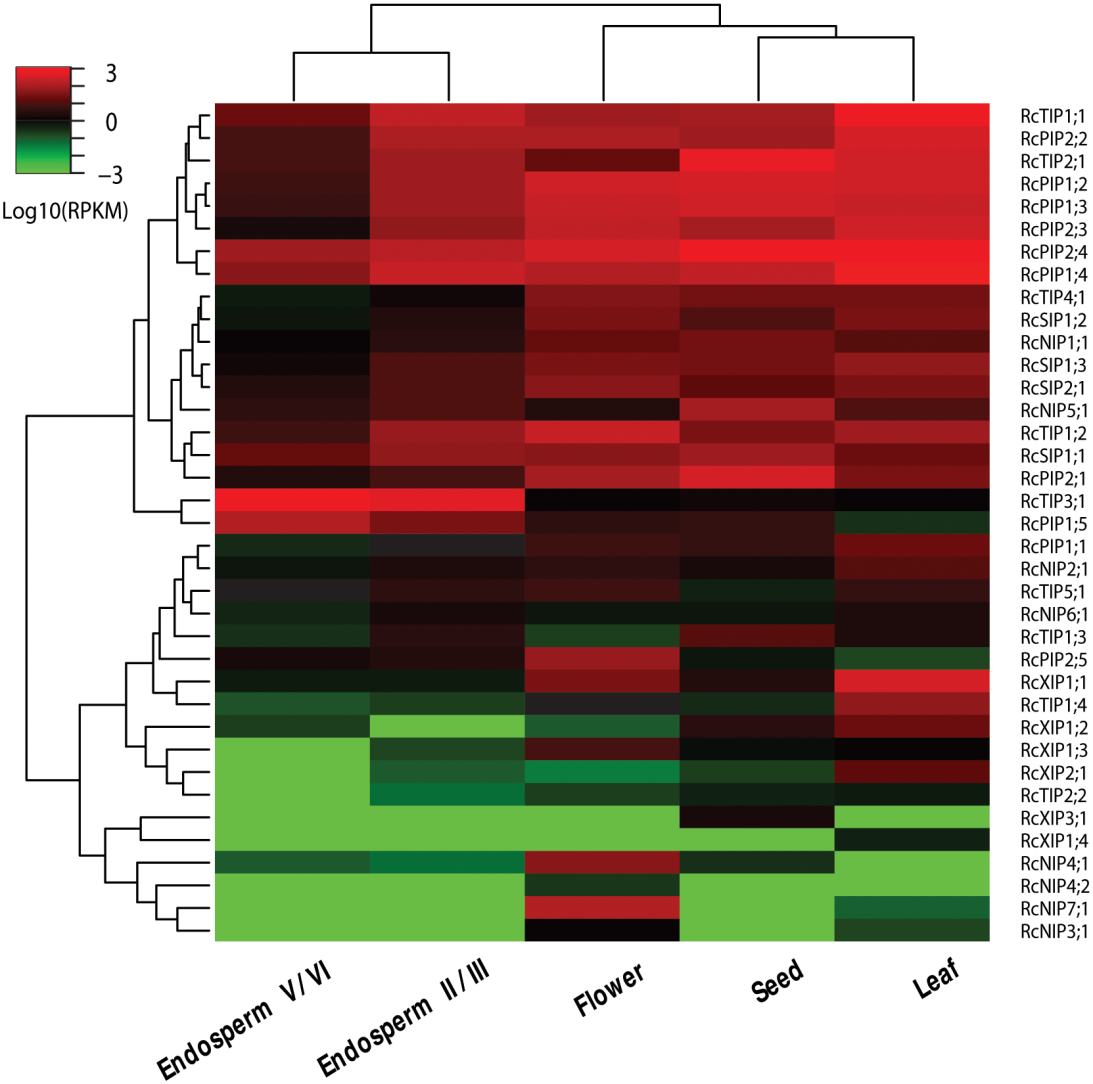
Expression profiles of the 37 RcAQP genes in leaf, flower, endosperm II/III, endosperm V/VI and seed. Color scale represents RPKM normalized log_10_ transformed counts where green indicates low expression and red indicates high expression.

To gain more information on the role of RcAQP genes in castor bean, RNA sequencing data of leaf, flower, endosperm (II/III, V/VI) and seed were investigated. Results showed that all 37 RcAQP genes were detected in at least one of the examined tissues, i.e., 35 in flower, 34 in leaf, 33 in seed, 31 in stage II/III endosperm and 29 in stage V/VI endosperm. According the RPKM annotation, the leaf tissue was shown to harbor the most transcripts, followed by the seed and flower tissues, and the endosperm has the fewest. Although the flower tissue harbors the most expressed AQP genes, its total expression level is only 0.46 and 0.71 folds of that of the leaf and seed tissues. In leaf, seed and flower tissues, the PIP subfamily contributes the major transcripts, in contrast, the TIP subfamily contributes the most in the endosperm. In the leaf tissue, six RcPIP members (in order as *RcPIP1;4*, *RcPIP2;4*, *RcPIP2;2*, *RcPIP1;2*, *RcPIP2;3* and *RcPIP1;3*) were shown to occupy about 98% of the total PIP transcripts, whereas *RcTIP1;1* and *RcTIP2;1* account for about 85% of the total TIP transcripts. In the seed tissue, five RcPIP members (in order as *RcPIP2;4*, *RcPIP1;2*, *RcPIP2;1*, *RcPIP1;3* and *RcPIP1;4*) occupy about 91% of the total PIP transcripts, whereas *RcTIP2;1* occupies more than 78% of the total TIP transcripts. In the flower tissue, six RcPIP members (in order as *RcPIP2;4*, *RcPIP1;2*, *RcPIP1;3*, *RcPIP2;3*, *RcPIP1;4* and *RcPIP2;2*) occupy about 89% of the total PIP transcripts, whereas *RcTIP1;2* occupies more than 63% of the total TIP transcripts. In the stage II/III endosperm, *RcPIP1;4*, *RcPIP2;4* and *RcPIP2;2* occupy about 67% of the total PIP transcripts, whereas *RcTIP3;1* and *RcTIP1;1* occupy about 82% of the total TIP transcripts. Compared with the other tissues and different developmental stage (i.e. endosperm II/III), the expression level of RcAQP genes is considerably low in the stage V/VI endosperm, nevertheless, *RcTIP3;1* occupies more than 95% of the total TIP transcripts or 69% of the total AQP transcripts. Although most genes were shown to be constitutively expressed in examined tissues, three genes seem to be tissue-specific, i.e., *RcNIP4;2* in flower, *RcXIP1;4* in leaf and *RcXIP3;1* in seed. Castor bean AQP isoforms seem to play different roles in various tissues. For example, *RcPIP2;4* is the most abundant transcript in both the flower and seed tissues, whereas *RcPIP1;4* and *RcTIP3;1* were shown to be expressed most in the leaf and endosperm tissues, respectively, suggesting their crucial roles in these tissues. In addition, the transcripts of *RcNIP5;1*, *RcNIP7;1* and *RcXIP1;1* were also shown to be relatively abundant in seed, flower and leaf, respectively (Fig 3). According to the cluster analysis shown in Fig 3, more similar expression pattern of RcAQP genes was observed between leaf and seed, and two stages of endosperm; two groups with distinct expression levels were also observed, where the more abundant group includes eight *PIPs* (i.e. *RcPIP2;2*, *RcPIP1;2*, *RcPIP1;3*, *RcPIP2;3*, *RcPIP2;4*, *RcPIP1;4*, *RcPIP2;1* and *RcPIP1;5*), five *TIPs* (i.e. *RcTIP1;1*, *RcTIP2;1*, *RcTIP4;1*, *RcTIP1;2* and *RcTIP3;1*), two *NIPs* (i.e. *RcNIP1;1* and *RcNIP5;1*), four *SIPs* (i.e. *RcSIP1;2*, *RcSIP1;3*, *RcSIP2;1* and *RcSIP1;1*).

## Discussion

### Small Number but High Diversity of Castor Bean AQP Genes

Compared with animals and microbes, AQPs are particularly abundant and diverse in land plants. To date, a high number of homologs have been identified from several plant species, i.e., 19 from *Selaginella moellendorffii* [18], 23 from *Physcomitrella patens* [9], 23 from *Vitis vinifera* [20], 33 from *Oryza sativa* [48], 35 from *A*. *thaliana* [16], 36 from *Zea mays* [49], 41 from *Solanum tuberosum* [14], 47 from *Solanum lycopersicum* [13], 55 from *P*. *trichocarpa* [19], 66 from *Glycine max* [15], and 71 from *Gossypium hirsutum* [12]. In contrast, the characterization of castor bean AQPs is still in its infancy. In the present study, a total of 37 AQP genes were identified from the castor bean through mining the genome and transcriptome datasets. Previously, one review also informed the presence of 37 RcAQP genes in castor bean, however, their result was merely dependent on the automatic genome annotation and only 34 out of the 37 RcAQP genes identified by this study were mentioned [50]. Although that paper focused on the analysis of TIPs, only seven RcTIP genes were described, whereas *RcTIP2;2* (30101.m000372) and *RcTIP5;1* (30147.m014231) were missed. Instead, three transcripts (i.e. 28962.m000435, 28962.m000436 and 30170.m014271) were misannotated as PIPs. In addition, four RcXIP1 genes were also misannotated as PIPs (S3 Table). The numbers of the castor bean AQP family are comparable to that of Arabidopsis and maize but less than that of potato, tomato, poplar, soybean and cotton.

**Fig 4:**
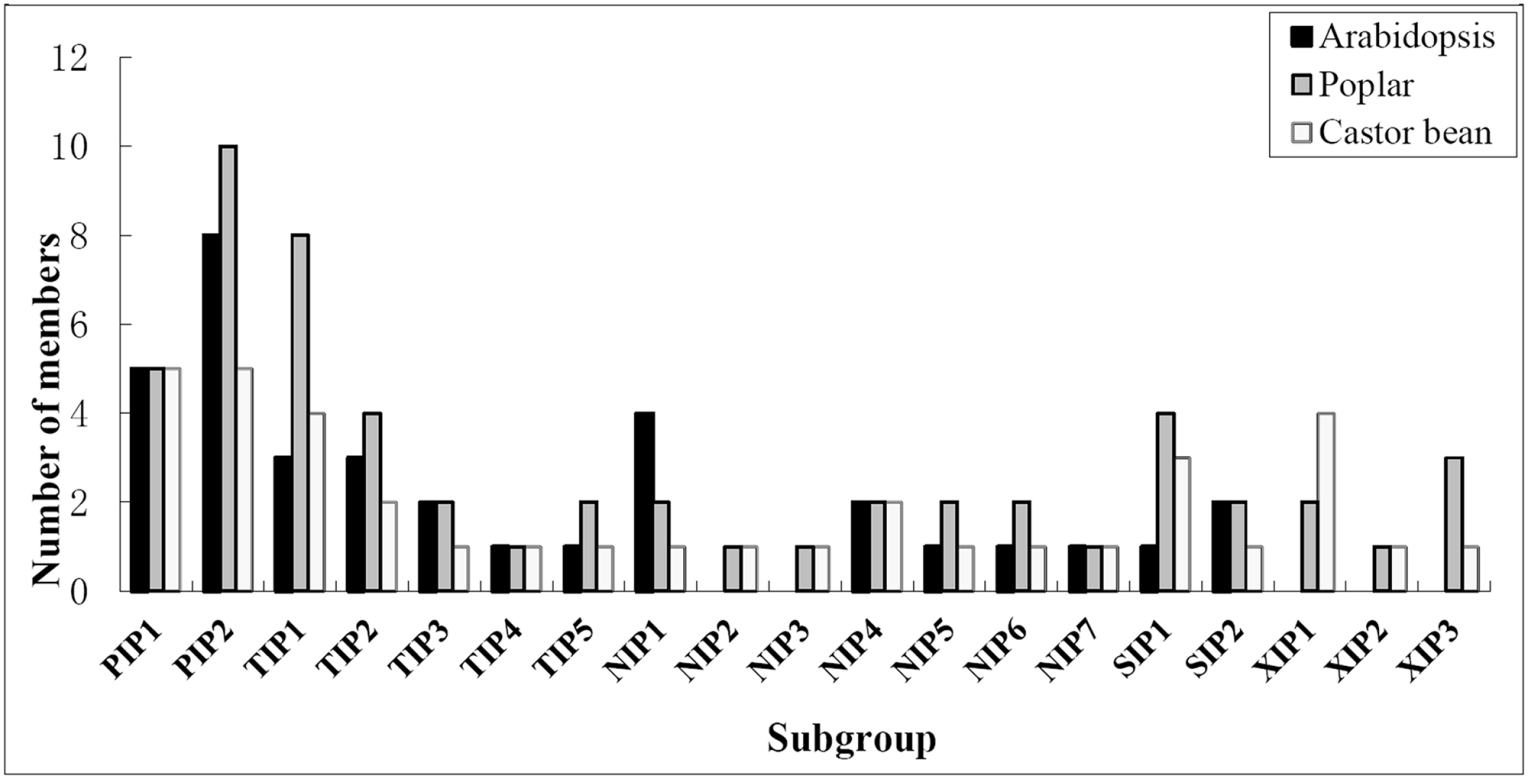
Distribution of the 37 RcAQP genes and their Arabidopsis and poplar homologs in subgroups.

Since the AQP genes in the model plant Arabidopsis and poplar were well characterized [16,19,47,51], their deduced proteins were added in the phylogenetic analysis of RcAQPs, which assigned the 37 RcAQPs to five subfamilies. Compared with Arabidopsis without XIPs, castor bean contains five XIPs and one more SIP but fewer members of PIPs, TIPs and NIPs. In contrast, the number of members in the five castor bean subfamilies was shown to be relatively smaller than that of poplar (Fig 4). With the exception of XIP subfamily, the further classification of RcAQP subfamilies into subgroups is consistent with Arabidopsis, i.e., 2 PIP subgroups, 5 TIP subgroups, 7 NIP subgroups and 2 SIP subgroups. However, it should be noticed that, as shown in Fig 1, the classification of AtNIP2;1 and AtNIP3;1 was not well resolved. In the case of AtNIP2;1, it shares the highest similarity of 67.0% with AtNIP1;2 in Arabidopsis, 64.7% with RcNIP1;1 in castor bean, or 62.0% with PtNIP1;2 in poplar. Given the same ar/R filter (W-V-A-R) and its closer cluster to the NIP1 subgroup, we recommend to class AtNIP2;1 to the NIP1 subgroup, thereby, no NIP2s were retained in Arabidopsis as seen in castor bean (RcNIP2;1) and poplar (PtNIP2;1) which harbor a G-S-G-R filter. In the case of AtNIP3;1, although it was clustered with the NIP4 subgroup, its closest homolog is AtNIP1;2 (61.7%) in Arabidopsis, RcNIP1;1 (61.6%) in castor bean, or PtNIP1;1 (60.3%) in poplar, thus AtNIP3;1 can also be nominated as an NIP1 member. In addition, according to the phylogenetic analysis and sequence similarity, we propose to rename PtNIP1;5, PtNIP1;3, PtNIP1;4, PtNIP3;3, PtNIP3;4, PtNIP3;1, PtNIP3;2 and PtNIP3;5 as PtNIP3;1, PtNIP4;1, PtNIP4;2, PtNIP5;1, PtNIP5;2, PtNIP6;1, PtNIP6;2 and PtNIP7;1 (see S1 Table). According to the nomenclature based on the ar/R filter that classes NIPs into subgroups NIP I, NIP II and NIP III [52,53], RcNIP1;1, RcNIP3;1 and two RcNIP4s can be also assigned to the subgroup NIP I, three members (i.e. RcNIP5;1, RcNIP6;1 and RcNIP7;1) to the subgroup NIP II, whereas RcNIP2 forms a new subgroup termed the subgroup NIP III as observed in rice and maize [53]. Since no XIP homolog was found in the Arabidopsis genome, the nomenclature proposed by Lopez et al. [19] for poplar was adopted to divide RcXIPs into three subgroups. Besides supported by high bootstrap values, XIP1s are characterized by the ar/R filter of V-F-V-R, XIP2s of I-F-V-R, and XIP3s of V-Y-A-R.

As seen in Arabidopsis and poplar, gene pairs were also observed in the castor bean AQP gene family, though the number is considerably small (Fig 1). For example, five AtPIP1s were clustered together apart from PIP1s of castor bean and poplar; RcPIP1;2, RcPIP1;3 and RcPIP1;4 were clustered with PtPIP1;3; RcPIP2;1 and RcPIP2;2 were clustered with PtPIP2;3 and PtPIP2;4; four RcXIP1s were clustered with two PtXIP1s; RcSIP1;2 and RcSIP1;3 were clustered with PtSIP1;3 and PtSIP1;4. It is well established that poplar underwent one whole-genome triplication event (designated γ) and one doubling event, whereas Arabidopsis underwent the same γ event and two independent doubling events [54–59]. And these duplication events mainly contribute to the gene expansion in these two plant species. Nevertheless, in contrast to poplar, the Arabidopsis genome encodes relatively fewer AQP genes due to massive gene loss and chromosomal rearrangement after genome duplications [54,60]. According to the comparative genomics analyses, the γ duplication occurred at approximate 117 million years ago, shortly before the origin of core eudicots [61]. As a core eudicot plant, castor bean was shown to share and only undergo the whole-genome γ duplication [25]. Since most gene pairs tend to be clustered in same scaffolds, tandem duplications are promised to be the main force for their expansion.

### Subcellular Localization and Functional Inference of RcAQPs

In comparison to non-plants, plant AQPs exhibit a broader subcellular localization, including plasma membrane, vacuolar, endoplasmic reticulum (ER), Golgi apparatus, mitochondrion and chloroplast, etc., corresponding to the high degree of compartmentalization of plant cells [3,62]. Our subcellular localization prediction of RcAQPs included the plasma membrane, vacuole, chloroplast, peroxisome and cytosol. As observed in other plants and suggested by their names [62], basic RcPIPs and acidic RcTIPs are localized to plasma membranes and vacuoles, respectively. All RcNIPs were predicted to target the plasma membrane, though their homologs in other organisms were determined to localize to the plasma membrane, ER or peribacteroid membrane of root nodules [63–65]. Compared with the diverse localizations predicted by WoLF PSORT, all XIPs were predicted to localize the plasma membrane by Plant-mPLoc, which is consistent with experimental results [66]. RcSIP2;1 was predicted to target the ER as reported in Arabdopsis and grapevine [67,68], in contrast, three RcSIP1s were predicted to localize the plasma membrane, vacuolar and chloroplast. Thereby, further investigations are required on the subcellular localization of RcAQPs.

Although plant AQPs were first known for their high water permeability, when expressed in *Xenopus* oocytes or yeast cells, increasing evidence has shown that some of them are also participated in the transport of other small molecules such as glycerol, urea, boric acid, silicic acid, NH_3_, CO_2_ and H_2_O_2_ [2]. As shown in Table 3, most RcAQPs exhibit an AqpZ-like Froger’s positions to favor the permeability of water. In contrast, NIP subfamily members possess mixed key residues of GlpF for P1 and P5, and AqpZ for P2–P4. Given the glycerol permease activity of soybean NOD26 and Arabidopsis NIPs [69,70], RcNIPs are promised to transport glycerol and may play roles in oil formation/translocation.

In addition to high permeability to water, plant PIP subfamily members were reported to transport urea, boric acid, CO_2_ and H_2_O_2_ [71–78]. As shown in Table 3, all RcPIPs represent the F-H-T-R ar/R filter as observed in AqpZ which harbors an extremely narrow and hydrophilic pore (diameter 2.8 Å) [72], suggesting their high water permeability. Based on the SDP analysis proposed by Hove and Bhave [10], all RcPIPs represent urea-type SDPs; RcPIP1s represent boric acid-type SDPs; all RcPIPs represent H_2_O_2_-type SDPs, supporting their similar functionality. In addition, RcPIP1;1, RcPIP1;3, RcPIP1;4, RcPIP1;5, RcPIP2;2 and RcPIP2;4 seem to represent novel CO_2_-type SDPs (I/L/V-M-C-A-I/V-D-W-D-W) with the substitution of I for M at SDP2.

Although highly variable in the ar/R filter, plant TIPs were shown to transport water as efficiently as PIPs [79]. Additionally, they also allow urea, NH_3_ and H_2_O_2_ through [80,81]. As shown in Table 3, all RcTIPs represent urea-type SDPs, whereas RcTIP5;1 represents H_2_O_2_-type SDPs, indicating similar functionality. Compared with typical NH_3_ SDPs, RcTIP2;2 seems to represent novel SDPs (T-K-T-V-A-S-A-P-S) with the substitution of S for A/R/T at SDP9.

As well as glycerol and water, plant NIPs have been found to transport urea, boric acid, silicic acid, NH_3_ and H_2_O_2_ [63–65,80–82]. As shown in Table 3, RcNIP5;1 is promised to be a transporter of urea, boric acid and H_2_O_2_; RcNIP1;1 is promised to be a urea and NH_3_ transporter; RcNIP3;1 and RcNIP4;2 are potential urea and H_2_O_2_ transporters; RcNIP4;1 and RcNIP7;1 are potential urea transporters; RcNIP2;1 is a potential urea and H_2_O_2_ transporter. In addition, compared with typical silicic acid SDPs, HbNIP2;1 seems to represent novel SDPs-types with the substitution of V for A/E/L at SDP3 or Q for A/K/P/T at SDP9, which is similar to that of GmNIP2;1 and GmNIP2;2 (S-Y-E-R-G-N-R-T-P) [83]. RcNIP6;1 may also be a urea transporter representing novel SDPs (H-P-I-A-L-E-G-S-N) with the substitution of E for A/G/P at SDP6.

As a recently identified AQP subfamily, plant XIPs were shown to transport water, glycerol, urea, boric acid and H_2_O_2_ [17,53]. According to phylogenetic relationships, XIPs are split into two independent clusters termed XIP-A and XIP-B, where XIP-A includes only XIP1 subgroup and XIP-B contains at least four subgroups, i.e., XIP2, XIP3, XIP4, and XIP5 [17]. Consistent with poplar XIPs (2 XIP1s, 1 XIP2 and 3 XIP3s), six RcXIPs can be assigned to subgroup XIP1 (4), XIP2 (1) and XIP3 (1) (Fig 4). When expressed in *Xenopus* oocytes, PtXIP2;1 and PtXIP3;3 were shown to transported water while other PtXIPs do not. Although the mechanism why PtXIP1s, PtXIP3;1 and PtXIP3;2 can’t transport water is still unclear, the close homologs of PtXIP1s in tobacco (*Nicotiana tabacum*) and potato were also reported to have undetectable water permeability. In contrast, Solanaceae XIPs showed high permeability to glycerol [66]. Therefore, although exhibiting an AqpZ-like Froger’s positions, all RcXIPs are promised to transport glycerol. Meanwhile, RcXIP2;1 and RcXIP3;1 are probably capable of transporting water. As shown in Table 3, RcXIP1;1, RcXIP1;2 and RcXIP1;3 may be H_2_O_2_ transporters representing novel SDPs with the substitution of S for I/L/V at SDP5.

### Distinct Expression Profiles of RcAQP Isoforms in Various Tissues

Water is essential for all life on earth. Like other organisms, plant growth and development depends on water uptake and transport across cellular membranes and tissues. Thereby, water stress forms a major factor that decreases plant growth and productivity. One important response of plant cells to water stress is the regulation of AQPs [84–87]. Although plant AQPs were reported to be regulated by posttranslational modifications (e.g. phosphorylation, methylation and glycosylation), gating, heteromerization and cellular trafficking [88,89], the transcriptional regulation still acts as the key mechanism. Since the organ specificity of AQP expressions may be closely related to the physiological function of each organ, we took advantage of deep transcriptome sequencing (also known as RNA-Seq, an approach to transcriptome profiling through sequencing the total cDNA) data to survey the expression profiles of RcAQP genes from a global view. Results showed that the transcripts of RcPIP and RcTIP subfamily members are highly abundant in all examined tissues, which is consistent with that observed in other plant species such as maize, Arabidopsis, tomato and potato [13,14,49,51]. Considering PIPs and TIPs are highly permeable to water [79,90], their high abundance indicates their crucial roles in intracellular, cellular, organic and whole plant water balance of castor bean. *RcPIP1;4*, *RcPIP2;4* and *RcTIP1;1* were considerably abundant in leaves and are promised to play the key role in leaf hydraulics. *RcPIP2;4*, *RcPIP1;2* and *RcTIP1;2* are more likely to control the flower water balance for their high abundance in this tissue. The highly abundant *RcPIP2;4* and *RcTIP2;1* are promised to govern the seed water balance and *RcTIP3;1* is promised to monitor the water balance of endosperms (regardless of stage II/III or V/VI). Compared with other tissues or developmental stage, *RcTIP1;1* is expressed more in leaf and endosperm II/III, in contrast, its closest homolog in Arabidopsis (*AtTIP1;1*, encoding a transporter of water, urea and H_2_O_2_) was reported to be highly expressed in vascular tissues of root, stem, cauline leaf and flower but not in the apical meristem [79,91–95]. Despite without a strict organ-specific expression pattern, *RcTIP1;2* is preferentially expressed in flowers, by contrast, its closest Arabidopsis homolog (*AtTIP1;3*, encoding a transporter of water and urea) was shown to be expressed highest in mature pollen, moderate in flower, very low in inflorescence and non-detectable in any other tissue [93,96]. Although the castor bean genome encodes two TIP2s, these two genes exhibit distinct expression profiles. Despite very low, the transcripts of *RcTIP2;2* was observed in leaf, seed, flower and endosperm II/III, but not detectable in endosperm V/VI. In contrast, *RcTIP2;1* is expressed in all examined tissues and the transcript level is considerably high in seed and leaf. In Arabidopsis, the closest homolog of *RcTIP2;1* is *AtTIP2;1*, encoding a transporter of water, urea and NH_3_, which was shown to be mainly expressed in flower, shoot, and stem, and to a lower extent in roots [93,97–99]. *AtTIP3;1*, an orthology of *RcTIP3;1* was reported to be a seed- and embryo-specific AQP gene [100], in contrast, *RcTIP3;1* was preferentially expressed in the endosperm of developing seeds and considerably low in germinating seed. In addition, it is noteworthy that three putative non-aqua transporter encoding genes (i.e. *RcNIP5;1*, *RcNIP7;1* and *RcXIP1;1*) were shown to be highly abundant in certain tissues. Compared with other tissues tested, the transcript level of *RcNIP5;1* is considerably high in seed. Like castor bean, the Arabidopsis genome encodes a unique NIP5 member (AtNIP5;1), which was shown to transport boric acid and arsenite as well as water [65,101]. Expression analyses indicated that *AtNIP5;1* is mainly expressed in root epidermal, cortical, and endodermal cells and the transcript is upregulated in response to B deprivation [65]. *RcNIP7;1* can be regard as a flower-specific gene, because its expression level in leaf is extremely low. Similar to castor bean, *AtNIP7;1*, the orthology of *RcNIP7;1* in Arabidopsis was also found to be specifically expressed in anther, encoding a less efficient boric acid transporter in comparison to AtNIP5;1 and AtNIP6;1 [102]. *RcXIP1;1*, a less characterized AQP subfamily member not found in Arabidopsis [19,47,66], was considerably abundant in leaf tissue, thus further investigating its function is of special interest. These results indicated that distinct AQP evolution occurred after the divergence of castor bean from Arabidopsis, and the AQP functional characterization in specific biological process needs to be performed beyond model plant species such as Arabidopsis.

## Conclusions

To our knowledge, this is the first genome-wide study of the castor bean AQP gene family and using systematic nomenclature assigned 37 RcAQPs into five subfamilies based on the sequence similarity and phylogenetic relationship with their Arabidopsis and poplar counterparts. Furthermore, their structural and functional properties were investigated using bioinformatics tools. The global expression profiles of these 37 RcAQP genes were examined with deep transcriptome sequencing. And putative transporters of water, glycerol, urea, boric acid, silicic acid, NH_3_, CO_2_ and H_2_O_2_ were also predicted and discussed. The RcAQP genes identified in this study represent an important resource for future functional analysis and utilization.

## Supporting Information

S1 File The gene model for *RcXIP2;1* and *RcXIP3;1*. (PDF)

S2 File The gene model for *RcPIP2;5*. (PDF)

S3 File The gene model for *RcTIP2;2*. (PDF)

S4 File The gene model for *RcNIP4;1*. (PDF)

S5 File Alignment of deduced amino acid sequences of castor bean AQPs with structure determined spinach PIP2;1. (PDF)

S6 File SDP analysis of castor bean AQPs from alignments with amino acid sequences of AQPs transporting non-aqua substrates. (PDF)

S1 Table List of the Phytozome accession numbers of the AQPs genes identified in Arabidopsis (35) and poplar (55). (XLSX)

S2 Table Percent similarity within and between five subfamilies of the RcAQPs. (XLSX)

S3 Table Comparison of the 37 RcAQP genes identified in this study with previous annotations. (XLSX)
