# Supplementary material for: Gene Structures, Evolution, Classification and Expression Profiles of the Aquaporin Gene Family in Castor Bean (Ricinus communis L.)
Source: PLoS One. 2015 Oct 28;10(10):e0141022. doi: 10.1371/journal.pone.0141022 (PMC4625025; doi:10.1371/journal.pone.0141022)
Supplement: S1 File — (PDF) [file pone.0141022.s001.pdf]

S1 File. The gene model for *RcXIP2;1* and *RcXIP3;1*. A, The coding region is marked with uppercase letters (the intergenic spacer between *RcXIP2;1* and *RcXIP3;1* is also shown in uppercase letters but labeled with single underlines), under which is its deduced amino acids. The introns are marked with lowercase letters. The start and stop codons are blacked. **B**, Read-mapping graphical representation of *RcXIP2;1*. **C**, Read-mapping graphical representation of *RcXIP3;1*.

(A)

```
    1 tgaagggcacaatacagtatggtttgcacttaacgaagatctaatcatcttgacattgtt
   61 ggcttcagtgatgctgattggacctttgatcccaacgataggaggtctacttctggtttc
  121 tgtatttttctagggagcaaccatgtttcttggaactgcaagaagcaatctactgtatca
  181 aggtccagtatagaggctgagtatagaagctgggctaatctagttgctgagttattttgg
  241 ataaactctcttctgtctgaacattaagtttctgttcctcgagctcctgttgtgtggttt
  301 gataatcagagtacaatgcttctctcagctaatgccgtgttacatgcaagatcgaagcac
  361 attgaactcgatttgtattttgttaaggaaaagtggttaacaaagaaattgatttaaggt
  421 atgcctcgtctgttgatcaattggcagatgtgtttactaaggctatttctagttcagtct
  481 ttaattcctctagattcaaacttaatgtagaaaatctttctatcctaagtttgaggaggg
  541 atgttaagaatggttaatgtgtttgttacgtgtttgttactgagctggtatatagtttat
  601 tcgagagggacacgtgtcattataagaatggttcggatgatttttagttggagtatttat
  661 gttaatatttcattgtattgttgagtaacacaagattaataatatacaaacaagattctc
  721 tcatttcacattaagtgctagtaattattcctcaactgccaacctatcctatcattatat
  781 taagaccttattttatatgatggaaaatagccaaagttgaaccaagaaaatttcataga
  841 atcATGGCGGATAATCTAAGGGTTATTGCTGATGAGGAAAATGGCTACGGAGGAAGAAGA
    1     M  A  D  N  L  R  V  I  A  D  E  E  N  G  Y  G  G  R  R
  901 GTTCAACCTTTTGCCTCTACACCACTAGGgcaagtagtctcaaattttctacagtcgtct
   20 V  Q  P  F  A  S  T  P  L  G
  961 ttcttttagattttgtgggttttgttgcatatgaactgagtatacttggttctgattttg
 1021 cagGGCAGCTCTGGACAATACTAACGGTGGAAAGAAGCAAAATCCTACTACATTCAGTAG
   30     A  A  L  D  N  T  N  G  G  K  K  Q  N  P  T  T  F  S  R
 1081 GGTTTTGGGCTTGGAAGAGCTTTCTTCTTTAAATgttagtaatatatctcttacgagcag
   49  V  L  G  L  E  E  L  S  S  L  N
 1141 tttacgtagttatagctaccgacagggtcaagcctgaaaactattttctttagcaaaatg
 1201 acaatatcttgcccaaaatgagtaaaattaacatagaaagtaagcactaactgtggcctt
 1261 gactagctagtagcttctgtcttctcttcccacccactaactactaatgtatgtcgatta
 1321 ttgaatttggcaacgccacctttagtacaattgaattacatgtaattgatgggcggatta
 1381 caattttattactatcatcagGTATGGAGAGCATCTGTGGCCGAGGTTCTAGGCACAGCA
   60                       V  W  R  A  S  V  A  E  V  L  G  T  A
 1441 GCCCTTGTCTTTGCTACAGACACTATAGTCATCTCCACCTACGAGACCGAAACAAAAACA
   73 A  L  V  F  A  T  D  T  I  V  I  S  T  Y  E  T  E  T  K  T
 1501 CCAAACCTTATAATGGCAGCTTTAATCGCCATGACAGTCACAATTCTTCTCACTGCCACA
   93 P  N  L  I  M  A  A  L  I  A  M  T  V  T  I  L  L  T  A  T
 1561 TTTCCCATCTCTGGTGGCCATATCAACCCTGTCATCACCATTTCTGCTGCATTTACAGGC
```

113 F P I S G G H I N P V I T I S A A F T G

1621 CTTGTTTCTCCTGTACGGGCTGCTGTATACATCTTGGCCCAATGTCTAGGAGCCACACTA

133 L V S P V R A A V Y I L A Q C L G A T L

1681 GGCGCACTAGCACTAAAAGCTGTGGTAAACAGCAGAATAGAGGAAACATTTTCTCTTGGA

153 G A L A L K A V V N S R I E E T F S L G

1741 GGGTGCACTCTAAACATTGTTGCACCAGGCCCACAAGGACCCATCGTGATCGGCCTTGAG

173 G C T L N I V A P G P Q G P I V I G L E

1801 ACGAGTCAAGCTCTCTGGCTGGAGATAATCTGTACATTTTTGTTCCTATTTTCTTCAATC

193 T S Q A L W L E I I C T F L F L F S S I

1861 TGGCTGGCATTTGACAAGCGTCAGGCCAAACTTTTAGGTCAAGTCATCGTTTGCAGTATA

213 W L A F D K R Q A K L L G Q V I V C S I

1921 ATTGGGCTGGTAGTGGGCCTGATTGTGTTCATATCGACGACGGTGACAGCCACGAAAGGA

233 I G L V V G L I V F I S T T V T A T K G

1981 TACGCAGGGGTCGGGATGAACCCGGCTAGGTGTCTAGGCCCAGCTCTTGTTAGAGGTGGC

253 Y A G V G M N P A R C L G P A L V R G G

2041 CACCTATGGAATGGGCATTGGGTATTTTGGGCTGGGCCTGTGATTTCTTGTGTAGCTTTT

273 H L W N G H W V F W A G P V I S C V A F

2101 GCTATTTACACTAAGATAATTCCAAAAGCTGAGGTCCATGCA**TAA**gggtgataactgatt

293 A I Y T K I I P K A E V H A *

2161 tacatccatatttgaatgtggcatttaaaatggtctcaacaagtaagaatgcatctttat

2221 gctgctttttttatgttttccagttagaggcttttactgttggggcatttgcactgcacca

2281 aacttattaatgtctgttaactttaaaattgtgtttttgtgatcctggcacaatgatgct

2341 aaaatgtaatatatctcagtttattatcaggacttctggcttttcttttgttatattgtc

2401 aatttatatagcagcatatatgtcatttctccttgatgataactagatttaataaagcaa

2461 aAAACAGTGCTAGTCCTTACGTTGGAGTCTTAGTTGCTAAATATAGTATGAATTATATAT

2521 ATATATATATATATGATTGACCAACGAAATAATATCATGTAATGTTTATTTATTTAGTTG

2581 GTATAAAGAATTGTGGAAAAGAAAGTTAATTTTCATGATTTCAAATAATACTTTTAAAAA

2641 AAATTAGGAAAGAGAATTACAATTGACGAGATTAAAATATAATATCTGCTCTTAAATAGT

2701 ATTATAATTCAGAGCTGAACCAAGTTATCATATTATGTCAAATTTCAATCCAAAATCAAT

2761 TGATATTGATACTATGTTTGGTTAAAATTCACTAAGAACTTCATTTTATTTTAAAAAATA

2821 TAAGAGATAATATAAAATGAAAATAATAAAGATATTTTAATGTATGAAATATATTAATTT

2881 AAAAATTTAGTTATAACTAATTTTACATAAATTTAATTATATAAAATTTTAAATAATTTT

2941 CACTTTATTTAAACCATTTAAATCTTAAATGCANNNNNNATTGATCTATAATTAACAAAT

3001 TATATTCTACAGCTAAATCTCCACGTAAGAAATGGGAGCTGATCCATGAATCTATGATTA

3061 AAAATAATAATAATTTTTTATTTTGTTACACAATATAAAATTAAATTTATAAATAAAAAT

3121 TAATAAATTTTTTAATATTTAATATTTATTTATGATTTTTAAAAATTAATAGTAAATTAA

3181 ATAGTTTAGATGTCATTTTTAATTTTTTAATATTTTAAAATTTTATTTATGCATTAATAT

3241 AATAATTATTTTAAAAACACTTATTAATTAATTTTAATATTAAATAAAATAATTCTATCA

3301 ACATAATTGATATAACTCTTTTAAGGATTACAAATTATTATATAACTAAAAATTAATTTA

3361 CTAATAATATAATATCTAAAACATAATTTAAAATATTATTTTTTTTAAATTAGGATTATT

3421 ATCTAATTAAAAATAAATTTAATATAATATTATAATATAGTTAATATGTTAATTTTTACA

3481 ATAGAATTAAAATTATTATCTAATAAGAAAATTAAGTTATTATTTAATATAATATTAAAA

3541 TATAATTAAACTGTTATTTTTAGAATTAGAATCACTATTTAATTAGAGATGTGACTTATG

3601 AGTCAATAAATAGAAAAAGAAATATTAAATTATACATAAATTTGAATTCTATATGTCAGA

3661 AATAAGTACACATGTCAAAAAAATAATTTAATATATATATATATATATATATATAATTAA
3721 ACTTATTTATTTATTTGTTAGGGTATTCTCAAACCAAGACGGAATTGATGAATGATTCAA
3781 CTCAAGATATTTATCAAACAATAGAGGAACATATCCAAGATTTGATATCTTTGGAGTCAC
3841 AACCTTATCTTCATCACTGCTCTCACTTCAAAAATCATAAGTAATATAAGTTTTATTATA
3901 GGAATATTGATGTTAGCAGCTATGATAGTTAGCCCCTATGTTAAAACTCATTTTATAATC
3961 TTTGCATTTTGCTTTCTAATTTATATCTTAAGTAAACAACAATGCAAAAACAACCCATGT
4021 CCTGAAATCTTCTTAGCTGTACGTAAGATATTTGTCTAGTCGTTATCTACAAAAGAATGA
4081 ACGAACCTCTTTTTAGTATATTTGATGGATATACTTCATATTCTCACCTTGCATTTTGTC
4141 CTTTAGACCAAACCATTTTTATTACAAAACCTAAGAATGTTAATGTAGTTTTGCATTTGG
4201 AGTTTTGGAATCTTCGTAAATGAAATTGACCGATCGGCAAAATCCTTATATATTGATGAA
4261 TAATTATCTCCTATATGCTAAGAGAAAAGGACTAATTACTTATTCTCGCAAATATATAGA
4321 ATTTAAGGCATGGATTTGAATGTAGTCTCACAAGATGCTTGTCATCAATCACCATTCTCA
4381 AAGTCACTTGAAAATGGCCACACAATCAGTAACTTCACAGCCACAAAAGAATCTTTGAAA
4441 ACAGGCTTTCTTGATCATATTGGTGCTCATGAATTCTTTTCAACAGAGGTAATTAATGTG
4501 AATTGATATTATTTATCTTTGAAATCCATCTTTGAATGATTTCATATTTATCTTCCAGAT
4561 GTGGAGAGCAACATTCACTGAGCTCGTAGCAACATCTTGCCTCTTATTCACACTGACCAT
4621 ATCTATCATCTCAAGCTTAGAATCACATGAATCAGAACCCAAGATTCTAGTCCCATTGGC
4681 AGTCTTTATAATTGCCTTCTTCTTCCTCTTAACAACCGTTCCTTTATCTGGAGGTCATAT
4741 GAATCCAATTTTCACCACCATTGCTGCCCTTGAAGGTGTTATAACTTTCCTTCGCGCCAT
4801 ATTCTATATCCTGGCACAATGTGTAGGCTCAATCCTATCTTATATGATAATCAAGAGGGT
4861 GATGAACAAAAATGTAGCTAAAAAGTATGCTTTAGGTGGCTGCATGATCAATGATGGAAA
4921 TGGAGAAGGGGTAACCCTAGCAACAGCATTAGTGCTTGAGTTTTCTTGCACTTTTTTGGT
4981 CCTATTTGTTGGTGTAACAGTAGCATTTGACAAGAGAAGGTTTAAAGAGCTTGGATTAGT
5041 AATGGTATGTGCAGTATTGGCAGGAACCATGGGATTAGCTGTTTTTGTTTCAATTACTGT
5101 AACTGGAAAGAGTGGCTATGGTGGTGTGGGCTTGAATCCTGCAAGATGTTTAGGGTCAGC
5161 TTTATTGCATGGAGGTGCATTGTGGTATGGGCATTGGGTTTTCTGGGTTGGACCATTTTT
5221 GGCTTGCATTGCTTATTACTGTTATACTTTGACCTTACCAAAGGAAGGCATGGGAAATAT
5281 AGATGCTGTAGATTGATAAATCATTAAAGTACTTTAAATTTAGTGTTTTTCTTCTTTGTA
5341 TTGAATGAAAATTTTATTCTTTTATTAGAAACTAGAGGTATACCTATCCTATGGGCGGAA
5401 TATCATTTTATTTATATAATAAATAAAAATAAAAATATATTAGACATTAATTAAAATTTA
5461 AAAGATGAAATTATGGTATAAAATTATTTTTATTTTAAATGAGAAAATAATATAATTTTT
5521 TTTTGCATAATCATTTTAAAGCGCCGAATAAATATAAATGTCAACTAATTTTAATCAAAG
5581 GAGGAAACAATTATAAATGAGTAGATAAAATATGATTTGGAATACTAAAATTATAATAAA
5641 ATACGATTAGTATGTATTAATTTAATAAATTAAACTTGTTATAAAATAAATAGATATAAT
5701 ATAATTTGACTACTGAAACTACAATAGAGTATGATTACTAAGTGTTAATTTAATAAATTA
5761 AACTTGTAAGAGTAATCCTTATTATTTTACAGAATTTATATCAACACTTAGTCACTCTCA
5821 CTTTTAATAGTTACTCGCAATTATTGATAATAGAAATCAAAAACTAATATTTATGAAAAA
5881 AATATGATAAGAACAACTATATAATATTTTCTTCATATTTTTAATTCTTTTGCATAATAT
5941 GCATTGTCAAACTTGTGAATGAAGTAAATGAATTATAGTGTTGGGGAAGAAAATTAAAAC
6001 ATCAATATACAAAAGATATTTAATAAATCTTTAATTCAAGTCACATTTCTATCTATTGCC
6061 TTAACATTGTCCCAAATACAAAATAATGCTTTCTTCATATGAAAATTCTTACAAAAAAAA
6121 AAACTCTTGTTAGCATTTGTTTCGAGCAAAATCATTTATATATATATGAATACGAATGAA
6181 ATGAAATTTATCTTCTCAGCCAATTCAATCTTAAAATATGATTAGCATATAATGAATATC
6241 CAATATATAATATAATGTTAATTCTCAAGACTTCTAATTATAATAATCTACTCCCATTAA

6301 AATATTTTCTATATGATGAATTTTATAGTTTTATAAAGAAAACAAAAGGAAATAAATTGT
6361 TACGAGTAAATTTTTATGAATATTTACATTAATTCATAATAGGATACATGTGAATAAAAT
6421 ATCAACATGAAATAAATAAATTATAATATATGAATTATGCTTGGCTAGAAGCAATAACAA
6481 CAAAGAAGTTGTAAACTTTTTGACCATAAGCTTATTTTGATAACACAATCCTTACCAATA
6541 ATACATACTATTTGTAACATCAGCATTAAGCATCTTAGATGGAGACAAATAGTGAAATTG
6601 ATTATAAATTGGAGATGAATAGTTTAGAAGCTGCGAAAGAGAGTTCAAAATTCTCAATTA
6661 TATATATATGTTTGCAAATCACATATTATTGGAAAGATCTAACTCTATAATTTTTTTTTT
6721 CAACAAATTCAATGTTGCATCATTCTTTATCATTATTGAGCAAACCAAAGGCAATCACAT
6781 GTTTTTATCATTGTTAAAGTAACAACAAGCATATTTGTATTTATTTTTCTATGTGTCAAA
6841 TAAACCTTTCATAATCTATATGGTACAAATGAATTTTGATATATTATAGAATTAAATGCT
6901 AATTGGGAAAAAGAAAAAATTAGATGTATTTTAATTTATAGGTTATAAATTTTTTCAATT
6961 TATTGATTATGTCTTTTATAGAAATACTTATTGTTTATTCTCTTACGTCAACCCAGTTTT
7021 TCTTTTTAAAAAAAGTTTAAAATTCTAAAAATAATCTCATTTCATCATTATTAAAGCATT
7081 TAAAAGCTATATTGAATTGAAATAGACTATTAAACTTTTTATTATTAATAAATTGAGGTT
7141 ATATATATAAAAATTTGTAACTTACCAATTTGATTTAAATAATTGAATATTTAATTGAAA
7201 AATAAATTATAACTAATGATGTTTAATTTATTTATTTAAATATCTTTTATAATAAGATAG
7261 CTTATCAAGATGAAAGAAAATATTTCAAAATCCATAAAAACATTAAAAGAGAAAAAAGAG
7321 TATTAAAAGAACAAAAAGAAAAAGCTAAAATTAAGAGAAAAATAGTTTAAAAAAAAATAT
7381 AGAATGCCATCTAAAAGAATTTATATAAAAATATAATTTTCATGTATATAGATTTAACTT
7441 TTTCTAGATCGGTAATGAAAAGCAAGAAGCCAAGTTGACTTACTTAACATTGAGGGATAA
7501 TTGCCATTAATGAATACTTAATATTGTTTCTGATGATTTAGATGAAAACGAGTTAAATAA
7561 GCTTCAACATAATTTATAATTGCCATTAATGAATTATATAATTATCATATGTATATTTTA
7621 TTTGGTGATGACTTGATTTTCACTTTATTTCTACCGTCCATACCATGTGAGACCCTACTT
7681 CAATGTTCTATAATGGTTATTGAGAAAGAATTTGTTTTGTTAAAAAACAAAACAAAAAAT
7741 TATCATAGAATTCCTGCTTTTTAGGAGTTCTTAGGGCAGAAAACGACCTCTCCCATGAAA
7801 GATTGTTTTATAAGGATTGTTGAGTTTGCATGTGCAATCTTCACATGCATTTATTATATT
7861 TTATTAAAAAAGACATATATTTACTGTCCTACTGGCATCGATGGCTTTTCTTTGCTGTCG
7921 ATAATAGCTATATTTGATTTTTTACTTTCTGTTTTTTTTTTTTTTGTAATTTTTTTAGTC
7981 ATAATAAAAGTAACCAAATTATATTTCTATTTTATTTATACAGTTGGTCAGAATTTATAA
8041 ATGTTGATTATAAAATATTTTACTAATGAATTTTACAAAATAGATGATAATGAAAGAAGA
8101 TCCTATGGCTATTAAAGTTTTAATATTCGGAGATAAATCAAAATTTATCATTAATAAATA
8161 TGAATTTATTTAGTTGAAGGTTTGTTTAAGATTTTATTTTATATTTATTTTTTAATTATT
8221 TATTAATTTTATTGATTAAATATTATAGATATTTTGTTTAGTTTATTTTTTACGGAGGTT
8281 TGTCTTTTTTATAGAATTTTTTTTTATGAGAGTTATAATGAGTTTAATTATAATATTTAA
8341 AATGATAATGAAATCGATCTGTGGCTTAATATAGCACCAAGTGGCATATATTTCATATCA
8401 ATATGCCAAATGCAGCCAATATACTGTATATAATTATTATGTCAATACGATAAATGAATA
8461 TTAGAAAAAAATTTCATTTTTATATCAAGATTAATATTTATTTTTTGAATGAAATTTACT
8521 TATATTTATTAATCATTTTTAATGAAATATTCTTTTTATTTTTTTTTTAACGAGATACTC
8581 ATATATAATACCTACAAAACATTATCCAATTGAAGGATCCCTGCTATTGCTTTAACAACA
8641 ATTCCTTGGGTACACTATGTCCTGGCAAATGAGGTGTTATGGGATCAAAGTGCACCCTGA
8701 CAGGAAAAATGAAAAGCATTAAAAATGATGATATCCCTTTATTTATGATCCCATAAACAA
8761 AGGGATATGGTATCTCAGCTGATATATTTGCAATTTTCTTTTTCATGTGAACTGTGCACG
8821 AACGCAAGACAAAATTGGAAAGATATTATGAACTTGGTCTAGATATTTATCATGGCCAAT
8881 TTTTAAAAACTAAGTGCAATAATAGTGTCAATAATGGCTAGAATTAAATAAGCATGTCTA

8941 TACTGTGAGGATTGACCTAGATTTCTTTCTATATAAAGATATTGGAAAATCAGGAAATAA
9001 TTTTTTTTTTATTTATTCCAGAAAAATTATAATTTAAATGGTATTTTTAAAATAGTATTT
9061 TGCCACACATAAAAGTCCTATATGACACTATGTATATTACTCTAAATTAATAGATTAAAA
9121 AATTATTATTATTATTTGGTGAGGATGGTAACTTTCTCTATTTCTGAGCAATTATAAGGA
9181 CACACCAAATATTAAACATGCCAGATGAGAATCTTTATTCCATGAAATATTAATAAACAG
9241 CCTAAGTTTAAATTTAGAGGAAAAAAAAGAAAAAATATTATTTTATTTTTAGAAGAATAT
9301 AAATATTCCATAAATAAGAATTAATAAATACATTTTAATATAAAAATAAAAATAAACATT
9361 AAACATCAAGAAATATACGTAAAGAAATTTATTCTTAACAATCATCAATCGTTTTAATAG
9421 AGCAATAGATTGTAATTCTGCAGATAGAGCACTCCATCCGATAACATTAAGTCATATATC
9481 GACTTATAGAGGTTTTTTGACTAGTTCAATTATTTGAATATTGAATTTAATTTCTTTTTT
9541 CTCTTCACCACTCGTACAGATAGAACTCAATACCTTCATGGTGGATTATAAATCTTTTAT
9601 AAAATGAGAACTGAACACTCTTATAATGAAAGATAATAAAATTTATAAAAAATATTAATA
9661 TTTATTTATTAAAATTAANNNNNNNNNNNNNNNNNNNNNNNNNNNNNNNNNNNNNNNNNN
9721 NNNNNNNNNNNNNNNNNNNNNNNNNNNNNNNNNNNNNNNNNNNNNNNNNNNNNNNNNNNN
9781 NNNNNNNNNNNNNNNNNNNNNNNNNNNNNNNNNNNNNNNNNNNNNNNNNACCCTATAGTT
9841 ATATAAATTATATATATATAACGTGACAATGATTTCTTTGAATCTTATATAAAGTGGAAG
9901 TAGCTGTTTCAATAAATGATCTAGAAGGTGATAATGGTTTCTTGAAATATAATATATATA
9961 TCACTAACCAATATTATAGTTGAAGAATCATTAATGAATTATTGTTATAAGTTATCAGTT
10021 GTAATAGTCCAATTCTTTGACTGGAATAGCACTGCATAAATGTGTCTATAAATGTGGCCA
10081 AAAAGTTAGCCATGAAAAGCAAAGGCATTagag**ATG**GCTGCTTATGGAGTTGTTCAAGAT
308 M A A Y G V V Q D
10141 GAAGAAAGCCTTAGTGGCAATAAGATACAACCGGTAGCCTCTACGCCAATgcaagtgttg
317 E E S L S G N K I Q P V A S T P M
10201 ttactttgtatctaatgttttttttttaataagaagaagtatataaactcaatttgttgc
10261 tcttttctgtttctcgttattaattgatcatatatgtattgattttcagGTCTGAGCAAC
334 S E Q Q
10321 AAAGCACCAGGGTAGGGAAGAAGCAAATCTCCCCCACATTGAGAAACATATTAGGCTTTG
338 S T R V G K K Q I S P T L R N I L G F E
10381 AAGAGCTTTTCTCTTTGACGgtaagtatgactttgtatagagtagaatctgcaaccccat
358 E L F S L T
10441 aaaattatcattttttttttttaattgcaaatgaaaaaggaactcaaatttgaatcaggt
10501 taagttgagatatatacatataatctatagaactatcatttcttagtagaactatataaa
10561 taataacatattttacatattaatgtatatagatacgaagatgaatacaatatattgtag
10621 ttcaacttttacttttaatttataaagaatgtgcaaattacttacttaaattgaaatata
10681 tacctacaactagttgaaatactagatatgttataaggttgacttaaagtaataattaaa
10741 tttcgactagtgcttttaggggatctctattgtttccatataaattttaattgtttttct
10801 ctcaccttgataatttcttaaaagatgacctttttgccaaatgcaaatgatattaaatcat
10861 taagagactagtttagaagtttaaggtatttaatttaaggaggacacacacatctaattg
10921 aacaattgggaccaaattaaactacatgtcaaaaagcaacttctgattaattttcgtcat
10981 cagagttatatccgaaaaatgagtgttgacataactcttcattattgatagtatagttca
11041 caatatcctttagatacatattacattaatgatttctttcttaatgtgagactcaaatat
11101 tacaatccatcacataataaacatggaaaagcaattcaattatttattactagctaacaa
11161 cctttttccatacaaaatgatatttccttttcagGTATGGAGAGCATCTTTGGCAGAACTC
364 V W R A S L A E L

11221 TTAGGCACTGCAATTCTTGTATTTGCAATAGACACTATAGTCATTTCCACTATAGAGAGC
373 L G T A I L V F A I D T I V I S T I E S
11281 GAAACAAAAGTACCAAACCTTATACTATCATGCCTTGTTGCCATCATAATAACAATTCTC
393 E T K V P N L I L S C L V A I I I T I L
11341 CTTATTGCTACATATCCAATATCTGGTGGTCACATTAATCCTATTGTTACCTTTTCTGCT
413 L I A T Y P I S G G H I N P I V T F S A
11401 GCACTCACTGGCCTTATTTCCATATCAAAAGCCTTCATATACATTTTGGCTCAATGTATT
433 A L T G L I S I S K A F I Y I L A Q C I
11461 GGTGGGATTGTGGGTGCACTAGCACTAAAAGCAGTAGTCAACAGCAACATTGAGAGAGTA
453 G G I V G A L A L K A V V N S N I E R V
11521 TTTTCACTAGGAGGTTGCACTCTTACTATTGTTGCACCAAGTGCTCATGGTCCTGTTACT
473 F S L G G C T L T I V A P S A H G P V T
11581 ATCGGTCTTCAAGTTGGCCAAGCCCTTTGGTTAGAGATTATCTGTGGGTTTGTTTTTCTT
493 I G L Q V G Q A L W L E I I C G F V F L
11641 TTTGCTTCAGTTTGGATGGCGTTTGATGACCGTCAAGCAAAGGCCCTGGGAAGAGTTGTG
513 F A S V W M A F D D R Q A K A L G R V V
11701 GTTTTCATTATTGTTGGGGTTGTCCTAGGCCTTCTTGTGTATGTCTCGACTTCGGTTACA
533 V F I I V G V V L G L L V Y V S T S V T
11761 ACTGCTAAAGGCTATGCTGGAGCTGGGATAAATCCTGCAAGGTGCTTTGGGCCAGCAATT
553 T A K G Y A G A G I N P A R C F G P A I
11821 GTCCGAGGTGATCATCTTTGGAATGGGCATTGGGTGTTTTGGGTGGGGCCATTTATTGCT
573 V R G D H L W N G H W V F W V G P F I A
11881 TGTGTGGCATTTACTTTGTACACTAAAATTATTCCTCCTCAACTTACTCACACAATG**TAG**
593 C V A F T L Y T K I I P P Q L T H T M *

(B)

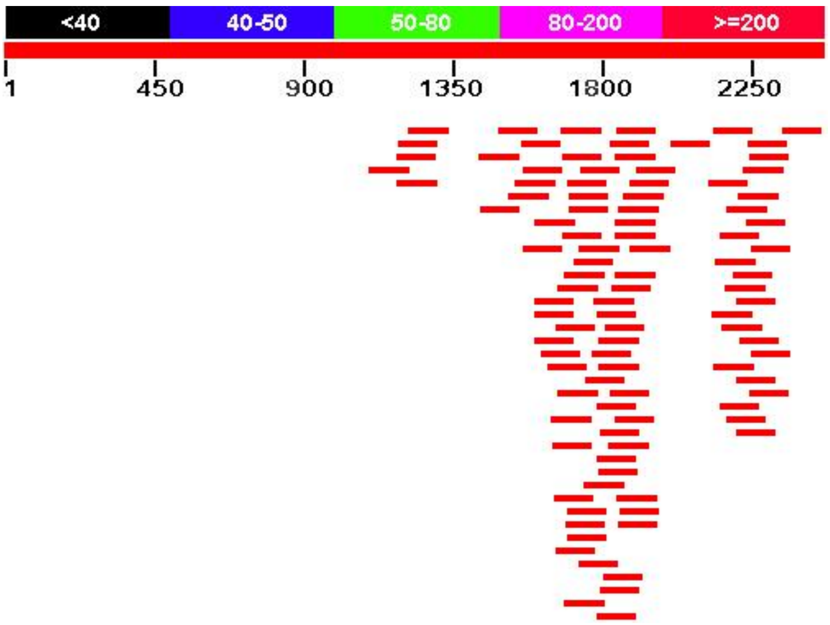

(C)

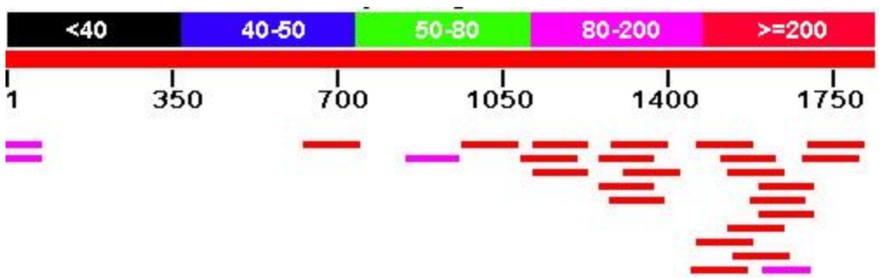
<40
40-50
50-80
80-200
>=200
1
350
700
1050
1400
1750
