# Supplementary material for: Gene Structures, Evolution, Classification and Expression Profiles of the Aquaporin Gene Family in Castor Bean (Ricinus communis L.)
Source: PLoS One. 2015 Oct 28;10(10):e0141022. doi: 10.1371/journal.pone.0141022 (PMC4625025; doi:10.1371/journal.pone.0141022)

S2 File. The gene model for *RcPIP2;5*. A, The coding region of the longest transcript is marked with uppercase letters, under which is its deduced amino acids. The transcribed untranslated regions, including 5' UTR, intron and 3' UTR sequences, are marked with lowercase letters. The start and stop codons are blacked. **B**, Diagrammatic presentation of four putatively alternative splicing transcripts.

(A)

1 tgatcacgcctccgcccatggttacgccatttttacagtttaagaagttaaatcttcaga
61 cttcatttctcttaaaaaaatttcaacaaaaacaagaaaaaagttaaaacagttatctct
121 atggttgtgacaatccgtgagcatgcagctgtagtatccttctttttgtcatctacaata
181 gagagaaataaacaaagaaacaattaaaagaataaggtaattaa**ATG**GCTGGTGATCAAG
1 M A G D Q D
241 ATAGACAGGTAGAAGAAGAAGAGGAGGAAAGTGACGGAAAGGACTACGTGGAGCCACCAC
7 R Q V E E E E E E S D G K D Y V E P P P
301 CAgttccattgttgcacttggcagagctgaaggaatgggcttttacagAGCTTTAATTGC
27 S F N C
361 GGAATTACTGCAACGCTTCTCTTTCTTTATGTGTCAGTAGCTACTGTTATCGGCCACAAG
31 G I T A T L L F L Y V S V A T V I G H K
421 GCTCAAATCTCTCCATGTGATGGTGTTGGCATTCTTGGCATTGCTTGGGCTTTTGGTGCC
51 A Q I S P C D G V G I L G I A W A F G A
481 ATGATTTTCGTTCTTGTCTATTGCACTGCTGGGATTTCTGgttggtacttcacatgatca
71 M I F V L V Y C T A G I S G
541 cccatcaagatcataacctttttgtttcttttctaataccatagacgtaggatttggcttt
601 ctgttttcctataaaacttggaaatgcaataacagattcatttttgtctggtttattatt
661 tatttcctacaaaactttagtttactatttcttttctgagtttaatcaaaaagggtaatt
721 gtctatatggcttgaaaaccgcttgcagGAGGGCACATCAACCCAGCAGTGACATTCGGG
85 G H I N P A V T F G
781 CTGCTGGTGGCGAGGAAGGTGTCACTGGATCGTGCAGTTTCTTACATGATATCGCAGTGC
95 L L V A R K V S L D R A V S Y M I S Q C
841 CTTGGAGCAATATGTGGCGTAGGGTTAGTGAAGGCATTCATGGAGCATGACTATATTAAT
115 L G A I C G V G L V K A F M E H D Y I N
901 ATTACACTTGGTGGTGGCGCAAACTCTGTTGCAACTGGTTACTCAAAAGGTGCTGCTTTG
135 I T L G G G A N S V A T G Y S K G A A L
961 GGTGCTGAGATTGTGGGTACTTTCGTTCTTGTGTACACAGTCTTTTCCGCCACTGATCCT
155 G A E I V G T F V L V Y T V F S A T D P
1021 AAGCGAAAAGCCCGCGACTCCCATGTTCCTgtaagtatatactagtagtaaaacaaataa
175 K R K A R D S H V P
1081 ctaaaactgaatttttttattaatctctctctccttggcaatccgggttaaatacttgta
1141 gaaagcacatcaggaatgtaatgctcaggctgatgcaggagagggtaatcttaccattgc
1201 caacaaggagacacccttgactgttaatgaaagaaaataataataattataataataata
1261 ataagagatgcataaaatggacttctctattttggagattctgatggggtgaggataaat
1321 gatttcagATACTGGCTCCTTTGCCAATTGGATTTGCTGTTTTCGCTGTGCATTTGGCCA

```
185          I  L  A  P  L  P  I  G  F  A  V  F  A  V  H  L  A  T
1381 CAATTCCTATTACTGGTACTGGCATAAATCCTGCCAGGAGCCTTGCTGCTGCTATTATTT
203    I  P  I  T  G  T  G  I  N  P  A  R  S  L  A  A  A  I  I  Y
1441 ACAACACTCGGAAAGTTTGGGAAGAACATgtatgtccattttcctctttcatctattata
223    N  T  R  K  V  W  E  E  H
1501 caaacatatgccaaagacaaagaacaataaacgaattatgctgtaaatcatgtattcaat
1561 tatcagtatctcacatgaaataaacaaatcaattcataattctacgaagggtagaaattt
1621 aaaaatataacttgctaaataagtgatttttttatttttattttaaaaaaaatacttatat
1681 atagatattaatatttttatttacacgctaaatcgatatataagttttatatatttatta
1741 atattaaataatatatagTGGATTTTCTGGGTGGGGCCATTAGTTGGAGCACTACTAGCA
232                    W  I  F  W  V  G  P  L  V  G  A  L  L  A
1801 GCAGTGTACTACCAGTACGTGATCAAAGGAGGAGCCATTAAATCTTTGGGATCATTCCGC
246  A  V  Y  Y  Q  Y  V  I  K  G  G  A  I  K  S  L  G  S  F  R
1861 AGCAGCAACCTTATGTGAggagacacccaattatctataatacttttagtcattcgtctg
266  S  S  N  L  M  *
1921 ttaatttaggaacttccttcaatcttattgctgtactgctttgagtttagacattttggt
1981 tctaatttttaaagatcactctgctactttcatcatctttgtagtataggaataatgcta
2041 caagtggtctgtaaattttatatttagtttcattttgattatgtaacttttttttttttgc
2101 ttcaattagtcaatctctaattatatttttatttaatcatatttgcaaatgaaaaaaataa
2161 taaataaaaaagagaatatataa
1981 t
```

(B)

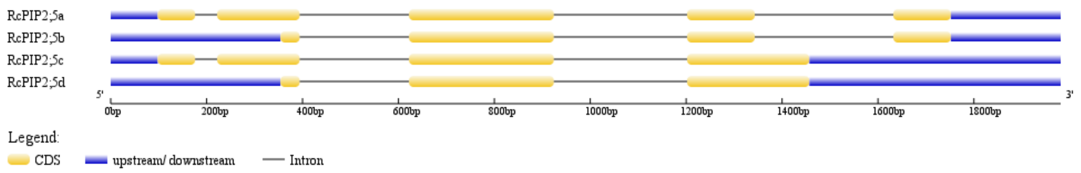

Supplement: S2 File — (PDF) [file pone.0141022.s002.pdf]
