# Supplementary material for: Gene Structures, Evolution, Classification and Expression Profiles of the Aquaporin Gene Family in Castor Bean (Ricinus communis L.)
Source: PLoS One. 2015 Oct 28;10(10):e0141022. doi: 10.1371/journal.pone.0141022 (PMC4625025; doi:10.1371/journal.pone.0141022)
Supplement: S3 File — (PDF) [file pone.0141022.s003.pdf]

S3 File. The gene model for *RcTIP2;2*. The coding region is marked with uppercase letters, under which is its deduced amino acids. The transcribed untranslated regions, including 5' UTR, intron and 3' UTR sequences, are marked with lowercase letters. The start and stop codons are blacked. The primers used for PCR amplification are labeled with single underlines.

1 gaagcactatgcttaagctgctgctgctatatatatatatatatattctccaaaatacat
61 gcttaacatatcatatacaggcttttaagtgtcccgctgtagctaatattgccaaggttg
121 ctagctagcaaaaacagaaggaaataaaa**ATG**GTGAAGATAGCTTTTGGTAGTTTAGGAG
1 M V K I A F G S L G D
181 ACTCTTTCAGCGCTGGATCCCTCAAGGCCTATCTATCTGAGTTCATTGCCACTCTTCTTT
12 S F S A G S L K A Y L S E F I A T L L F
241 TTGTGTTTGCTGGTGTTGGCTCTGCTATTGCTTACAgtaagtgattcaggaatccctttc
32 V F A G V G S A I A Y S
301 ttttgtttttcaggttcctgatatctaatattgtatttgatcgtaacaagaatatttgca
361 tgcatgctaccttagaaaataacaataatatatttttcttttcgtgtgtgaatattatag
421 GCAAGCTTACGACAGATGCAGCTCTAGACCCACCAGGGTTGGTTGCTGTGGCAGTGGCTC
44 K L T T D A A L D P P G L V A V A V A H
481 ATGCTTTCGGACTGTTTGTTGGGGTAGCCATCGCAGCCAACATCTCAGGTGGTCACTTAA
64 A F G L F V G V A I A A N I S G G H L N
541 ATCCAGCTGTCACCTTTGGTTTGGCAGTCGGAGGGAACATCACCATCCTAACTGGTATCT
84 P A V T F G L A V G G N I T I L T G I F
601 TCTACTGGATTGCCCAGTGCCTTGGCTCTATTGTTGCCTGCCTTCTTCTCCAATTTGTCA
104 Y W I A Q C L G S I V A C L L L Q F V T
661 CCAATGGCAAGgtatttgatacaacctatatattaagcctatacataagtgatacatata
124 N G K
721 tttattaacattaaaacattaaggtttatatcaattattggacgttcaagtattataagc
781 aaaattagataataaagtcctaaattgtgaaactgacatcttgggtttggcagAGCATCC
127 S I P
841 CAACCCATGGAGTTGCTTCAGGCATGAGTGCTATTGAAGGAGTAGTAATGGAGATTGTCA
130 T H G V A S G M S A I E G V V M E I V I
901 TAACCTTTGCACTAGTTTACACTGTCTATGCCACAGCAGCCGACCCCAAAAAGGGAGATT
150 T F A L V Y T V Y A T A A D P K K G D L
961 TGGGAATAATTGCACCCATTGCAATTGGGTTCATAGTGGGTGCAAACATATTAGCTGCCG
170 G I I A P I A I G F I V G A N I L A A G
1021 GCCCATTTAGCGGTGGCTCGATGAATCCGGCCCGATCATTTGGCCCAGCTGTGGTTAGCG
190 P F S G G S M N P A R S F G P A V V S G
1081 GTGACTTCTCAGAGAACTGGATCTATTGGGTCGGCCCACTAATCGGAGGAGGGTTAGCTG
210 D F S E N W I Y W V G P L I G G G L A G
1141 GGCTTGTATACAGTTGCAGTTTCATTGGGTCTTACTCTGCAGCCCCATCTTCTGAAGAGT
230 L V Y S C S F I G S Y S A A P S S E E Y
1201 ATGCC**TAA**attgggttgttaatgctttgtttgtgtggttctgtttcttggtttcttgtct

250 A *

1261 ttggggttgggcttggagaacttgtacttcataataaggaaaatgtaaataataaattga

1321 atcaagtttgagaatcttttgttgcatattt
