# Supplementary material for: Gene Structures, Evolution, Classification and Expression Profiles of the Aquaporin Gene Family in Castor Bean (Ricinus communis L.)
Source: PLoS One. 2015 Oct 28;10(10):e0141022. doi: 10.1371/journal.pone.0141022 (PMC4625025; doi:10.1371/journal.pone.0141022)

S4 File. The gene model for *RcNIP4;1*. **A**, The coding region is marked with uppercase letters, under which is its deduced amino acids. The transcribed untranslated regions, including 5' UTR, intron and 3' UTR sequences, are marked with lowercase letters. The start and stop codons are blacked. **B**, Read-mapping graphical representation of *RcNIP4;1*.

(A)

1 gcatttgagtagctctatccatccacgcatctatctcttattctttttcttttttaatc
61 tggtttcatataatttccttcctagacacaaacaccaaatctctctgcacacaggagaaa
121 taaaagtaaat**ATG**TCTGGAGAAAATCATGTCAGAAGCTTGGAAGAGGGGCAATGCAGCG
1 M S G E N H V R S L E E G Q C S D
181 ACTTTGTTCCTCCAAATAATAGCAAATCTGATTTTTGTTCTTCAAATGCAACAGTTCAAC
18 F V P P N N S K S D F C S S N A T V Q L
241 TCCTTCAGATGgcaaacaatctattgctatttactttctgtttttccccatttttttcgtt
38 L Q M
301 cattatattctaaatgaatattacatgaagaagaaactagcaagtaaaatggaatcaata
361 gttaggtggatcttaaaatatatatataaaagaatcaataattttactaatatgtgatac
421 attggcttggtaccctagatgttaataattttactaatatgtgcatctctttttctctaa
481 tagTTGATCGCTGAGACAATAGGGACATATTTGGTAATATTTTGCGGGTGCGGATCAGTC
41 L I A E T I G T Y L V I F C G C G S V
541 GCTGTGAATAAGATATATGGTTCGGTCACCTTTCCAGGAATTTGTGTAGTTTGGGGTCTA
60 A V N K I Y G S V T F P G I C V V W G L
601 ATTGTGATGGTTATGGTTTACTCTGTTGGTCATATCTCTGGTGCACATTTCAATCCTGCA
80 I V M V M V Y S V G H I S G A H F N P A
661 GTTACCATCACTTTCGCCATTTTTCGACAGTTTCCTTACAAACAGgcaagtctttctatc
100 V T I T F A I F R Q F P Y K Q
721 acattgatccttttatttgcaccaaactaaaatctgctagatattgcaaatcaaaatcct
781 tttctacttctttcttttcttttttggttttattaatttggaaccgtaacccgatagtat
841 tgagtaaaagtgcaacaataacgagccgagttctgaaataatatatagagatttatatac
901 tttcagGTGCCGATATACATTGTTGCACAAGTTGTTGGATCACTTCTTGCTAGTGGTACT
115 V P I Y I V A Q V V G S L L A S G T
961 TTATACTATATATTCAGTGTGACAGATGAGGCATTCTTTGGGACAGTACCAGTAGGACCT
133 L Y Y I F S V T D E A F F G T V P V G P
1021 CCTATGCGTTCTTTTGTTTTAGAAATAATTATCTCCTTCCTCTTAATGTTCGTTATTTCT
153 P M R S F V L E I I I S F L L M F V I S
1081 GGCGTGGCTACAGACAATAGAGCAgtgagtataaatttatttatcctatctcgatcacta
173 G V A T D N R A
1141 tatgcagtatcttatctcaataaggaactaaattatttaactcgttgcagATAGGGGAAT
181 I G E L
1201 TAGCAGGAATTGCTGTTGGAATGACAATTATGTTAAATGTCTTCATTGCTGGgtgggtac
185 A G I A V G M T I M L N V F I A G
1261 attctgctaatctaataacttttttttttctttgtaatcatagattaattccaaaagttt
1321 tctaagagcttacacttcacaaatgcagGCCTGTATCTGGGGCATCCATGAATCCTGCTA
202 P V S G A S M N P A R

1381 GAACCTTGGGGCCTGCCATTGTCATGCGAACATATAAAGGAATTTGGGTTTATATGGCTG
213 T L G P A I V M R T Y K G I W V Y M A G
1441 GTCCTGTCATTGGTGCCATTCTCGGAGGATTTGCTTATAATTTGATTAGGTTTACAGATA
233 P V I G A I L G G F A Y N L I R F T D K
1501 AACCTTTGCGCGAAATTACCAAGAGCAGCTCATTTATGAAGAGCTTTAGGGGT**TAA**aaat
253 P L R E I T K S S S F M K S F R G *
1561 ggttgttcgttgtaccatcttgtaacagcattacaagtataccaagcttgttagatttat
1621 acgattgactgaatttaaaattctagcaatttctcacatgcgccaaggttgtgttgtcct
1681 gggatccttgtgctgtttaccatcattaacataaatcctgatgcaaagaaagtaagtaga
1741 agattcagatctccaagtgatattgttttcttgcagcagactttctaaaatttatgatca
1801 ctttcattggatgcttgtggtcataaatgtggatctccttaacgatatatatggtacata
1861 aaggactcatcacatgctcccatccatgaatataataacacccaggaaagtatacataaa
1921 accaaaaatgttgtgacacacatggcgtcacatgcaacaaatactaacagaaatatcaca
1981 tggcttgccaaatgtgaaactgcccatctcccctggccggttttgaaacgtgaccctctt
2041 aaaaacaatttcaggaatttgctttatgtaatacagaagatcaagaattcggttttcact
2101 tatgaaaacattattctagtagtaaactgataatatttgtcttgcaattcaagaaatcca
2161 cactatttaaacataatagttaacaatctctcgattcctttcgtttaaagattttcaatc
2221 taaataaaatgaaattataaattaggtagcaccattgataatgaccgatggtccatagtc
2281 catagtccatcccttcccttgccaaacgaaatttcacaacaacctgaagcccacctctg
2341 ttgtttctattcatgcttgcatactttatagttacaaccatggatgcttccagctgatg
2401 tagatctcttaccagaagaaatagccatacatattataatgtatgtttcttgaagggaa

(B)

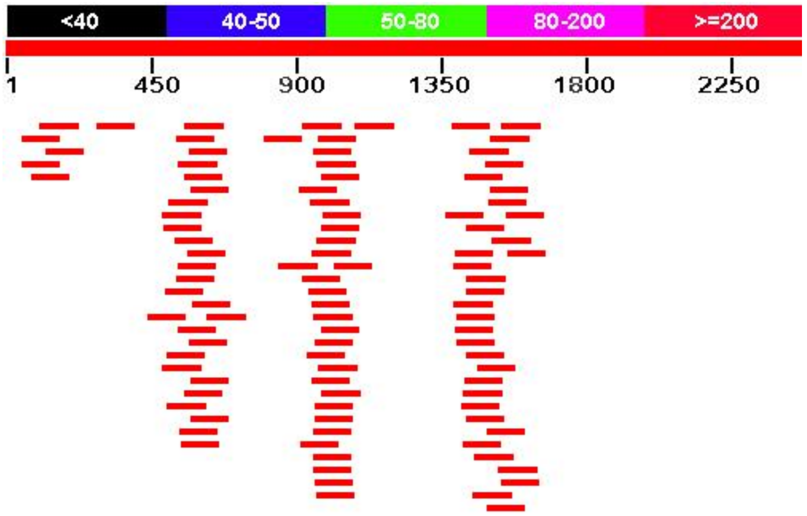

Supplement: S4 File — (PDF) [file pone.0141022.s004.pdf]
