# Supplementary material for: Gene Structures, Evolution, Classification and Expression Profiles of the Aquaporin Gene Family in Castor Bean (Ricinus communis L.)
Source: PLoS One. 2015 Oct 28;10(10):e0141022. doi: 10.1371/journal.pone.0141022 (PMC4625025; doi:10.1371/journal.pone.0141022)
Supplement: S5 File — (PDF) [file pone.0141022.s005.pdf]

S5 File. Alignment of deduced amino acids sequences of castor bean AQPs with structure determined Spinach PIP2;1. Multiple alignments were performed using ClustalX. Transmembrane helices (TM1–TM6) and the two short helices forming the two NPAs (HB and HE) (shaded), P_1–P_5 residues (shown in blue), NPA motifs (shown in red), and ar/R selectivity filter residues (shown in green) are indicated. The highly conserved cysteine residues in XIPs are shown in **bold**. The positions corresponding to S115 and S274 in SoPIP2;1（GenBank accession number 1Z98）are highlighted in bright green. The residues corresponding to S262 in GmNOD26 (GenBank accession number P08995) are shown in sky blue. The residues at the position corresponding to L197 from SoPIP2;1, determined to be the key residue involved in gating (occurring just before TM5) are underlined.

```
SoPIP2;1        --------M-SKEVSEE--------------AQAHQHGKDYVDPPPAPFFDLGEL-----
RcPIP1;1        --------MEGKEEDVRLGANKYRETQPIGTAAQSQDDKDYTEPPPAPLFEPGEL-----
RcPIP1;2        --------MEGKEEDVRLGANKFTERQPIGTSAQT--DKDYKEPPPAPLFEPGEL-----
RcPIP1;3        --------MEGREEDVSVGANRYGERQPIGTAAQTQDVKDYSEPPPAPLFEPGEL-----
RcPIP1;4        --------MEGKEEDVRLGANKYRETQPIGTAAQSQDDKDYTEPPPAPLFEPGEL-----
RcPIP1;5        --------MEGKEEDVTLGANRFPERQPIGTAAQT--EKDYNEPPSAPLFLPGEL-----
RcPIP2;1        --------M-GKDVEVG-------------GHGGEFHAKDYHDPPPAPLIDAEEL-----
RcPIP2;2        --------M-VKDMEVG--------------ERGPFSAKDYHDPPPAPLIDAVEL-----
RcPIP2;3        --------M-AKDVEVAE------------ATGGEFSAKDYHDPPPAPLIDVDEL-----
RcPIP2;4        --------M-AKDVGEE--------------TQT-SHGKDYVDPPPAPLVDMAEL-----
RcPIP2;5        --------M-AGDQDRQV-----------EEEEEESDGKDYVEPPP--------------
RcTIP1;1        ----------------------------------MPIRNIAVGHP-HEATQPDA------
RcTIP1;2        ----------------------------------MPISRIAVGNP-GEASQPDA------
RcTIP1;3        ----------------------------------MAITSIAFGSP-AEAGQPDA------
RcTIP1;4        ----------------------------------MPITRIAIGLPQHDILQSDA------
RcTIP2;1        ------------------------------------MAGIAFGRF-DDSFSLGS------
RcTIP2;2        ------------------------------------MVKIAFGSL-GDSFSAGS------
RcTIP3;1        -----------------------------------MPRRYAFGRA-EEATHPDS------
RcTIP4;1        ------------------------------------MAKIALGTR-REATQSDC------
RcTIP5;1        ------------------------------------MAPTSLLARFEQSVTRDA------
RcNIP1;1        ---------------------------------MAEISGNGNHGVVLDIKDVNP------
RcNIP2;1        ---------------------------METIIDPNLNNSSSSPASPEHLVSVEN------
RcNIP3;1        ---------------------------MASPNSITSEVSSKIQLPIKHSIPTEA------
RcNIP4;1        -------------------------------MSGENHVRSLEEGQCSDFVPPNN------
RcNIP4;2        -------------------------------------MTNIEEGLVVTSTKSNP------
RcNIP5;1        ----------------------MPESEAGTPTVSAPNTPGTPGGPLFSALRIDSL-----
RcNIP6;1        ----------------------MDNEEVPS-APSTPATPGTPGAPLFGGFKGDRGNGNGV
RcNIP7;1        --------------------MKMKHLLEEQPSPDTFMNASSSDASRDCSQDTGSN-----
RcXIP1;1        -----------------MALGAENRAFSDSGQALNVMGRQFPNSSFLTRLGVHEL-----
RcXIP1;2        -----------------MALGAENQACS------NLKGRQSPNSSFCTRIGVHEL-----
RcXIP1;3        ------------------------------------------------------------
RcXIP1;4        --------------------MSSQQAAN------VKDSSKYAMTTFLARIGAHEF-----
RcXIP2;1        MADNLRVIADEENGYGGRRVQPFASTPLGAALDNTNGGKKQNPTTFSRVLGLEEL-----
RcXIP3;1        MAAYG-VVQDEES-LSGNKIQPVASTPMSEQQS-TRVGKKQISPTLRNILGFEEL-----
RcSIP1;1        ------------------------------------------------------------
RcSIP1;2        ------------------------------------------------------------
RcSIP1;3        ------------------------------------------------------------
RcSIP2;1        ------------------------------------MGGGVTG-----------------
```

```
                                                                       TM1
SoPIP2;1     -------------------------------------KLWSFWRAAIAEFIATLLFLYIT
RcPIP1;1     -------------------------------------TSWSFYRAGIAEFIATFLFLYIS
RcPIP1;2     -------------------------------------SSWSFYRAGIAEFMATFLFLYIT
RcPIP1;3     -------------------------------------SSWSFYRAGIAEFVATFLFLYIT
RcPIP1;4     -------------------------------------TSWSFYRAGIAEFIATFLFLYIS
RcPIP1;5     -------------------------------------KSWSYYRAGIAEFMATFLFLYIT
RcPIP2;1     -------------------------------------TKWSFYRAIIAEFIATLLFLYIT
RcPIP2;2     -------------------------------------TKWSFYRALIAEFIATLLFLYIT
RcPIP2;3     -------------------------------------GKWSFYRALIAEFIATLLFLYIT
RcPIP2;4     -------------------------------------KLWSFYRALIAEFIATLLFLYIT
RcPIP2;5     ----------------------------------------SFN----CGITATLLFLYVS
RcTIP1;1     ------------------------------------------LKAALAEFISTLIFVFAG
RcTIP1;2     ------------------------------------------LRAALAEFFSMIIFVFAG
RcTIP1;3     ------------------------------------------LKAALAEFISMLIFVFAG
RcTIP1;4     ------------------------------------------LKAALAEFFSTAIFVFAG
RcTIP2;1     ------------------------------------------FKAYLAEFISTLLFVFAG
RcTIP2;2     ------------------------------------------LKAYLSEFIATLLFVFAG
RcTIP3;1     ------------------------------------------IRATLAEFVSTLIFVFAG
RcTIP4;1     ------------------------------------------IKALIVEFITTFLFVFAG
RcTIP5;1     ------------------------------------------LRSYLAEFISTFFFVFAV
RcNIP1;1     -----PPSASKDSVLSFSVP---------------------FMQKLIAEMVGTYFLIFAG
RcNIP2;1     -----PKSEKSFLCLVQSFQNQ---------------YPPRFPRKVVAEVIATYLLVFVT
RcNIP3;1     -----KASRSREWFVTDDAS-------------------PSVFQKIVAELVGTYILIFVG
RcNIP4;1     -----SKSDF--CSSNATVQ---------------------LLQMLIAETIGTYLVIFCG
RcNIP4;2     -----NPTSFGTCLSPKSVN---------------------IAQKVVAELIGTYFVIFAG
RcNIP5;1     SYDRKSMPR-CKCFPVNAPTFG----PPHTCFTDFPAPDISLTRKLGAEFVGTFILIFAA
RcNIP6;1     GFGRKSLLKSCKCFSVEEWSLEEGRLPPVSCS--LLPPPVSLARKVGAEFIGTLILMFAG
RcNIP7;1     -----ALSTNGDIFAKYSNFGCFP-----------KELDLNPARMVLAEFMGTFILMFCV
RcXIP1;1     -------------------------------------YSPEMWRAAFTELVASATFLFTL
RcXIP1;2     -------------------------------------YSLEMWRAAVTELVASATFLFTL
RcXIP1;3     -----------------------------------------MWRAAVTELVATGTFLFTL
RcXIP1;4     -------------------------------------FSPEMWRAVATELVATACLLFML
RcXIP2;1     -------------------------------------SSLNVWRASVAEVLGTAALVFAT
RcXIP3;1     -------------------------------------FSLTVWRASLAELLGTAILVFAI
RcSIP1;1     ---------------------------------------MGVIKSAVGDAVLTSMWVFIA
RcSIP1;2     ---------------------------------------MGVIKSAVGDAVLTSIWVFTL
RcSIP1;3     ---------------------------------------MRAIKAAAGDAVLTFMWVFCS
RcSIP2;1     -------------------------------------------RLIISDFVIAFMWVWSG
                                                                     ::
```

```
                    TM1                    TM2               HB
SoPIP2;1   VATVIGHSKETV------VCGSVGLLGIAWAFGGMIFVLVYCTAGISGGHINPAVTFGLF
RcPIP1;1   VLTVMGVVKAPT------KCSTVGIQGIAWAFGGMIFALVYCTAGISGGHINPAVTFGLF
RcPIP1;2   VLTVMGVSKSGN------KCATVGTQGIAWAFGGMIFALVYCTAGISGGHINPAVTFGLF
RcPIP1;3   VLTVMGVVKSPS------KCSTVGIQGIAWAFGGMIFALVYCTAGISGGHINPAVTFGLF
RcPIP1;4   VLTVMGVVKAPT------KCSTVGIQGIAWAFGGMIFALVYCTAGISGGHINPAVTFGLF
RcPIP1;5   VLTVMGYNRSPN------KCASVGVQGIAWAFGGMIFALVYCTAGISGGHINPAVTFGLL
RcPIP2;1   VLTVIGYKSQTDPAKNADACGGVGILGIAWAFGGMIFILVYCTAGISGGHINPAVTFGLF
RcPIP2;2   VLTVIGYKSQSE----SDSCGGVGILGIAWAFGGMIFILVYCTAGISGGHINPAVTFGLF
RcPIP2;3   VLTVIGYKSQTDPDKNADACGGVGILGIAWAFGGMIFILVYCTAGISGGHINPAVTFGLF
RcPIP2;4   VATVIGYKKQTD------PCGGVGILGIAWAFGGMIFILVYCTAGISGGHINPAVTFGLF
RcPIP2;5   VATVIGHKAQIS------PCDGVGILGIAWAFGAMIFVLVYCTAGISGGHINPAVTFGLL
RcTIP1;1   EGSGMAFNKLTN----NGAATPSGLVAAAIAHAFALFVAVSVGANISGGHVNPAVTFGAF
RcTIP1;2   EGSGMAFNKLTD----GGSTTPSGLVAASLSHAFALFVAVSVGANISGGHVNPAVTFGAF
RcTIP1;3   EGSGMAFNKLTS----DGATTPAGLVAASLAHGFALFVAVSVGANISGGHVNPAVTFGAF
RcTIP1;4   EGSSMAFSKLTD----DGSSTPAAIIMASLAHAFGLFVGVSTAANISGGHCNPAVTFGAF
RcTIP2;1   VGSAIAYGKLTS----DAALDPAGLVAIAICHGFALFVAVAVGANISGGHVNPAVTFGLA
RcTIP2;2   VGSAIAYSKLTT----DAALDPPGLVAVAVAHAFGLFVGVAIAANISGGHLNPAVTFGLA
RcTIP3;1   EGSVLALDKLYR----ETGPPASGLVMVALAHGLALFSAVSASINISGGHLNPAVTFGAL
RcTIP4;1   VGSAMAANKLLG----DS---LVGLFFVAMAHTLVVAVMISAG-HISGGHLNPAVTLGLL
RcTIP5;1   VGSSMASRKLMP----AAD--PSNLVIVALANAFALSSAVYIAANISGGHVNPAVTFSMA
RcNIP1;1   CTSVAVN---------LNFDKVVTLPGISIVWGLAVMVLVYSVGHISGAHFNPAVTLAFA
RcNIP2;1   CGAAAIS---------SADDKRISKLGASLAGGLIVTVMIYAVGHVSGAHMNPAVTTAFA
RcNIP3;1   CGVALTD---------EVQR--LTMVGIAIAWGVVLMALIYAVGHVSGAHFNPAVSIAFA
RcNIP4;1   CGSVAVN---------KIYG-SVTFPGICVVWGLIVMVMVYSVGHISGAHFNPAVTITFA
RcNIP4;2   CGSVAVN---------NIYG-SVTFPGVCVTWGLIVAVMIYSVGHISGAHFNPAVTITSA
RcNIP5;1   TAGPIVN---------QKYNGVETLIGNAACAGLAVMIIILSTGHISGAHLNPSLTIAFA
RcNIP6;1   TATAIVN---------QKTQGTETLIGLAVSTGLAVMIVILSTGHISGAHLNPSITIAFA
RcNIP7;1   CGIMAST---------QLTGGQVGLLEYAATAGLTVIVLVFAIGPISGAHVNPAVTIAFA
RcXIP1;1   STTIIACLESHE---------TAPKLVIPVAVFFIALFWLLPTVPLSGGFFSPTFTFMAA
RcXIP1;2   STTIIACLESHE---------TSPKLVIPVVVFLIAFFWLLTTVPLSGGFFSPTFAFMAA
RcXIP1;3   STTIIACLESHE---------SDPKLLIPIAVFFIAFLWLMVTVPLSGGLFSPAFSFIAA
RcXIP1;4   TTTIIACLESKE---------TEPKLLIPIVVFVIVFLVLVVTVPLSGGHMSPVFTFIAA
RcXIP2;1   DTIVISTYETET---------KTPNLIMAALIAMTVTILLTATFPISGGHINPVITISAA
RcXIP3;1   DTIVISTIESET---------KVPNLILSCLVAIIITILLIATYPISGGHINPIVTFSAA
RcSIP1;1   PFLGVLTSIIASYVG-IEPRSVPALFITINLATLRLLTFSFIGALLGGASFNPNTTVSLY
RcSIP1;2   PFLGVLTSIVSTYVG-VEPRSIPGLFITINLATLLYLMFSFLGAALGGASFNPATTVTLY
RcSIP1;3   SLFGFFTTLIATALG-VQHHVWATLFITTVLVFVFVFLFGLIAEFLGGASFNPTGTASFY
RcSIP2;1   ALIKMFVNGVLR-----MGHEPSGEVLKSTLSIINMFFFAFLGKISKGAAYNPLTIFSSA
                                                    *.   .*
```

TM3

```
SoPIP2;1     LAR---KVSLLRALVYMIAQCLGAICGVGLVKAFMK-GPYN--QFGGGANSVALG-----
RcPIP1;1     LAR---KLSLTRALFYMVMQCLGAICGAGVVKGFEGSHDYTR--LGGGANSVNPG-----
RcPIP1;2     LAR---KLSLTRALFYMVMQCLGAICGAGVVKGFEGDRTYET--LGGGANVVNAG-----
RcPIP1;3     LAR---KLSLTRAVFYMIMQCLGAICGAGVVKALEKGHEYER--LGGGANTVSSG-----
RcPIP1;4     LAR---KLSLTRALFYMVMQCLGAICGAGVVKGFEGSHDYTR--LGGGANSVNPG-----
RcPIP1;5     LAR---KLSLNRAIFYMVMQCLGAICGAGVVKGFQP-TPYER--VGGGANMVNPG-----
RcPIP2;1     LAR---KVSLVRAIMYMAAQCLGAICGCGLVKAFQR-AYYN--RYGGGANELADG-----
RcPIP2;2     LAR---KVSLVRAIMYMVAQCLGAICGVGLVKAFQS-SHYK--RYGGGANTLDDN-----
RcPIP2;3     LGR---KVSLIRALGYMVAQCLGAICGCGLVKAFQK-AYYN--RYGGGANELADG-----
RcPIP2;4     LAR---KVSLIRAVAYMVAQCLGAICGVGLVKAFMK-NPYN--RLGGGANSVASG-----
RcPIP2;5     VAR---KVSLDRAVSYMISQCLGAICGVGLVKAFME-HDYINITLGGGANSVATG-----
RcTIP1;1     VGG---NITLLRGILYWIAQLLGSTVACLLLKFSTGGLTTSAFALSSG------------
RcTIP1;2     IGG---HITLLRGILYWIAQLLGSVVACLLLKFSTGGLETSAFALSSG------------
RcTIP1;3     VGG---HITFIRSILYWIAQLLGSVVACLLLKFSTGGLETSAFALSSG------------
RcTIP1;4     LGG---NISLLRGILYWIAQLLGSTVACLLLKFSTHGMTTSAFALSSG------------
RcTIP2;1     LGG---QITILTGIFYWIAQLLGSIVACFLLKVVTGGLATPIHSVAAG------------
RcTIP2;2     VGG---NITILTGIFYWIAQCLGSIVACLLLQFVTNGKSIPTHGVASG------------
RcTIP3;1     VGG---RISVVLAFYYWIAQLLGAIVASLLLRLVTNGMRPVGFHVTSG------------
RcTIP4;1     AGG---HITVVRSILYWIDQLLASSAACFLLNYLTGGMATPVHTLASG------------
RcTIP5;1     IGG---HISVPTALFYWVSQLVASVMACLLLRVAAVGQNLPTYIIAEE------------
RcNIP1;1     TCK---RFPWKQVPAYIACQVIGSTLAAGTIRLIFTGKQDHFTGTMP-------------
RcNIP2;1     AVR---HFPWKEVPYYAAAQLTGAISASFTLKVLLHP-VKHIGTTSP-------------
RcNIP3;1     AGR---KFPWKHVPMYILAQVLGSTLASLTLRVLFNDLDDIEVTVTQYKD----------
RcNIP4;1     IFR---QFPYKQVPIYIVAQVVGSLLASGTLYYIFSVTDEAFFGTVP-------------
RcNIP4;2     IFH---RFPMHEVPLYIVAQVMGSILASGTLALVVDVNPKAYFGTVP-------------
RcNIP5;1     ALR---HFPWVQVPAYIAAQVSASICASFALKGVFHPFMSGGVTVPS-------------
RcNIP6;1     ALR---HFPWKHVPVYIGAQVSASVCAAFALKVIFHPFMSGGVTVPS-------------
RcNIP7;1     TFG---HFPWSKVPFYVVAQTVGSVLATYAAKLVYGIKADLMVTRPV-------------
RcXIP1;1     LRG---VISFTRALFYCLGQCLGAIIGYIILKSVMDPTIAHKYALGGCMVNGNGE-----
RcXIP1;2     LSG---VISFVRALFYCLGQCFGAIIGYMILKSVMDPTIAHKYALGGCMVNGGGE-----
RcXIP1;3     LRG---VITFVRALFYSLGQLLGALIAYLILKGVMDPNMAHKYALAGCMVNGNGA-----
RcXIP1;4     LRG---LISLVRALFYVLAQCVGSIMAYLVIKSVMDETVADKYALGGCMVNGNGA-----
RcXIP2;1     FTG---LVSPVRAAVYILAQCLGATLGALALKAVVNSRIEETFSLGGCTLNIVAPGPQGP
RcXIP3;1     LTG---LISISKAFIYILAQCIGGIVGALALKAVVNSNIERVFSLGGCTLTIVAPSAHGP
RcSIP1;1     AAGLKSDMSLVSMAVRFPAQAAGGAGGATAMKILQALPRKYHHMLKGPTLN---------
RcSIP1;2     ASGLRPDASLMSMAVRFPAQAAGGVSG--AMAILQAMPRKYKHLLKGPSLK---------
RcSIP1;3     AAGATADN-LFSMALRFPAQAAGAVGG--ALAILEVMPLQYKHMLGGPALK---------
RcSIP2;1     ISG-DFSQFLLTVGARIPAQVIGSITG---VTLVIQTFPEIGFGPRLN------------
                                  *   ..  .
```

```
                     TM4                                  TM5
SoPIP2;1    ----YNKGTALGAEIIGTFVLVYTVFSATDPKRSARDSHVPILAPLPIGFAVFMVHLATI
RcPIP1;1    ----YTKGDGLGAEIVGTFVLVYTVFSATDAKRSARDSHVPILAPLPIGFAVFLVHLATI
RcPIP1;2    ----YTKGDGLGAEIVGTFVLVYTVFSATDAKRNARDSHVPILAPLPIGFAVFLVHLATI
RcPIP1;3    ----YSKGDGLGAEIVGTFILVYTVFSATDAKRNARDSHVPILAPLPIGFAVFLVHLATI
RcPIP1;4    ----YTKGDGLGAEIVGTFVLVYTVFSATDAKRSARDSHVPILAPLPIGFAVFLVHLATI
RcPIP1;5    ----YSKGDGLGAEIVGTFVLVYTVFSATDAKRSARDSHVPVLAPLPIGFAVFLVHLATI
RcPIP2;1    ----YSTGTGLGAEIIGTFVLVYTVFAATDPKRNARDSHVPVLAPLPIGFAVFMVHLATI
RcPIP2;2    ----YSTGVGLGAEIIGTFVLVYTVFSATDPKRSARDSHVPVLAPLPIGFAVFMVHLATI
RcPIP2;3    ----YNKGTGLGAEIIGTFVLVYTVFSATDPKRSARDSHVPVLAPLPIGFAVFMVHLATI
RcPIP2;4    ----YSNGTALGAEIIGTFVLVYTVFSATDPKRSARDSHVPVLAPLPIGFAVFMVHLATI
RcPIP2;5    ----YSKGAALGAEIVGTFVLVYTVFSATDPKRKARDSHVPILAPLPIGFAVFAVHLATI
RcTIP1;1    ----VGVWNAFVFEIVMTFGLVYTVYATAVDPKKG---SLGTIAPIAIGFIVGANILAGG
RcTIP1;2    ----VSSWNAVVFEIVMTFGLVYTVYATAVDPKKG---NIGIIAPIAIGFIVGANILAGG
RcTIP1;3    ----VGAWNAVVFEIVMTFGLVYTVYATAVDPKKG---NIGIIAPLAIGFIVGANILAGG
RcTIP1;4    ----VNVWNAFVFEIVMTFGLVYTVYATAIDPKKS---EVGIIAPLAIGFVVGANILAGG
RcTIP2;1    ----VGAIEGVVMEIVITFALVYTVYATAADPKKG---SLGTIAPIAIGFIVGANILAAG
RcTIP2;2    ----MSAIEGVVMEIVITFALVYTVYATAADPKKG---DLGIIAPIAIGFIVGANILAAG
RcTIP3;1    ----VGEVHGLIMEMVMTFGLVYTVYATAIDPKRG---SLGIIAPLAIGFIVGANILVGG
RcTIP4;1    ----VGYVQGIVWEIVLTFSLLFTVYATIVDPKKG---SIDGLGPTLTGFVVGANILAGG
RcTIP5;1    ----MTGFGASIVEGVLTFGLVYTVYAAG-DPRRS---LLGATGPLVIGLMAGANVLAAG
RcNIP1;1    ---AGSDMQSFVVEFIITFYLMFIISGVATDNR-----AIGELAGLAVGATVLLNVMFAG
RcNIP2;1    ---SGSDFQALVMEIVVTFCMMFVTSAVATDTK-----AIGELAGIAVGSAVCITSILAG
RcNIP3;1    ---STSDLEAIIWEFIITFILMFNILAVATDYR-----AVKYLSGVAIGGTLLFNALLAG
RcNIP4;1    ---VGPPMRSFVLEIIISFLLMFVISGVATDNR-----AIGELAGIAVGMTIMLNVFIAG
RcNIP4;2    ---VGSNWQSLIMEIIITFLLMFVISGVTTDDRT----TAGPLGGIGVGMTILLNVFVAG
RcNIP5;1    ----VSTGQAFALEFLITFNLLFVVTAVATDTR-----AVGELAGIAVGATVMLNILVAG
RcNIP6;1    ----GGYGQAFALEFIISFNLMFVVTAVATDTR-----AVGELAGIAVGATVTLNILIAG
RcNIP7;1    ----QGCNSAFSVEFITTFLMMFLAASLAYQAA------TRHLSGFVIGLSIGLAVLISG
RcXIP1;1    ---GVSAGTALMIEFSCTFLVLYTAMTIVLDKKKCQDLGLTMVCIIISGAYAVSVFVSTT
RcXIP1;2    ---GVSAGTALMVEFSCTFLVLYVAMTIILDKKKCQELGLTMVCVIISGAYAVSVFVSTT
RcXIP1;3    ---GVSVGTALIIEILCTFMVLYVAMTIILDKQKCMDLGLTTVCVIISGIYAASVFVSIT
RcXIP1;4    ---GVSTGTALVIEFACTFLVLYVAITVAFDKKMCQELGL---AILIR------------
RcXIP2;1    IVIGLETSQALWLEIICTFLFLFSSIWLAFDKRQAKLLGQVIVCSIIGLVVGLIVFISTT
RcXIP3;1    VTIGLQVGQALWLEIICGFVFLFASVWMAFDDRQAKALGRVVVFIIVGVVLGLLVYVSTS
RcSIP1;1    ----VDLHTGAVVG----FLYCLALLLVMTKGPKN-----FLLKIWLLAWAR---AVVGG
RcSIP1;2    ----VDLHTGAIAEGVLSFMLCLAFLSLMSKGPKN-----SMLKLWLLACVITGLAACGA
RcSIP1;3    ----VDLHTGAIAEGVLTFFISFAVLVIILRGPRN-----LFVQNWLIAVVTVALVVTGS
RcSIP2;1    ----VDIHRGALTEGLLTFAIVTISLGLARKIPGS-----FFMKTWISSVSKTLHILGS
```

```
                                       HE                          TM6
SoPIP2;1      P-----ITGTGINPARSFGAAVIFNSNKVWDDQWIFWVGPFIGAAVAAAYHQYVLRAAAI
RcPIP1;1      P-----ITGTGINPARSLGAAIIFNKDQGWDDHWIFWVGPFIGAALAALYHQVVIRAIPF
RcPIP1;2      P-----ITGTGINPARSLGAAIIFNKDHAWDDHWIFWVGPFIGAALAAVYHQIVIRAIPF
RcPIP1;3      P-----ITGTGINPARSLGAALIYNKDQAWDDHWIFWVGPFIGAALAALYHQIVIRAIPF
RcPIP1;4      P-----ITGTGINPARSLGAAIIFNKDQGWDEHWIFWVGPFIGAALAALYHQVVIRAIPF
RcPIP1;5      P-----ITGTGINPARSLGAAIIYNKDHAWDDHWIFWVGPFIGAALAALYHQIIIRAIPF
RcPIP2;1      P-----VTGTGINPARSFGAAVIYNKEKAWDDQWIFWVGPFIGAAIAAIYHQYVLRASAA
RcPIP2;2      P-----ITGTGINPARSLGAAVIYNQDKAWDDQWIFWVGPFIGAAIAAFYHQFILRAGAV
RcPIP2;3      P-----ITGTGINPARSFGAAVIYNKEKAWDDQWIFWVGPFIGAAIAAFYHQYILRAAAI
RcPIP2;4      P-----ITGTGINPARSFGAAVIYNNDKVWDDHWIFWVGPFVGALAAAAYHQYVLRAAGI
RcPIP2;5      P-----ITGTGINPARSLAAAIIYNTRKVWEEHWIFWVGPLVGALLAAVYYQYVIKGGAI
RcTIP1;1      A-----FDGASMNPAVSFGPALVSW---SWENHWVYWAGPLVGGGLAGLVYEFFFI-HS-
RcTIP1;2      A-----FDGASMNPAVSFGPAVVSW---TWTNHWVYWVGPLIGSAIAAIVYDNIFIGYG-
RcTIP1;3      A-----FDGASMNPAVSFGPAVVSW---TWDNHWVYWLGPFVGAGIAAIVYETFFISPS-
RcTIP1;4      A-----FEGASMNPAVSFGPALVSW---DWTNHWVYWAGPLIGGGLAGIVYDLFFI-TY-
RcTIP2;1      P-----FSGGSMNPARSFGPAVVSG---DFTDNWIYWVGPLVGGGLAGLIYGNLYMPG--
RcTIP2;2      P-----FSGGSMNPARSFGPAVVSG---DFSENWIYWVGPLIGGGLAGLVYSCSFIGSY-
RcTIP3;1      P-----FDGASMNPARAFGPALVGW---RWNNHWIYWLGPFVGGGLAALIYEYMVIPTEP
RcTIP4;1      P-----FSGASMNPARSFGPALVSW---DWTDHWVYWVGPLIGGGLAGFIYENFFIIR--
RcTIP5;1      P-----FSGGSMNPACAFGSAVVAG---RFKNQAVYWVGPLLGATVAGLLYDNVVFPNQV
RcNIP1;1      P-----ISGASMNPARSLGPAIVSH---KYKGLWIYIVSPTLGAQAGAWVYNMIRYTDKP
RcNIP2;1      P-----ISGGSMNPARTLGPAIASA---YYKGIWVYIVGPVVGTLLGSWSYNFIRVTDQP
RcNIP3;1      P-----ITGASMNPARSLGPAIVSG---VYKNLWVFIVSPIFGALAATYVYNMLRVPEPE
RcNIP4;1      P-----VSGASMNPARTLGPAIVMR---TYKGIWVYMAGPVIGAILGGFAYNLIRFTDKP
RcNIP4;2      P-----VSGASMNPARSIGPAIVKH---VYKGLWVYIVGPIVGAILGASAYNLLR---SP
RcNIP5;1      P-----SSGGSMNPVRTLGPPAVAAG--NYRALWIYLVAPTLGAIAGAGTYSAVKLREEE
RcNIP6;1      E-----TTGASMNPVRTLGPAIAAN---NYKAIWVYLTAPILGALCGAGIYSAVKLPEED
RcNIP7;1      P-----VSGGSLNPARSLGPAIVSW---NFKDIWVYIIAPTTGAVAGALMFHVLRIQRPP
RcXIP1;1      VTGRVGYGGVGLNPARCLAPALLLG-GALWDGHWVFWVGPVCACSVYYVCSLMLPKQGFV
RcXIP1;2      VTGRIGYGGVGLNPARCLAPALLLG-GSLWDGHWVFWVGPICSCSVYYVFSLLLPKQGFV
RcXIP1;3      VTGRPGYGGVGLNPARCLGPVLLMG-GALWEGHWVFWVGPFCACMIYYAYSLMLPKRGFV
RcXIP1;4      -----QFG---------------------------------LKATIYYL-----------
RcXIP2;1      VTATKGYAGVGMNPARCLGPALVRG-GHLWNGHWVFWAGPVISCVAFAIYTKIIPKAEVH
RcXIP3;1      VTTAKGYAGAGINPARCFGPAIVRG-DHLWNGHWVFWVGPFIACVAFTLYTKIIPPQLTH
RcSIP1;1      E-----KSGTLMNPANAYGWAYVNNLHNTWEFFHVYWFCPFIGATLAAWNFRLLFKASA-
RcSIP1;2      K-----YTGPSLNPANVYGWAYVHNWHNSWELFYVYWIGPLVGATLSAWVFRFLFKPSS-
RcSIP1;3      K-----YTGPSMNPANAFGWAYINKWHNTWEQFYVYWISPFIGATLAAWIFRLIFPPPAP
RcSIP2;1      D-----LTGGVMNPASVMGWAYARGDHITKEHILVYWLAPVEATLLAVWTFKLLVRPTTQ
```

```
SoPIP2;1        KALGSFRSNPTN-----------------------------------
RcPIP1;1        KKC--------------------------------------------
RcPIP1;2        KSRA-------------------------------------------
RcPIP1;3        QSK--------------------------------------------
RcPIP1;4        KK---------------------------------------------
RcPIP1;5        KARA-------------------------------------------
RcPIP2;1        KALGSFRSSSNI-----------------------------------
RcPIP2;2        KALGSFRSNPTV-----------------------------------
RcPIP2;3        KALGSFRSNA-------------------------------------
RcPIP2;4        KALGSFRSNPTN-----------------------------------
RcPIP2;5        KSLGSFRSSNLM-----------------------------------
RcTIP1;1        ---THEQLPTTDY----------------------------------
RcTIP1;2        ---AHEPLPVNDF----------------------------------
RcTIP1;3        ---THEQLPSADF----------------------------------
RcTIP1;4        ---SHEPVPSSEL----------------------------------
RcTIP2;1        ---DHAPL-SSDF----------------------------------
RcTIP2;2        ---SAAPS-SEEYA---------------------------------
RcTIP3;1        PHHTHQPLAPEDY----------------------------------
RcTIP4;1        ---SHRPLPNDEENY--------------------------------
RcTIP5;1        P--DSIRGISDGVGA--------------------------------
RcNIP1;1        LREITKS--ASFLKSTGRA----------------------------
RcNIP2;1        LQAISPRSFSAKLRRIRSTNEQPTNKDPFDAL---------------
RcNIP3;1        KSEEKTKNIFNHLYTATDP----------------------------
RcNIP4;1        LREITKS--SSFMKSFRG-----------------------------
RcNIP4;2        YNQTP------------------------------------------
RcNIP5;1        VDP---PRPVRSFRR--------------------------------
RcNIP6;1        GDAREKPSTARSFRR--------------------------------
RcNIP7;1        CSPTTPSPNTGLLGHSINFARR-------------------------
RcXIP1;1        RADEEQHVLQLVRSSCLGSESANYFEGKV------------------
RcXIP1;2        RADEQQHLIQLVRSSCLGSESPDYFEGKV------------------
RcXIP1;3        RADIEEDIIKLVRASCSGSDCPSCVEKKVTSLYEKILYKLSFGFICD
RcXIP1;4        -----------------------------------------------
RcXIP2;1        A----------------------------------------------
RcXIP3;1        TM---------------------------------------------
RcSIP1;1        KHKQA------------------------------------------
RcSIP1;2        KQKQA------------------------------------------
RcSIP1;3        KQKKA------------------------------------------
RcSIP2;1        ERKENLKGKSD------------------------------------
```
