# Supplementary material for: Gene Structures, Evolution, Classification and Expression Profiles of the Aquaporin Gene Family in Castor Bean (Ricinus communis L.)
Source: PLoS One. 2015 Oct 28;10(10):e0141022. doi: 10.1371/journal.pone.0141022 (PMC4625025; doi:10.1371/journal.pone.0141022)
Supplement: S6 File — (PDF) [file pone.0141022.s006.pdf]

S6 File. SDP analysis of castor bean AQPs from alignments with amino acid sequences of AQPs transporting non-aqua substrates. Multiple alignments were performed using ClustalW. Shown is the sequences containing the nine SD positions which are highlighted in yellow and the representative sequences are marked in blue. The Genbank accession numbers: AtPIP1;2 (Q06611), AtPIP2;1 (P43286), AtPIP2;4 (Q9FF53), AtTIP1;1 (P25818), AtTIP1;2 (Q41963), AtTIP1;3 (NP_192056), AtTIP2;1 (Q41951), AtTIP2;3 (Q9FGL2), AtTIP4;1 (O82316), AtTIP5;1 (NP_190328), AtNIP1;1 (CAA16760), AtNIP1;2 (Q8LFP7), AtNIP5;1 (NP_192776), AtNIP6;1 (NP_178191), CmNIP2;1 (BAK09176), CpNIP1 (CAD67694), GmNOD26 (P08995), GmNIP2;1 (XP_003534451), GmNIP2;2 (NP_001240190), HvPIP1;3 (BAA23745), HvPIP1;4 (BAF33068), HvPIP2;1 (BAA23744), HvNIP2;1 (BAH24163), NtAQP1 (O24662), NtTIPa (Q9XG70), NtXIP1;1α (ADO66667), NtXIP1;1β (ADO66666), OsNIP2;1 (Q6Z2T3), OsNIP2;2 (Q67WJ8), OsNIP3;2 (Q7EYH7), SlXIP1;1α (ADO66672), SlXIP1;1β (ADO66671), StXIP1;1α (ADO66669), StXIP1;1β (ADO66670), TaLsi1 (ADM47602), TaTIP2;1 (AAS19468), TaTIP2;2 (AAS19469), TgTIP1;1 (BAL41683), TgTIP1;2 (BAL41684), ZmPIP1;1 (Q41870), ZmPIP1;5 (Q9AR14), ZmNIP2;1 (Q19KC1), ZmNIP2;2 (Q9ATN2).

Ammonia (NH_3) Transporters

```
RcTIP2;2          TGIFYWIAQCLGSIVACLLLQFVTNGKSIPTHGVASGMSAIEGVVMEIVITFALVYTVYA
RcNIP1;1          QVPAYIACQVIGSTLAAGTIRLIFTGKQDHFTGTMPAGSDMQSFVVEFIITFYLMFIISG
AtTIP2;1          TGVFYWIAQLLGSTAACFLLKYVTGGLAVPTHSVAAGLGSIEGVVMEIIITFALVYTVYA
AtTIP2;3          TGFFYWIAQCLGSIVACLLLVFVTNGKSVPTHGVSAGLGAVEGVVMEIVVTFALVYTVYA
TaTIP2;1          TGIFYWVAQLLGAIVGAFLVQFCT-GVATPTHGLS-GVGAFEGVVMEIIVTFGLVYTVYA
TaTIP2;2          TGIFYWVAQLLGAIVGAFLVQFCT-GVATPTHGLS-GVGAFEGVVMEIIVTFGLVYTVYA
GmNOD26           QVPAYVVAQLLGSILASGTLRLLFMGNHDQFSGTVPNGTNLQAFVFEFIMTFFLMFVICG
                      *  .* :*:  ..  :     *      .       .:..*.*:::** *:: : .

RcTIP2;2          TAADPKKGDLGIIAPIAIGFIVGANILAAGPFSGGSMNPARSFGPAVVSGDFSENWIYWV
RcNIP1;1          VATDNR--AIGELAGLAVGATVLLNVMFAGPISGASMNPARSLGPAIVSHKYKGLWIYIV
AtTIP2;1          TAADPKKGSLGTIAPLAIGLIVGANILAAGPFSGGSMNPARSFGPAVAAGDFSGHWVYWV
AtTIP2;3          TAADPKKGSLGTIAPIAIGFIVGANILAAGPFSGGSMNPARSFGPAVVSGDLSQIWIYWV
TaTIP2;1          TAADPKKGSLGTIAPIAIGFIVGANILVAGPFSGGSMNPARSFGPAVASGDFTNIWVYWA
TaTIP2;2          TAADPKKGSLGTIAPIAIGFIVGANILVAGPFSGGSMNPARSFGPAVASGDFTNIWIYWA
GmNOD26           VATDNR--AVGEFAGIAIGSTLLLNVIIGGPVTGASMNPARSLGPAFVHGEYEGIWIYLL
                  .*:* :   :* :* :*:*  :  *:: .**.:*.*******:***..  .    *:*

RcTIP2;2          GPLIGGGLAGLVYSCSFIGSYSAAPSSEEYA---------
RcNIP1;1          SPTLGAQAGAWVYNMIRYTDKPLREITKSASFLKSTGRA-
AtTIP2;1          GPLIGGGLAGLIYGNVFMGSSEHVPLASADF---------
AtTIP2;3          GPLVGGALAGLIYGDVFIGSYEAVETREIRV---------
TaTIP2;1          GPLIGGGLAGVVYRYLYMCD-DHSSVAGNDY----------
TaTIP2;2          GPLIGGGLAGVVYRYVYMCD-DHSSVAGNDY----------
GmNOD26           APVVGAIAGAWVYNIVRYTDKPLSETTKSASFLKGRAASK
                  .* :*.  .. :*      .       .
```

Boric Acid Transporters

```
RcPIP1;1        VGIQGIAWAFGGMIFALVYCTAGISGGHINPAVTFGLFLARKLSLTRAVFYMIMQCLGAI
RcPIP1;2        VGIQGIAWAFGGMIFALVYCTAGISGGHINPAVTFGLFLARKLSLTRALFYMVMQCLGAI
RcPIP1;3        VGIQGIAWAFGGMIFALVYCTAGISGGHINPAVTFGLFLARKLSLTRALFYMVMQCLGAI
RcPIP1;4        VGTQGIAWAFGGMIFALVYCTAGISGGHINPAVTFGLFLARKLSLTRALFYMVMQCLGAI
RcPIP1;5        VGVQGIAWAFGGMIFALVYCTAGISGGHINPAVTFGLLLARKLSLNRAIFYMVMQCLGAI
RcNIP5;1        ETLIGNAACAGLAVMIIILSTGHISGAHLNPSLTIAFAALRHFPWVQVPAYIAAQVSASI
HvPIP1;3        VGIQGIAWSFGGMIFVLVYCTAGISGGHINPAVTFGLFLARKLSLTRAVFYIVMQCLGAI
HvPIP1;4        VGIQGIAWSFGGMIFVLVYCTAGISGGHINPAVTFGLFLARKLSLTRAVFYIVMQCLGAI
ZmPIP1;1        VGIQGIAWSFGGMILALVYCTAGISG-HINPAVTFGLFLARKLSLTRAVFYIIMQCLGAI
OsNIP2;1        ISQLGQSIAGGLIVTVMIYAVGHISGAHMNPAVTLAFAVFRHFPWIQVPFYWAAQFTGAI
AtNIP5;1        ETLIGNAACAGLAVMIIILSTGHISGAHLNPSLTIAFAALRHFPWAHVPAYIAAQVSASI
AtNIP6;1        ETLIGCAASAGLAVMIVILSTGHISGAHLNPAVTIAFAALKHFPWKHVPVYIGAQVMASV
NtXIP1;1α       MPNLIMSILIAIVITILLLAVVPVSGGHINPVISFSAALVGIISMSRAIIYMVAQCVGAI
NtXIP1;1β       MPNLIMSILIAIVITILLLAVVPVSGGHINPVISFSAALVGIISMSRAIIYMVAQCVGAI
StXIP1;1α       MPNLIMSILIAVVITILLLAVVPVSGGHINPVISFSAALVGIISMSRAIIYIVAQCLGAV
StXIP1;1β       MPNLIMSILIAVVITILLLAVVPVSGGHINPVISFSAALVGIISMSRAIIYIVAQCLGAV
                      :   .  :  :: ..  :** *:** :::.      :.  :.  *   *  .::
```

```
RcPIP1;1        CGAGVVKALEKGHEYERLGGG-----------ANTVSSGYSKGDGLGAEIVGTFILVYTV
RcPIP1;2        CGAGVVKGFEGSHDYTRLGGG-----------ANSVNPGYTKGDGLGAEIVGTFVLVYTV
RcPIP1;3        CGAGVVKGFEGSHDYTRLGGG-----------ANSVNPGYTKGDGLGAEIVGTFVLVYTV
RcPIP1;4        CGAGVVKGFEGDRTYETLGGG-----------ANVVNAGYTKGDGLGAEIVGTFVLVYTV
RcPIP1;5        CGAGVVKGFQP-TPYERVGGG-----------ANMVNPGYSKGDGLGAEIVGTFVLVYTV
RcNIP5;1        CASFALKGVFHPFMSG-----------------GVTVPSVSTGQAFALEFLITFNLLFVV
HvPIP1;3        CGAGVVKGFQT-TLYQGNGGG-----------ANSVAAGYTKGDGLGAEIVGTFVLVYTV
HvPIP1;4        CGAGVVKGFQT-TLYQGNGGG-----------ANSVAAGYTKGDGLGAEIVGTFVLVYTV
ZmPIP1;1        CGRGVVKGFQQ-GLYMGNGGR-----------RNVVAPGYTKGDGLGAEIVGTFILVYTV
OsNIP2;1        CASFVLKAVIHPVDVI-----------------GTTTPVGPHWHSLVVEVIVTFNMMFVT
AtNIP5;1        CASFALKGVFHPFMSG-----------------GVTIPSVSLGQAFALEFIITFILLFVV
AtNIP6;1        SAAFALKAVFEPTMSG-----------------GVTVPTVGLSQAFALEFIISFNLMFVV
NtXIP1;1α       LGALALKAVVSSTIAQTFSLGGCTITVIAPGPNGPITVGLEMAQALWLEIFCTFVFLFAS
NtXIP1;1β       LGALALKAVVSSTIAQTFSLGGCTITVIAPGPNGPITVGLEMAQALWLEIFCTFVFLFAS
StXIP1;1α       LGALALRAVVSSSIEDTFSLGGCTVTIIAPGPNGPVTVGLETAQALWLEIFCTFVFLFAS
StXIP1;1β       LGALALRAVVSSSIEDTFSLGGCTVTIIAPGPNGPVTVGLETAQALWLEIFCTFVFLFAS
                 .  .::..                        .         ..:  *.. :* :::.
```

```
RcPIP1;1        FSATDAKRNARDS---HVPILAPLPIGFAVFLVHLATIP--ITGTGINPARSLGAALIYN
RcPIP1;2        FSATDAKRSARDS---HVPILAPLPIGFAVFLVHLATIP--ITGTGINPARSLGAAIIFN
RcPIP1;3        FSATDAKRSARDS---HVPILAPLPIGFAVFLVHLATIP--ITGTGINPARSLGAAIIFN
RcPIP1;4        FSATDAKRNARDS---HVPILAPLPIGFAVFLVHLATIP—ITGTGINPARSLGAAIIFN
RcPIP1;5        FSATDAKRSARDS---HVPVLAPLPIGFAVFLVHLATIP--ITGTGINPARSLGAAIIYN
RcNIP5;1        TAVATDTR--------AVGELAGIAVGATVMLNILVAGP--SSGGSMNPVRTLGPAVAAG
HvPIP1;3        FSATDAKRSARDS---HVPILAPLPIGFAVFLVHLATIP--ITGTGINPARSLGAAIIYN
HvPIP1;4        FSATDAKRSARDS---HVPILAPLPIGFAVFLVHLATIP--ITGTGINPARSLGAAIIYN
ZmPIP1;1        FSATDAKRRARDS---HVPILAPLPIGFAVFLVHLATMG--ITGTGINPARSLGAAVIYN
OsNIP2;1        LAVATDTR--------AVGELAGLAVGSAVCITSIFAGA--ISGGSMNPARTLGPALASN
AtNIP5;1        TAVATDTR--------AVGELAGIAVGATVMLNILVAGP--STGGSMNPVRTLGPAVASG
AtNIP6;1        TAVATDTR--------AVGELAGIAVGATVMLNILIAGP--ATSASMNPVRTLGPAIAAN
NtXIP1;1α       IWMAYDHRQAKALGLVTVLSIVGIVLGLLVFISTTVTMKKGYAGAGMNPARCFGAAVVRG
NtXIP1;1β       IWMAYDHRQAKALGLVTVLSIVGIVLGLLVFISTTVTMKKGYAGAGMNPARCFGAAVVRG
StXIP1;1α       IWMAYDHRQAKALGHVTVLSIVGLVLGLLVFISTTVTAKKGYGGAGINPARCLGPAIIRG
StXIP1;1β       IWMAYDHRQAKALGHVTVLSIVGLVLGLLVFISTTVTAKKGYGGAGINPARCLGPAIIRG
                   :   *         *  :. : :*  * :    :      . .:**.* :*.*:  .
```

Carbon dioxide (CO_2) Transporters

```
RcPIP1;1        GGHINPAVTFGLFLARKLSLTRAVFYMIMQCLGAICGAGVVKALEKGHEYERLGGGANTV
RcPIP1;3        GGHINPAVTFGLFLARKLSLTRALFYMVMQCLGAICGAGVVKGFEGSHDYTRLGGGANSV
RcPIP1;4        GGHINPAVTFGLFLARKLSLTRALFYMVMQCLGAICGAGVVKGFEGDRTYETLGGGANVV
RcPIP1;5        GGHINPAVTFGLLLARKLSLNRAIFYMVMQCLGAICGAGVVKGFQ-PTPYERVGGGANMV
RcPIP2;2        GGHINPAVTFGLFLARKVSLVRAIMYMVAQCLGAICGVGLVKAFQ-SSHYKRYGGGANTL
RcPIP2;4        GGHINPAVTFGLFLARKVSLIRAVAYMVAQCLGAICGVGLVKAFM-KNPYNRLGGGANSV
AtPIP1;2        GGHINPAVTFGLFLARKLSLTRAVYYIVMQCLGAICGAGVVKGFQ-PKQYQALGGGANTI
NtAQP1          GGHINPAVTFGLFLARKLSLTRAIFYIVMQCLGAICGAGVVKGFM-VGPYQRLGGGANVV
HvPIP2;1        GGHINPAVTFGLFLARKVSLIRALLYIIAQCLGAICGVGLVKGFQ-SSYYVRYGGGANEL
                ************:****:** **: *:: ********.*:**.:     *   ***** :

RcPIP1;1        SSGYSKGDGLGAEIVGTFILVYTVFSATDAKRNARDSHVPILAPLPIGFAVFLVHLATIP
RcPIP1;3        NPGYTKGDGLGAEIVGTFVLVYTVFSATDAKRSARDSHVPILAPLPIGFAVFLVHLATIP
RcPIP1;4        NAGYTKGDGLGAEIVGTFVLVYTVFSATDAKRNARDSHVPILAPLPIGFAVFLVHLATIP
RcPIP1;5        NPGYSKGDGLGAEIVGTFVLVYTVFSATDAKRSARDSHVPVLAPLPIGFAVFLVHLATIP
RcPIP2;2        DDNYSTGVGLGAEIIGTFVLVYTVFSATDPKRSARDSHVPVLAPLPIGFAVFMVHLATIP
RcPIP2;4        ASGYSNGTALGAEIIGTFVLVYTVFSATDPKRSARDSHVPVLAPLPIGFAVFMVHLATIP
AtPIP1;2        AHGYTKGSGLGAEIIGTFVLVYTVFSATDAKRNARDSHVPILAPLPIGFAVFLVHLATIP
NtAQP1          NHGYTKGDGLGAEIIGTFVLVYTVFSATDAKRNARDSYVPILAPLPIGFAVFLVHLATIP
HvPIP2;1        SAGYSKGTGLAAEIIGTFVLVYTVFSATDPKRNARDSHIPVLAPLPIGFAVFMVHLATIP
                  .*:.* .*.***:***:**********.**.****::*:***********:*******

RcPIP1;1        ITGTGINPARSLGAALIYNKDQAWDDHWIFWVGPFIGAALAALYHQIVIRAIPFQSK---
RcPIP1;3        ITGTGINPARSLGAAIIFNKDQGWDDHWIFWVGPFIGAALAALYHQVVIRAIPFKKC---
RcPIP1;4        ITGTGINPARSLGAAIIFNKDHAWDDHWIFWVGPFIGAALAAVYHQIVIRAIPFKSRA--
RcPIP1;5        ITGTGINPARSLGAAIIYNKDHAWDDHWIFWVGPFIGAALAALYHQIIIRAIPFKARA--
RcPIP2;2        ITGTGINPARSLGAAVIYNQDKAWDDQWIFWVGPFIGAAIAAFYHQFILRAGAVKALGSF
RcPIP2;4        ITGTGINPARSFGAAVIYNNDKVWDDHWIFWVGPFVGALAAAAYHQYVLRAAGIKALGSF
AtPIP1;2        ITGTGINPARSLGAAIIFNKDNAWDDHWVFWVGPFIGAALAALYHVIVIRAIPFKSRS--
NtAQP1          ITGTGINPARSLGAAIIYNTDQAWDDHWIFWVGPFIGAALAAVYHQIIIRAIPFHKSS--
HvPIP2;1        ITGTGINPARSLGAAVIYNTDKAWDDQWIFWVGPLIGAAIAAAYHQYVLRASAAK-LGSY
                ***********:***:*:* *: ***:*:*****::**  ** **  ::**   :
```

H_2O_2 Transporters

```
RcPIP1;1        AFGGMIFALVYCTAGISGGHINPAVTFGLFLARKLSLTRAVFYMIMQCLGAICGAGVVKA
RcPIP1;2        AFGGMIFALVYCTAGISGGHINPAVTFGLFLARKLSLTRALFYMVMQCLGAICGAGVVKG
RcPIP1;3        AFGGMIFALVYCTAGISGGHINPAVTFGLFLARKLSLTRALFYMVMQCLGAICGAGVVKG
RcPIP1;4        AFGGMIFALVYCTAGISGGHINPAVTFGLFLARKLSLTRALFYMVMQCLGAICGAGVVKG
RcPIP1;5        AFGGMIFALVYCTAGISGGHINPAVTFGLLLARKLSLNRAIFYMVMQCLGAICGAGVVKG
RcPIP2;1        AFGGMIFILVYCTAGISGGHINPAVTFGLFLARKVSLVRAIMYMAAQCLGAICGCGLVKA
RcPIP2;2        AFGGMIFILVYCTAGISGGHINPAVTFGLFLARKVSLVRAIMYMVAQCLGAICGVGLVKA
RcPIP2;3        AFGGMIFILVYCTAGISGGHINPAVTFGLFLGRKVSLIRALGYMVAQCLGAICGCGLVKA
RcPIP2;4        AFGGMIFILVYCTAGISGGHINPAVTFGLFLARKVSLIRAVAYMVAQCLGAICGVGLVKA
RcPIP2;5        AFGAMIFVLVYCTAGISGGHINPAVTFGLLVARKVSLDRAVSYMISQCLGAICGVGLVKA
RcTIP5;1        ANAFALSSAVYIAANISGGHVNPAVTFSMAIGGHISVPTALFYWVSQLVASVMACLLLRV
RcNIP2;1        AGGLIVTVMIYAVGHVSGAHMNPAVTTAFAAVRHFPWKEVPYYAAAQLTGAISASFTLKV
RcNIP3;1        AWGVVLMALIYAVGHVSGAHFNPAVSIAFAAGRKFPWKHVPMYILAQVLGSTLASLTLRV
RcNIP4;2        TWGLIVAVMIYSVGHISGAHFNPAVTITSAIFHRFPMHEVPLYIVAQVMGSILASGTLAL
RcNIP5;1        CAGLAVMIIILSTGHISGAHLNPSLTIAFAALRHFPWVQVPAYIAAQVSASICASFALKG
RcXIP1;1        AVFFIAFLWLMVTVPLSGGLFSPAFSFIAALRGVITFVRALFYSLGQLLGALIAYLILKG
RcXIP1;2        VVFLIAFFWLLTTVPLSGGFFSPTFAFMAALSGVISFVRALFYCLGQCFGAIIGYMILKS
RcXIP1;3        AVFFIALFWLLPTVPLSGGFFSPTFTFMAALRGVISFTRALFYCLGQCLGAIIGYIILKS
AtPIP2;5        AFGGMIFILVYCTAGISGGHINPAVTFGLLLARKVTLVRAVMYMVAQCLGAICGVALVKA
AtPIP2;1        AFGGMIFILVYCTAGISGGHINPAVTFGLFLARKVSLPRALLYIIAQCLGAICGVGFVKA
AtPIP2;2        AFGGMIFILVYCTAGISGGHINPAVTFGLFLARKVSLIRAVLYMVAQCLGAICGVGFVKA
AtPIP2;4        AFGGMIFVLVYCTAGISGGHINPAVTVGLFLARKVSLVRTVLYIVAQCLGAICGCGFVKA
ZmPIP2;5        AFGGMIFILVYCTAGVSGGHINPAVTFGLFLARKVSLVRALLYIVAQCLGAICGVGLVKG
AtPIP2;7        AFGGMIFVLVYCTAGISGGHINPAVTFGLFLARKVSLVRALGYMIAQCLGAICGVGFVKA
NtXIP1;1α       LIAIVITILLLAVVPVSGGHINPVISFSAALVGIISMSRAIIYMVAQCVGAILGALALKA
NtXIP1;1β       LIAIVITILLLAVVPVSGGHINPVISFSAALVGIISMSRAIIYMVAQCVGAILGALALKA
StXIP1;1α       LIAVVITILLLAVVPVSGGHINPVISFSAALVGIISMSRAIIYIVAQCLGAVLGALALRA
StXIP1;1β       LIAVVITILLLAVVPVSGGHINPVISFSAALVGIISMSRAIIYIVAQCLGAVLGALALRA
SlXIP1;1α       LIAVVITILLLAVVPVSGGHINPVISFSAALVGIISMSRAIIYIVAQCVGAILGALALRA
SlXIP1;1β       LIAVVITILLLAVVPVSGGHINPVISFSAALVGIISMSRAIIYIVAQCVGAILGALALRA
AtTIP1;1        AHAFGLFVAVSVGANISGGHVNPAVTFGAFIGGNITLLRGILYWIAQLLGSVVACLILKF
AtTIP1;2        AHAFGLFVAVSVGANISGGHVNPAVTFGVLLGGNITLLRGILYWIAQLLGSVAACFLLSF
TgTIP1;1        AHAFALFVAVSVGANISGGHVNPAVTFGAFLGGNITLLRGVLYIIAQLLGSSVACLLLRF
TgTIP1;2        AHAFALFVAVSVGANISGGHVNPAVTFGASLGGNITLLRGVLYIIAQLLGSSVACLLLRF
AtTIP2;3        AHAFALFVGVSIAANISGGHLNPAVTLGLAIGGNITLITGFFYWIAQCLGSIVACLLLVF
AtNIP1;2        VWGLTVMVLVYSLGHISGAHFNPAVTIAFASCGRFPLKQVPAYVISQVIGSTLAAATLRL
                         :     :**. ..* .:        ..      *   *  .:  .   :
```

```
RcPIP1;1        LEKGHEYER--LGGGAN-TVSSG--------YSKGDGLGAEIVGTFILVYTVFSATDAKR
RcPIP1;2        FEGSHDYTR--LGGGAN-SVNPG--------YTKGDGLGAEIVGTFVLVYTVFSATDAKR
RcPIP1;3        FEGSHDYTR--LGGGAN-SVNPG--------YTKGDGLGAEIVGTFVLVYTVFSATDAKR
RcPIP1;4        FEGDRTYET--LGGGAN-VVNAG--------YTKGDGLGAEIVGTFVLVYTVFSATDAKR
RcPIP1;5        FQP-TPYER--VGGGAN-MVNPG--------YSKGDGLGAEIVGTFVLVYTVFSATDAKR
RcPIP2;1        FQR-AYYN--RYGGGAN-ELADG--------YSTGTGLGAEIIGTFVLVYTVFAATDPKR
RcPIP2;2        FQS-SHYK--RYGGGAN-TLDDN--------YSTGVGLGAEIIGTFVLVYTVFSATDPKR
RcPIP2;3        FQK-AYYN--RYGGGAN-ELADG--------YNKGTGLGAEIIGTFVLVYTVFSATDPKR
RcPIP2;4        FMK-NPYN--RLGGGAN-SVASG--------YSNGTALGAEIIGTFVLVYTVFSATDPKR
RcPIP2;5        FME-HDYINITLGGGAN-SVATG--------YSKGAALGAEIVGTFVLVYTVFSATDPKR
RcTIP5;1        AAVGQNLPT--------YIIAEE--------MTGFGASIVEGVLTFGLVYTVYA-AGDPR
RcNIP2;1        LLHPVKH---------IGTTSPS--------GSDFQALVMEIVVTFCMMFVTSAVATDTK
RcNIP3;1        LFNDLDD-----IEVTVTQYKDS--------TSDLEAIIWEFIITFILMFNILAVATDYR
RcNIP4;2        VV-DVNP-------KAYFGTVPV--------GSNWQSLIMEIIIITFLLMFVISGVTTDDR
RcNIP5;1        VFHPFMSG---------GVTVPS--------VSTGQAFALEFLITFNLLFVVTAVATDTR
RcXIP1;1        VMDPNMAHKYALAGCMVNGNGAG--------VSVGTALIIEILCTFMVLYVAMTIILDKQ
RcXIP1;2        VMDPTIAHKYALGGCMVNGGGEG--------VSAGTALMVEFSCTFLVLYVAMTIILDKK
RcXIP1;3        VMDPTIAHKYALGGCMVNGNGEG--------VSAGTALMIEFSCTFLVLYTAMTIVLDKK
AtPIP2;5        FQS-AYFT--RYGGGAN-GLSDG--------YSIGTGVAAEIIGTFVLVYTVFSATDPKR
AtPIP2;1        FQS-SYYT--RYGGGAN-SLADG--------YSTGTGLAAEIIGTFVLVYTVFSATDPKR
AtPIP2;2        FQS-SYYD--RYGGGAN-SLADG--------YNTGTGLAAEIIGTFVLVYTVFSATDPKR
AtPIP2;4        FQS-SYYT--RYGGGAN-ELADG--------YNKGTGLGAEIIGTFVLVYTVFSATDPKR
ZmPIP2;5        FQS-AFYV--RYGGGAN-ELSAG--------YSKGTGLAAEIIGTFVLVYTVFSATDPKR
AtPIP2;7        FMK-TPYN--TLGGGAN-TVADG--------YSKGTALGAEIIGTFVLVYTVFSATDPKR
NtXIP1;1α       VVSSTIAQTFSLGGCTITVIAPGPNGPITVGLEMAQALWLEIFCTFVFLFASIWMAYDHR
NtXIP1;1β       VVSSTIAQTFSLGGCTITVIAPGPNGPITVGLEMAQALWLEIFCTFVFLFASIWMAYDHR
StXIP1;1α       VVSSSIEDTFSLGGCTVTIIAPGPNGPVTVGLETAQALWLEIFCTFVFLFASIWMAYDHR
StXIP1;1β       VVSSSIEDTFSLGGCTVTIIAPGPNGPVTVGLETAQALWLEIFCTFVFLFASIWMAYDHR
SlXIP1;1α       VVSSSIEDTFSLGGCTVTIIAPGPNGPVIVGLETAQALWLEIFCTFVFLFASIWMAYDHR
SlXIP1;1β       VVSSSIEDTFSLGGCTVTIIAPGPNGPVIVGLETAQALWLEIFCTFVFLFASIWMAYDHR
AtTIP1;1        ATGGLAVPA--------FGLSAG--------VGVLNAFVFEIVMTFGLVYTVYATAIDPK
AtTIP1;2        ATGGEPIPA--------FGLSAG--------VGSLNALVFEIVMTFGLVYTVYATAVDPK
TgTIP1;1        TTG-LGTGT--------FGLVAG--------VSVWSGLVMEIVMTFGLVYTVYATAVDPK
TgTIP1;2        TTG-LGTGT--------FGLVAG--------VSVWSGLVMEIVMTFGLVYTVYATAVDPK
AtTIP2;3        VTNGKSVPT--------HGVSAG--------LGAVEGVVMEIVVTFALVYTVYATAADPK
AtNIP1;2        LF-GLDQDVCSGKHDVFVGTLPS--------GSNLQSFVIEFIITFYLMFVISGVATDNR
                                                    .   *   ** .::         :
```

```
RcPIP1;1        NARDS---HVPILAPLPIGFAVFLVHLATIP--ITGTGINPARSLGAALIYNKDQAWDDH
RcPIP1;2        SARDS---HVPILAPLPIGFAVFLVHLATIP--ITGTGINPARSLGAAIIFNKDQGWDEH
RcPIP1;3        SARDS---HVPILAPLPIGFAVFLVHLATIP—-ITGTGINPARSLGAAIIFNKDQGWDDH
RcPIP1;4        NARDS---HVPILAPLPIGFAVFLVHLATIP--ITGTGINPARSLGAAIIFNKDHAWDDH
RcPIP1;5        SARDS---HVPVLAPLPIGFAVFLVHLATIP--ITGTGINPARSLGAAIIYNKDHAWDDH
RcPIP2;1        NARDS---HVPVLAPLPIGFAVFMVHLATIP--VTGTGINPARSFGAAVIYNKEKAWDDQ
RcPIP2;2        SARDS---HVPVLAPLPIGFAVFMVHLATIP--ITGTGINPARSLGAAVIYNQDKAWDDQ
RcPIP2;3        SARDS---HVPVLAPLPIGFAVFMVHLATIP--ITGTGINPARSFGAAVIYNKEKAWDDQ
RcPIP2;4        SARDS---HVPVLAPLPIGFAVFMVHLATIP--ITGTGINPARSFGAAVIYNNDKVWDDH
RcPIP2;5        KARDS---HVPILAPLPIGFAVFAVHLATIP--ITGTGINPARSLAAAIIYNTRKVWEEH
RcTIP5;1        R------SLLGATGPLVIGLMAGANVLAAGP--FSGGSMNPACAFGSAVVAG---RFKNQ
RcNIP2;1        --------AIGELAGIAVGSAVCITSILAGP--ISGGSMNPARTLGPAIASA---YYKGI
RcNIP3;1        --------AVKYLSGVAIGGTLLFNALLAGP--ITGASMNPARSLGPAIVSG---VYKNL
RcNIP4;2        T-------TAGPLGGIGVGMTILLNVFVAGP--VSGASMNPARSIGPAIVKH---VYKGL
RcNIP5;1        --------AVGELAGIAVGATVMLNILVAGP--SSGGSMNPVRTLGPAVAAG---NYRAL
RcXIP1;1        KCMDLGLTTVCVIISGIYAASVFVSITVTGRPGYGGVGLNPARCLGPVLLMG-GALWEGH
RcXIP1;2        KCQELGLTMVCVIISGAYAVSVFVSTTVTGRIGYGGVGLNPARCLAPALLLG-GSLWDGH
RcXIP1;3        KCQDLGLTMVCIIISGAYAVSVFVSTTVTGRVGYGGVGLNPARCLAPALLLG-GALWDGH
AtPIP2;5        SARDS---HVPVLAPLPIGFAVFIVHLATIP--ITGTGINPARSLGAAIIYNKDKAWDHH
AtPIP2;1        SARDS---HVPVLAPLPIGFAVFMVHLATIP--ITGTGINPARSFGAAVIYNKSKPWDDH
AtPIP2;2        NARDS---HVPVLAPLPIGFAVFMVHLATIP--ITGTGINPARSFGAAVIYNKSKPWDDH
AtPIP2;4        NARDS---HVPVLAPLPIGFAVFMVHLATIP--ITGTGINPARSFGAAVIYNNEKAWDDQ
ZmPIP2;5        NARDS---HVPVLAPLPIGFAVFMVHLATIP--ITGTGINPARSLGAAVIYNNDKAWDDH
AtPIP2;7        SARDS---HIPVLAPLPIGFAVFMVHLATIP--ITGTGINPARSFGAAVIYNNEKAWDDQ
NtXIP1;1α       QAKALGLVTVLSIVGIVLGLLVFISTTVTMKKGYAGAGMNPARCFGAAVVRG-GHLWDGH
NtXIP1;1β       QAKALGLVTVLSIVGIVLGLLVFISTTVTMKKGYAGAGMNPARCFGAAVVRG-GHLWDGH
StXIP1;1α       QAKALGHVTVLSIVGLVLGLLVFISTTVTAKKGYGGAGINPARCLGPAIIRG-GHLWDGH
StXIP1;1β       QAKALGHVTVLSIVGLVLGLLVFISTTVTAKKGYGGAGINPARCLGPAIIRG-GHLWDGH
SlXIP1;1α       QAKALGHVTVLSIVGLVLGLLVFISTTVTAKKGYGGAGINPARCLGPAIIRG-GHLWDGH
SlXIP1;1β       QAKALGHVTVLSIVGLVLGLLVFISTTVTAKKGYGGAGINPARCLGPAIIRG-GHLWDGH
AtTIP1;1        N------GSLGTIAPIAIGFIVGANILAGGA--FSGASMNPAVAFGPAVVSW---TWTNH
AtTIP1;2        N------GSLGTIAPIAIGFIVGANILAGGA--FSGASMNPAVAFGPAVVSW---TWTNH
TgTIP1;1        K------GDIGTIAPIAIGFIVGANILVGGA--FTGASMNPAIAFGPALVSW---SWTSH
TgTIP1;2        K------GDIGTIAPIAIGFIVGANILVGGA--FTGASMNPAIAFGPALVSW---SWASH
AtTIP2;3        K------GSLGTIAPIAIGFIVGANILAAGP--FSGGSMNPARSFGPAVVSG---DLSQI
AtNIP1;2        --------AIGELAGLAVGSTVLLNVIIAGP--VSGASMNPGRSLGPAMVYS---CYRGL
                                  .                * .:**   :...:
```

```
RcPIP1;1        WIFWVGPFIGAALAALYHQIVIRAIPFQSK------------------------------
RcPIP1;2        WIFWVGPFIGAALAALYHQVVIRAIPFKK-------------------------------
RcPIP1;3        WIFWVGPFIGAALAALYHQVVIRAIPFKKC------------------------------
RcPIP1;4        WIFWVGPFIGAALAAVYHQIVIRAIPFKSRA-----------------------------
RcPIP1;5        WIFWVGPFIGAALAALYHQIIIRAIPFKARA-----------------------------
RcPIP2;1        WIFWVGPFIGAAIAAIYHQYVLRASAAKALGSFRSSSNI---------------------
RcPIP2;2        WIFWVGPFIGAAIAAFYHQFILRAGAVKALGSFRSNPTV---------------------
RcPIP2;3        WIFWVGPFIGAAIAAFYHQYILRAAAIKALGSFRSNA-----------------------
RcPIP2;4        WIFWVGPFVGALAAAAYHQYVLRAAGIKALGSFRSNPTN---------------------
RcPIP2;5        WIFWVGPLVGALLAAVYYQYVIKGGAIKSLGSFRSSNLM---------------------
RcTIP5;1        AVYWVGPLLGATVAGLLYDNVVFPNQVPDSIRGISDGVGA--------------------
RcNIP2;1        WVYIVGPVVGTLLGSWSYNFIRVTDQPLQAISPRSFSAKLRRIRSTNEQPTNKDPFDAL-
RcNIP3;1        WVFIVSPIFGALAATYVYNMLR-VPEPEKSEEKT--KNIFNHLYTATDP-----------
RcNIP4;2        WVYIVGPIVGAILGASAYNLLR---SPYNQTP----------------------------
RcNIP5;1        WIYLVAPTLGAIAGAGTYSAVKLREEEVDP---------PRPVRSFRR------------
RcXIP1;1        WVFWVGPFCACMIYYAYSLMLPKRGFVRADIEEDIIKLVRASCSGSDCPSCVEKKVTSLY
RcXIP1;2        WVFWVGPICSCSVYYVFSLLLPKQGFVRADEQQHLIQLVRSSCLGSESPDYFEGKV----
RcXIP1;3        WVFWVGPVCACSVYYVCSLMLPKQGFVRADEEQHVLQLVRSSCLGSESANYFEGKV----
AtPIP2;5        WIFWVGPFAGAAIAAFYHQFVLRAGAIKALGSFRSQPHV---------------------
AtPIP2;1        WIFWVGPFIGAAIAAFYHQFVLRASGSKSLGSFRSAANV---------------------
AtPIP2;2        WIFWVGPFIGAAIAAFYHQFVLRASGSKSLGSFRSAANV---------------------
AtPIP2;4        WIFWVGPMIGAAAAAFYHQFILRAAAIKALGSFGSFGSFRSFA-----------------
ZmPIP2;5        WIFWVGPFIGAAIAAAYHQYVLRASAAK-LGSSASFSR----------------------
AtPIP2;7        WIFWVGPFLGALAAAAYHQYILRASAIKALGSFRSNATN---------------------
NtXIP1;1α       WIFWVGPTIACVAFYVYTKIIPPKHFHAD-GYKYDFIGVVKASFGLHE------------
NtXIP1;1β       WIFWVGPTIACVAFYVYTKIIPPKHFHAD-GYKYDFIGVVKASFGLHE------------
StXIP1;1α       WIFWVGPTIGCVAFYVYTKIIPPKHFLAEYGFKHDFVGVVKALSNV--------------
StXIP1;1β       WIFWVGPTIGCVAFYVYTKIIPPKHFLAEYGFKHDFVGVVKALSNV--------------
SlXIP1;1α       WIFWVGPTIGCVAFYVYTKIIPTKHFLAEYGFKHDFVGVVKALSNV--------------
SlXIP1;1β       WIFWVGPTIGCVAFYVYTKIIPTKHFLAEYGFKHDFVGVVKALSNV--------------
AtTIP1;1        WVYWAGPLVGGGIAGLIYEVFFINT-THEQLPTTDY------------------------
AtTIP1;2        WVYWAGPLIGGGLAGIIYDFVFIDENAHEQLPTTDY------------------------
TgTIP1;1        WVYWVGPLIGGGIAGIVYDLFFINS-THEQLPSTDY------------------------
TgTIP1;2        WVYWVGPLIGGGIAAIVYDLFFISG-THEQLPTTDY------------------------
AtTIP2;3        WIYWVGPLVGGALAGLIYGDVFIGS--YEAVETREIRV----------------------
AtNIP1;2        WIYIVSPIVGAVSGAWVYNMVRYTDKPLREITKS--GSFLKTVRNGSSR-----------
                 :: ..*  .          .
```

Silicic Acid Transporters

```
RcNIP2;1        VRHFPWKEVPYYAAAQLTGAISASFTLKVLLHPVKHIGTTSPSGSDFQALVMEIVVTFCM
HvNIP2;1        FRHFPWIQVPFYWAAQFTGAICASFVLKAVLHPITVIGTTEPVGPHWHALVIEVVVTFNM
TaLsi1          FRHFPWIQVPFYWAAQFTGAICASFVLKAVLHPITVIGTTEPVGPHWHALVIEVVVTFNM
ZmNIP2;1        FRHFPWIQVPFYWAAQFTGSICASFVLKAVLHPIAVLGTTTPTGPHWHSLVIEIIVTFNM
OsNIP2;1        FRHFPWIQVPFYWAAQFTGAICASFVLKAVIHPVDVIGTTTPVGPHWHSLVVEVIVTFNM
ZmNIP2;2        FRHFPWIQVPFYWAAQFTGAMCAAFVLKAVLHPIAVIGTTTPSGPHWHALLIEIVVTFNM
OsNIP2;2        FRHFPWIQVPFYWAAQFTGAMCAAFVLRAVLYPIEVLGTTTPTGPHWHALVIEIVVTFNM
CmNIP2;1a       TRHFPWKQVPLYAAAQLSGATCAAFTLRLLLHPIKHLGTTTPSGSDLQALVMEIVVTFSM
CpNIP1          TRHFPWKQVPLYAAAQLSGATCAAFTLRLLLHPIKHLGTTTPSGSDLQALVMEIVVTFSM
CmNIP2;1b       TRHFPWKQVPLYAAAQLSGATCAAFTLRLLLHPIKHLGTTTPSGSDLQALVMEIVVTFSM
GmNIP2;1        VRHLPWPQLPFYIAAQLTGAISASYTLRELLRPSNEIGGTSPAGSHIQALIMEMVTTYTM
GmNIP2;2        VRHLPWPQLPFYVAAQLTGAISASYTLRELLRPSDEIGGTSPAGSHIQALIMEMVSTYTM
                 **:** ::* * ***::*: .*::.*: :: *   :* * * *.. ::*::*:: *: *

RcNIP2;1        MFVTSAVATDTKAIGELAGIAVGSAVCITSILAGPISGGSMNPARTLGPAIASAYYKGIW
HvNIP2;1        MFVTLAVATDTRAVGELAGLAVGSSVCITSIFAGAVSGGSMNPARTLGPALASNRYPGLW
TaLsi1          MFVTLAVATDTRAVGELAGLAVGSSVCITSIFAGAVSGGSMNPARTLGPALASNRYPGLW
ZmNIP2;1        MFVTLAVATDTRAVGELAGLAVGSAVCITSIFAGAVSGGSMNPARTLGPALASNLYTGLW
OsNIP2;1        MFVTLAVATDTRAVGELAGLAVGSAVCITSIFAGAISGGSMNPARTLGPALASNKFDGLW
ZmNIP2;2        MFVTCAVATDSRAVGELAGLAVGSAVCITSIFAGPVSGGSMNPARTLAPAVASNVFTGLW
OsNIP2;2        MFVTCAVATDSRAVGELAGLAVGSAVCITSIFAGPVSGGSMNPARTLAPAVASNVYTGLW
CmNIP2;1a       MFVTCAVATDTKAVGELAGLAVGSAVCITSILAGPVSGGSMNPVRTLGPAMASDNYKGLW
CpNIP1          MFVTCAVATDTKAVGELAGLAVGSAVCITSILAGPVSGGSMNPVRTLGPAMASDNYKGLW
CmNIP2;1b       MFVTCAVATDTKAVGELAGLAVGSAVCITSILAGPVSGGSMNPVRTLGPAMASDNYKGLW
GmNIP2;1        VFISMAVATDSNATGQLSGVAVGSSVCIASIVAGPISGGSMNPARTLGPAIATSYYKGLW
GmNIP2;2        VFISMAVATDSNATGQLSGVAVGSSVCIASIVAGPISGGSMNPARTLGPAIATSYYKGLW
                :*:: *****:.* *:*:*:****:***:**.**.:*******.***.**:*:  : *:*

RcNIP2;1        VYIVGPVVGTLLGSWSYNFIRVTDQPLQAIS--PRSFS-AKLRRIRST-NEQPTNKDPFD
HvNIP2;1        LYFLGPVLGTLSGAWTYTYIRFEDPP--KDA--PQKLSSFKLRRLQSQ-SVAADD-DELD
TaLsi1          LYFLGPVLGTLSGAWTYTYIRFEDPP--KDG--PQKLSSFKLRRLQSQ-SVAADD-DELD
ZmNIP2;1        IYFLGPVLGTLSGAWTYTYIRFEEAPSHKDM--SQKLSSFKLRRLQSQ-SVAVDD-DELD
OsNIP2;1        IYFLGPVMGTLSGAWTYTFIRFEDTP-KEGS--SQKLSSFKLRRLRSQQSIAADDVDEME
ZmNIP2;2        IYFLGPVIGTLSGAWVYTYIRFEEAPAAKD---TQRLSSFKLRRMQSQ--LAADEFDTV-
OsNIP2;2        IYFLGPVVGTLSGAWVYTYIRFEEAPAAAGGAAPQKLSSFKLRRLQSQ-SMAADEFDNV-
CmNIP2;1a       VYFVGLVTGTLLGAWSYKFIRASDKPVHLIS--PHSFS-LKLRRMSRS-DVGEGER----
CpNIP1          VYFVGPVTGTLLGAWSYKFIRASDKPVHLIS--PHSFS-LKLRRMSRS-DVGEGER----
CmNIP2;1b       VYFVGPVTGTLLGAWSYKFIRASDKPVHLIS--PHSFS-LKLRRMSRS-DVGEGER----
GmNIP2;1        VYFVGPITGAVLAAWSYNVIRDTEHPGFPIS--LSSIS-SKVRQSIGGTEQKSDQRCLV-
GmNIP2;2        VYFVGPITGAVLAAWSYNVIRDTEHPGFPIS--LSSIS-SKVRQSIGGTEQKSDQRCLV-
                :*::* : *:: .:* *. **  : *          :*  *:*:          :
```

Urea Transporters

```
RcPIP1;1        SK------CSTVGIQGIAWAFGGMIFALVYCTAGISGGHINPAVTFGLFLARKLSLTRAV
RcPIP1;2        TK------CSTVGIQGIAWAFGGMIFALVYCTAGISGGHINPAVTFGLFLARKLSLTRAL
RcPIP1;3        TK------CSTVGIQGIAWAFGGMIFALVYCTAGISGGHINPAVTFGLFLARKLSLTRAL
RcPIP1;4        NK------CATVGTQGIAWAFGGMIFALVYCTAGISGGHINPAVTFGLFLARKLSLTRAL
RcPIP1;5        NK------CASVGVQGIAWAFGGMIFALVYCTAGISGGHINPAVTFGLLLARKLSLNRAI
RcPIP2;1        DPAKNADACGGVGILGIAWAFGGMIFILVYCTAGISGGHINPAVTFGLFLARKVSLVRAI
RcPIP2;3        DPDKNADACGGVGILGIAWAFGGMIFILVYCTAGISGGHINPAVTFGLFLGRKVSLIRAL
RcPIP2;2        E----SDSCGGVGILGIAWAFGGMIFILVYCTAGISGGHINPAVTFGLFLARKVSLVRAI
RcPIP2;4        DP------CGGVGILGIAWAFGGMIFILVYCTAGISGGHINPAVTFGLFLARKVSLIRAV
RcPIP2;5        SP------CDGVGILGIAWAFGAMIFVLVYCTAGISGGHINPAVTFGLLVARKVSLDRAV
RcTIP1;1        NNG----AATPSGLVAAAIAHAFALFVAVSVGANISGGHVNPAVTFGAFVGGNITLLRGI
RcTIP1;2        DGG----STTPSGLVAASLSHAFALFVAVSVGANISGGHVNPAVTFGAFIGGHITLLRGI
RcTIP1;3        SDG----ATTPAGLVAASLAHGFALFVAVSVGANISGGHVNPAVTFGAFVGGHITFIRSI
RcTIP1;4        DDG----SSTPAAIIMASLAHAFGLFVGVSTAANISGGHCNPAVTFGAFLGGNISLLRGI
RcTIP2;1        SDA----ALDPAGLVAIAICHGFALFVAVAVGANISGGHVNPAVTFGLALGGQITILTGI
RcTIP2;2        TDA----ALDPPGLVAVAVAHAFGLFVGVAIAANISGGHLNPAVTFGLAVGGNITILTGI
RcTIP3;1        RET----GPPASGLVMVALAHGLALFSAVSASINISGGHLNPAVTFGALVGGRISVVLAF
RcTIP4;1        GD-------SLVGLFFVAMAHTLVVAVMISAG-HISGGHLNPAVTLGLLAGGHITVVRSI
RcTIP5;1        P------AADPSNLVIVALANAFALSSAVYIAANISGGHVNPAVTFSMAIGGHISVPTAL
RcNIP1;1        KV---------VTLPGISIVWGLAVMVLVYSVGHISGAHFNPAVTLAFATCKRFPWKQVP
RcNIP2;1        KR---------ISKLGASLAGGLIVTVMIYAVGHVSGAHMNPAVTTAFAAVRHFPWKEVP
RcNIP3;1        -----------LTMVGIAIAWGVVLMALIYAVGHVSGAHFNPAVSIAFAAGRKFPWKHVP
RcNIP4;1        -S---------VTFPGICVVWGLIVMVMVYSVGHISGAHFNPAVTITFAIFRQFPYKQVP
RcNIP4;2        -S---------VTFPGVCVTWGLIVAVMIYSVGHISGAHFNPAVTITSAIFHRFPMHEVP
RcNIP5;1        GV---------ETLIGNAACAGLAVMIIILSTGHISGAHLNPSLTIAFAALRHFPWVQVP
RcNIP6;1        GT---------ETLIGLAVSTGLAVMIVILSTGHISGAHLNPSITIAFAALRHFPWKHVP
RcNIP7;1        GQ---------VGLLEYAATAGLTVIVLVFAIGPISGAHVNPAVTIAFATFGHFPWSKVP
CpNIP1          QR---------VSQLGASVAGGLIVTVMIYAVGHISGAHMNPAVTTAFAATRHFPWKQVP
OsNIP2;1        SR---------ISQLGQSIAGGLIVTVMIYAVGHISGAHMNPAVTLAFAVFRHFPWIQVP
AtNIP6;1        GA---------ETLIGCAASAGLAVMIVILSTGHISGAHLNPAVTIAFAALKHFPWKHVP
NtAQP1          SL------CSSVGIQGVAWAFGGMIFALVYCTAGISGGHINPAVTFGLFLARKLSLTRAI
ZmPIP1;5        SK------CATVGIQGIAWSFGGMIFALVYCTAGISGGHINPAVTFGLFLARKLSLTRAL
AtTIP1;3        GDG----PATPAGLVAASLSHAFALFVAVSVGANVSGGHVNPAVTFGAFIGGNITLLRAI
AtTIP1;1        ENG----ATTPSGLVAAAVAHAFGLFVAVSVGANISGGHVNPAVTFGAFIGGNITLLRGI
AtTIP1;2        DNG----ATTPSGLVAAALAHAFGLFVAVSVGANISGGHVNPAVTFGVLLGGNITLLRGI
AtTIP2;1        SDA----ALDTPGLVAIAVCHGFALFVAVAIGANISGGHVNPAVTFGLAVGGQITVITGV
NtTIPa          GD-------PLVSLFFVAMAHALVVAVTISAGFRISGGHLNPAVTLGLCMGGHITVFRSI
AtTIP4;1        GN-------TLVGLFAVAVAHAFVVAVMISAG-HISGGHLNPAVTLGLLLGGHISVFRAF
AtTIP5;1        AGD----VSGPFGVLIPAIANALALSSSVYISWNVSGGHVNPAVTFAMAVAGRISVPTAM
NtXIP1;1α       VK---------MPNLIMSILIAIVITILLLAVVPVSGGHINPVISFSAALVGIISMSRAI
NtXIP1;1β       VK---------MPNLIMSILIAIVITILLLAVVPVSGGHINPVISFSAALVGIISMSRAI
StXIP1;1α       TK---------MPNLIMSILIAVVITILLLAVVPVSGGHINPVISFSAALVGIISMSRAI
StXIP1;1β       TK---------MPNLIMSILIAVVITILLLAVVPVSGGHINPVISFSAALVGIISMSRAI
                                 .      :   :     :**.* ** ::        ..
```

```
RcPIP1;1        FYMIMQCLGAICGAGVVKALEKGHEYER--LGGGANTVSS---------GYSKGDGLGAE
RcPIP1;2        FYMVMQCLGAICGAGVVKGFEGSHDYTR--LGGGANSVNP---------GYTKGDGLGAE
RcPIP1;3        FYMVMQCLGAICGAGVVKGFEGSHDYTR--LGGGANSVNP---------GYTKGDGLGAE
RcPIP1;4        FYMVMQCLGAICGAGVVKGFEGDRTYET--LGGGANVVNA---------GYTKGDGLGAE
RcPIP1;5        FYMVMQCLGAICGAGVVKGFQP-TPYER--VGGGANMVNP---------GYSKGDGLGAE
RcPIP2;1        MYMAAQCLGAICGCGLVKAFQR-AYYN--RYGGGANELAD---------GYSTGTGLGAE
RcPIP2;2        MYMVAQCLGAICGVGLVKAFQS-SHYK--RYGGGANTLDD---------NYSTGVGLGAE
RcPIP2;3        GYMVAQCLGAICGCGLVKAFQK-AYYN--RYGGGANELAD---------GYNKGTGLGAE
RcPIP2;4        AYMVAQCLGAICGVGLVKAFMK-NPYN--RLGGGANSVAS---------GYSNGTALGAE
RcPIP2;5        SYMISQCLGAICGVGLVKAFME-HDYINITLGGGANSVAT---------GYSKGAALGAE
RcTIP1;1        LYWIAQLLGSTVACLLLKFSTG-------GLTTSAFALSS---------GVGVWNAFVFE
RcTIP1;2        LYWIAQLLGSVVACLLLKFSTG-------GLETSAFALSS---------GVSSWNAVVFE
RcTIP1;3        LYWIAQLLGSVVACLLLKFSTG-------GLETSAFALSS---------GVGAWNAVVFE
RcTIP1;4        LYWIAQLLGSTVACLLLKFSTH-------GMTTSAFALSS---------GVNVWNAFVFE
RcTIP2;1        FYWIAQLLGSIVACFLLKVVTG-------GLATPIHSVAA---------GVGAIEGVVME
RcTIP2;2        FYWIAQCLGSIVACLLLQFVTN-------GKSIPTHGVAS---------GMSAIEGVVME
RcTIP3;1        YYWIAQLLGAIVASLLLRLVTN-------GMRPVGFHVTS---------GVGEVHGLIME
RcTIP4;1        LYWIDQLLASSAACFLLNYLTG-------GMATPVHTLAS---------GVGYVQGIVWE
RcTIP5;1        FYWVSQLVASVMACLLLRVAAV-------GQNLPTYIIAE---------EMTGFGASIVE
RcNIP1;1        AYIACQVIGSTLAAGTIRLIFT----GKQDHFTGTMP------------AGSDMQSFVVE
RcNIP2;1        YYAAAQLTGAISASFTLKVLLH----PVKHIGTTSPS-------------GSDFQALVME
RcNIP3;1        MYILAQVLGSTLASLTLRVLFN----DLDDIEVTVTQYKD---------STSDLEAIIWE
RcNIP4;1        IYIVAQVVGSLLASGTLYYIFS----VTDEAFFGTVP------------VGPPMRSFVLE
RcNIP4;2        LYIVAQVMGSILASGTLALVVD----VNPKAYFGTVP------------VGSNWQSLIME
RcNIP5;1        AYIAAQVSASICASFALKGVFH----PFMSGGVTVPS-------------VSTGQAFALE
RcNIP6;1        VYIGAQVSASVCAAFALKVIFH----PFMSGGVTVPS-------------GGYGQAFALE
RcNIP7;1        FYVVAQTVGSVLATYAAKLVYG----IKADLMVTRPV-------------QGCNSAFSVE
CpNIP1          LYAAAQLSGATCAAFTLRLLLH----PIKHLGTTTPS-------------GSDLQALVME
OsNIP2;1        FYWAAQFTGAICASFVLKAVIH----PVDVIGTTTPV-------------GPHWHSLVVE
AtNIP6;1        VYIGAQVMASVSAAFALKAVFE----PTMSGGVTVPT-------------VGLSQAFALE
NtAQP1          FYIVMQCLGAICGAGVVKGFMV-GPYQR--LGGGANVVNH---------GYTKGDGLGAE
ZmPIP1;5        FYMVMQCLGAICGAGVVKGFQE-GLYMG--AGGGANAVNP---------GYTKGDGLGAE
AtTIP1;3        LYWIAQLLGAVVACLLLKVSTG-------GMETAAFSLSY---------GVTPWNAVVFE
AtTIP1;1        LYWIAQLLGSVVACLILKFATG-------GLAVPAFGLSA---------GVGVLNAFVFE
AtTIP1;2        LYWIAQLLGSVAACFLLSFATG-------GEPIPAFGLSA---------GVGSLNALVFE
AtTIP2;1        FYWIAQLLGSTAACFLLKYVTG-------GLAVPTHSVAA---------GLGSIEGVVME
NtTIPa          LYWIDQLLASVAACALLNYLTA-------GLETPVHTLAN---------GVSYGQGIIME
AtTIP4;1        LYWIDQLLASSAACFLLSYLTG-------GMGTPVHTLAS---------GVSYTQGIIWE
AtTIP5;1        FYWTSQMIASVMACLVLKVTVM-------EQHVPIYKIAG---------EMTGFGASVLE
NtXIP1;1α       IYMVAQCVGAILGALALKAVVSSTIAQTFSLGGCTITVIAPGPNGPITVGLEMAQALWLE
NtXIP1;1β       IYMVAQCVGAILGALALKAVVSSTIAQTFSLGGCTITVIAPGPNGPITVGLEMAQALWLE
StXIP1;1α       IYIVAQCLGAVLGALALRAVVSSSIEDTFSLGGCTVTIIAPGPNGPVTVGLETAQALWLE
StXIP1;1β       IYIVAQCLGAVLGALALRAVVSSSIEDTFSLGGCTVTIIAPGPNGPVTVGLETAQALWLE
                 *   *  .:  .                                          .   *
```

```
RcPIP1;4        IVGTFILVYTVFSATDAKRNA---RDSHVPILAPLPIGFAVFLVHLATIPI--TGTGINP
RcPIP1;1        IVGTFVLVYTVFSATDAKRSA---RDSHVPILAPLPIGFAVFLVHLATIPI--TGTGINP
RcPIP1;2        IVGTFVLVYTVFSATDAKRSA---RDSHVPILAPLPIGFAVFLVHLATIPI——TGTGINP
RcPIP1;3        IVGTFVLVYTVFSATDAKRNA---RDSHVPILAPLPIGFAVFLVHLATIPI--TGTGINP
RcPIP1;5        IVGTFVLVYTVFSATDAKRSA---RDSHVPVLAPLPIGFAVFLVHLATIPI--TGTGINP
RcPIP2;1        IIGTFVLVYTVFAATDPKRNA---RDSHVPVLAPLPIGFAVFMVHLATIPV--TGTGINP
RcPIP2;2        IIGTFVLVYTVFSATDPKRSA---RDSHVPVLAPLPIGFAVFMVHLATIPI--TGTGINP
RcPIP2;3        IIGTFVLVYTVFSATDPKRSA---RDSHVPVLAPLPIGFAVFMVHLATIPI--TGTGINP
RcPIP2;4        IIGTFVLVYTVFSATDPKRSA---RDSHVPVLAPLPIGFAVFMVHLATIPI--TGTGINP
RcPIP2;5        IVGTFVLVYTVFSATDPKRKA---RDSHVPILAPLPIGFAVFAVHLATIPI--TGTGINP
RcTIP1;1        IVMTFGLVYTVYATAVDPK------KGSLGTIAPIAIGFIVGANILAGGAF--DGASMNP
RcTIP1;2        IVMTFGLVYTVYATAVDPK------KGNIGIIAPIAIGFIVGANILAGGAF--DGASMNP
RcTIP1;3        IVMTFGLVYTVYATAVDPK------KGNIGIIAPLAIGFIVGANILAGGAF--DGASMNP
RcTIP1;4        IVMTFGLVYTVYATAIDPK------KSEVGIIAPLAIGFVVGANILAGGAF--EGASMNP
RcTIP2;1        IVITFALVYTVYATAADPK------KGSLGTIAPIAIGFIVGANILAAGPF--SGGSMNP
RcTIP2;2        IVITFALVYTVYATAADPK------KGDLGIIAPIAIGFIVGANILAAGPF--SGGSMNP
RcTIP3;1        MVMTFGLVYTVYATAIDPK------RGSLGIIAPLAIGFIVGANILVGGPF--DGASMNP
RcTIP4;1        IVLTFSLLFTVYATIVDPK------KGSIDGLGPTLTGFVVGANILAGGPF--SGASMNP
RcTIP5;1        GVLTFGLVYTVYA-AGDPR------RSLLGATGPLVIGLMAGANVLAAGPF--SGGSMNP
RcNIP1;1        FIITFYLMFIISGVATDNR--------AIGELAGLAVGATVLLNVMFAGPI--SGASMNP
RcNIP2;1        IVVTFCMMFVTSAVATDTK--------AIGELAGIAVGSAVCITSILAGPI--SGGSMNP
RcNIP3;1        FIITFILMFNILAVATDYR--------AVKYLSGVAIGGTLLFNALLAGPI--TGASMNP
RcNIP4;1        IIISFLLMFVISGVATDNR--------AIGELAGIAVGMTIMLNVFIAGPV--SGASMNP
RcNIP4;2        IIITFLLMFVISGVTTDDRT-------TAGPLGGIGVGMTILLNVFVAGPV--SGASMNP
RcNIP5;1        FLITFNLLFVVTAVATDTR--------AVGELAGIAVGATVMLNILVAGPS--SGGSMNP
RcNIP6;1        FIISFNLMFVVTAVATDTR--------AVGELAGIAVGATVTLNILIAGET--TGASMNP
RcNIP7;1        FITTFLMMFLAASLAYQAA---------TRHLSGFVIGLSIGLAVLISGPV--SGGSLNP
CpNIP1          IVVTFSMMFVTCAVATDTK--------AVGELAGLAVGSAVCITSILAGPV--SGGSMNP
OsNIP2;1        VIVTFNMMFVTLAVATDTR--------AVGELAGLAVGSAVCITSIFAGAI--SGGSMNP
AtNIP6;1        FIISFNLMFVVTAVATDTR--------AVGELAGIAVGATVMLNILIAGPA--TSASMNP
NtAQP1          IIGTFVLVYTVFSATDAKRNA---RDSYVPILAPLPIGFAVFLVHLATIPI--TGTGINP
ZmPIP1;5        IVGTFVLVYTVFSATDAKRSA---RDSHVPILAPLPIGFAVFLVHLATIPI--TGTGINP
AtTIP1;3        IVMTFGLVYTVYATAVDPK------KGDIGIIAPLAIGLIVGANILVGGAF--DGASMNP
AtTIP1;1        IVMTFGLVYTVYATAIDPK------NGSLGTIAPIAIGFIVGANILAGGAF--SGASMNP
AtTIP1;2        IVMTFGLVYTVYATAVDPK------NGSLGTIAPIAIGFIVGANILAGGAF--SGASMNP
AtTIP2;1        IIITFALVYTVYATAADPK------KGSLGTIAPLAIGLIVGANILAAGPF--SGGSMNP
NtTIPa          VILTFSLLFTVYTTIVDPK------KGILEGMGPLLTGLVVGANIMAGGPF--SGASMNP
AtTIP4;1        IILTFSLLFTVYATIVDPK------KGSLDGFGPLLTGFVVGANILAGGAF--SGASMNP
AtTIP5;1        GVLAFVLVYTVFT-ASDPR------RGLPLAVGPIFIGFVAGANVLAAGPF--SGGSMNP
NtXIP1;1α       IFCTFVFLFASIWMAYDHRQAKALGLVTVLSIVGIVLGLLVFISTTVTMKKGYAGAGMNP
NtXIP1;1β       IFCTFVFLFASIWMAYDHRQAKALGLVTVLSIVGIVLGLLVFISTTVTMKKGYAGAGMNP
StXIP1;1α       IFCTFVFLFASIWMAYDHRQAKALGHVTVLSIVGLVLGLLVFISTTVTAKKGYGGAGINP
StXIP1;1β       IFCTFVFLFASIWMAYDHRQAKALGHVTVLSIVGLVLGLLVFISTTVTAKKGYGGAGINP
                 . :* :::                               *                 . .:**
```
